# Evidence for metaviromic islands in marine phages

**DOI:** 10.3389/fmicb.2014.00027

**Published:** 2014-02-03

**Authors:** Carolina Megumi Mizuno, Rohit Ghai, Francisco Rodriguez-Valera

**Affiliations:** Evolutionary Genomics Group, Departamento de Producción Vegetal y Microbiología, Universidad Miguel HernándezAlicante, Spain

**Keywords:** population genomics, phages, metaviromes, host recognition, marine phages, virome

## Abstract

Metagenomic islands (MGIs) have been defined as genomic regions in prokaryotic genomes that under-recruit from metagenomes where most of the same genome recruits at close to 100% identity over most of its length. The presence of MGIs in prokaryotes has been associated to the diversity of concurrent lineages that vary at this level to disperse the predatory pressure of phages that, reciprocally, maintain high clonal diversity in the population and improve ecosystem performance. This was proposed as a Constant-Diversity (C-D) model. Here we have investigated the regions of phage genomes under-recruiting in a metavirome constructed with a sample from the same habitat where they were retrieved. Some of the genes found to under-recruit are involved in host recognition as would be expected from the C-D model. Furthermore, the recruitment of intragenic regions known to be involved in molecular recognition also had a significant under-recruitment compared to the rest of the gene. However, other genes apparently disconnected from the recognition process under-recruited often, specifically the terminases involved in packaging of the phage genome in the capsid and a few others. In addition, some highly related phage genomes (at nucleotide sequence level) had no metaviromic islands (MVIs). We speculate that the latter might be generalist phages with broad infection range that do not require clone specific lineages.

## Introduction

One of the major conundrums of microbiology is the population biology of bacteria and their phages (Rodriguez-Valera, 2002). Pure culture, the work-horse of bacteriology, imposes a strong prejudice about how bacteria really are in nature (Hugenholtz et al., 1998). Owing to their asexual reproduction the population in a pure culture is essentially clonal, composed of nearly identical cells. Likewise, in an infectious disease, typically the pathogen is a clone and repeated isolation of the same clone is at the root of modern epidemiology (Stenderup and Orskov, 1983). However, we have very little knowledge of what the situation is in natural environments such as soil or waters. One approach to study the population biology of a microbe is to isolate and compare many strains from the same sample (Tettelin et al., 2005; Jacobs-Sera et al., 2012). However, this approach is fraught with challenges. The isolation process can be heavily biased (Rodriguez-Valera, 2004) and the same clone may be retrieved many times. To get a realistic picture many strains might be required, and the cost of sequencing and analyzing their genomes may be disproportionate to the information gain.

On the other hand, high throughput metagenomics provides a composite of the individual cells present in a sample that can be gathered using the genome of one isolate for retrieval of the homologous regions in the clonal lineages present in the population. This virtual experiment has already been done repeatedly for cellular microbes revealing very interesting patterns (Coleman et al., 2006; Legault et al., 2006; Cuadros-Orellana et al., 2007; Rodriguez-Valera et al., 2009). Typically, a large part of the strain genome is covered at a very high identity. Actually, several 100% identical fragments are found for most of the core genome. The retrieval of identical fragments could have been expected, particularly when the isolate comes from the same sample as the metagenome. However, normally there is also a cloud of reads with similarities within the range from 100 to ca. 95% (Caro-Quintero and Konstantinidis, 2012). This similarity cloud reflects the natural variation of the clonal frames that coexist within the population (López-Pérez et al., 2013). Even less expected was the appearance of selected tracts of the reference genome that recruit much less and sometimes not at all. These regions have been named metagenomic islands (MGIs) and overlap mostly (but not completely) with the flexible genome (defined when comparing different strain genomes) (Coleman et al., 2006; Cuadros-Orellana et al., 2007; Pašić et al., 2009). Overall, these data can be explained by a high degree of concurrent genomic diversity in prokaryotic populations (Gonzaga et al., 2012; López-Pérez et al., 2013). The finding of such high diversity is contradictory to the classical paradigm derived from laboratory enrichments indicating that clonal sweeps happen regularly in prokaryotic populations narrowing the genetic diversity (Atwood et al., 1951; Cohan and Koeppel, 2008). Particularly, it raises the question of how selection events that favor a single high-fitness clone (clonal sweeps) are prevented from decreasing clonal diversity. Considering these issues and the high diversity of concurrent clonal lineages found in the environments analyzed, a model was proposed in which high clonal diversity was maintained by the predatory pressure exercised by bacteriophages (Rodriguez-Valera et al., 2009). The constant-diversity model (C-D) posits that several clonal lineages coexist, enriching the genetic wealth of the population, and these are kept under control by the stabilizing influence of a similar diversity of viruses that prey on the population. It is actually a revival of the classic “kill-the-winner” model proposed years ago (Thingstad and Lignell, 1997; Thingstad, 2000) to explain the plankton paradox, but this time applied to clonal frames within a single species, rather than to different species with similar niches.

One of the predictions of C-D is that diverse populations of phages preying on the same species coexist in a single environmental niche. Actually, the clonal diversity of phage populations has been also approached by isolating multiple phages and indicated that indeed many lineages coexist at a single time and place, for example, roseophages (Angly et al., 2009), cyanophages (Labrie et al., 2013), and *Alteromonas* phages (Garcia-Heredia et al., 2013) among others. However, drawbacks similar to those encountered in prokaryotic pure culture methods limit the weight of the evidence. Rodriguez-Brito et al. studied the diversity of phages in individual saltern ponds by a viral metagenomic approach and found evidence for a high diversity that was maintained through seasons and years, even though within a short time scale, rank switching could be detected (Rodriguez-Brito et al., 2010). Along similar lines, a high conserved diversity of phages has been detected in a hypersaline lake (Emerson et al., 2013). However, given the scarcity of phage genomes isolated from the same environments, a phage equivalent of the MGIs detected in bacteria and archaea has been hitherto missing. There is only the report of Garcia-Heredia et al, indicating that some host recognition genes (glucanases) under-recruited in metaviromes from hypersaline waters (Garcia-Heredia et al., 2012).

For prokaryotic genomes, the presence of MGIs has been interpreted as indicative of the presence of different clonal lineages with variations at some gene clusters diluting the recruiting efficiency of these regions. These gene clusters are diverse but include an abundance of outer cellular structures that are candidate recognition target of phages, such as the O-chain polysaccharide synthesis genes, that were always among the least recruiting in the genome. We wondered if the reciprocal was also true. That is, that the regions that were different among the viral lineages were involved in host recognition as some previous evidence seemed to indicate (for example, Angly et al., 2009). In this work, taking advantage of the availability of a large set of phage contigs from the Mediterranean Deep Chlorophyll Maximum (MedDCM), by cloning metagenomic DNA in fosmids and a metavirome from the same location (Mizuno et al., 2013), we examine patterns of variability and the presence of the viral equivalent of MGIs or metaviromic islands (MVIs) in the most abundant phages. The results show a remarkable degree of clonal diversity of concurrent phages, even more so than the prokaryotic host and affecting different genes, with some (but not all) involved in host recognition.

## Materials and methods

### Sampling, fosmid, and metavirome sequencing

The sampling, fosmid library construction, assembly and recovery of phage genomes has been described previously (Ghai et al., 2010; Mizuno et al., 2013). Briefly, a fosmid library of ~13000 clones was constructed from the DNA from a 0.22 μm filter from a sample from the Mediterranean DCM. The sampling date was October 15, 2007, depth 50 m and location was off the coast of Alicante, Spain (38°4′6.64″N 0°13′55.18″W). DNA from ~6000 metagenomic fosmids was sequenced in 24 batches using Illumina. Each batch was assembled independently. 1148 phage derived contigs were identified using sequence based approaches and classified into groups based on high sequence identity. Complete genomes of phages were termed as Complete Genome Representatives (CGRs) and incomplete contigs related to CGRs were termed Complete Genome Fragments (CGFs). All 1148 contigs assembled were submitted to DDBJ and are available using the accession numbers AP013358-AP014505.

Sampling and sequencing of metavirome MedDCM-Vir has also been described previously (Mizuno et al., 2013). Briefly, the metaviromic biomass originates from another sample of 20 L from the Mediterranean DCM (sampling date August 29, 2011, depth 65 m, from the same location as above). After filtration through 0.22 μm, phages were concentrated using tangential flow filtration (TFF) with a 30 kD polyethersulfone membrane and multiple amplification displacement (MDA) was performed. The resulting DNA was sequenced in one third of an Illumina lane. The metavirome has been deposited in NCBI SRA with the Bioproject number PRJNA210529.

### Selection of phage contigs and MVI detection

From a total of 1148 phage contigs described previously (Mizuno et al., 2013), we have focused on 208 that were identified as complete genomes (referred to as CGRs) and those highly related to them, albeit incomplete (524 Complete Genome Fragments, CGFs). The CGRs, by comparison to reference phage genomes were further classified into 21 groups (G1, G2 etc.) that are akin to high level taxa (such as family) (Mizuno et al., 2013). In order to reliably identify MVIs we first identified the most abundant CGRs and CGFs in a MDA-amplified metavirome obtained from the same habitat and location 4 years later (Mizuno et al., 2013). Fragment recruitment was done at very high nucleotide identity (>98% over at least 50 nucleotides) and only those with a minimum median base coverage of at least 5× and a value of reads per Kb of sequence per Gb of metavirome (RPKG) >1 were used for further analysis (total of 224 phage contigs from which 51 are CGRs and 173 CGFs). In these 224 selected contigs, regions that had a coverage of less than 20% of the median coverage of the entire contig and were >500 bp in length were considered as MVIs (Data S1).

### Projecting fragment recruitment on protein structure

The base coverages of each C1q gene were projected onto the amino acid sequence of the same using the average of the read coverage for each base for each codon (that is, data for each amino acid position was computed by averaging the read coverages of all three bases in the codon). Protein structure prediction for the C1q domain was performed using the I-TASSER server (Zhang, 2008). The results of the mapping of the read coverages onto the predicted protein structure were visualized using PyMOL version 1.2r1 (Delano, 2002).

## Results and discussion

### Identification of MVIs

**Figure 1:**
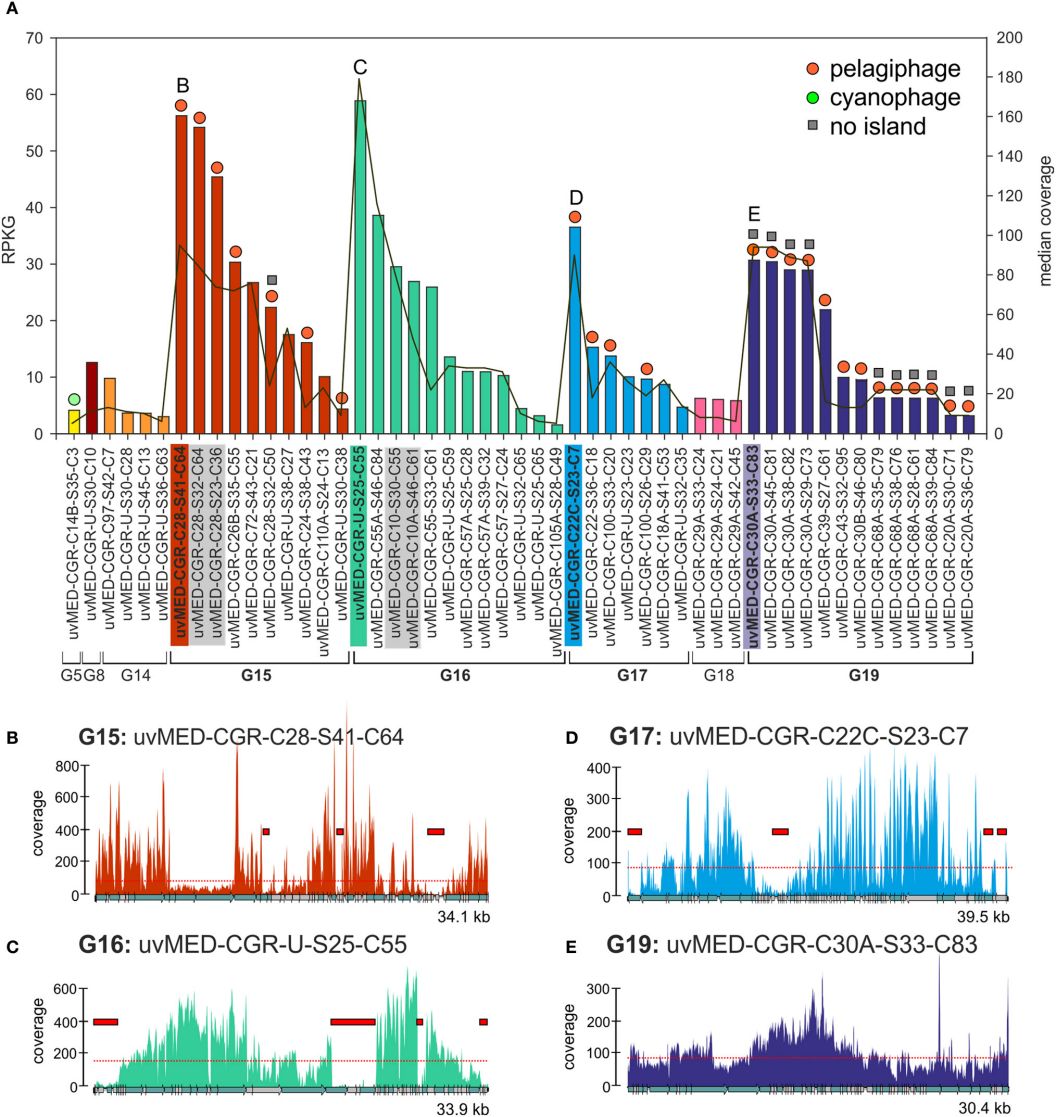
**Fragment recruitment of highly abundant CGRs. (A)** Recruitment levels of all selected CGRs in this study. Groups of CGRs are indicated on the X-axis and CGRs for each group are shown in the same color. CGRs belonging to the same cluster are highlighted in gray. Vertical bars indicate RPKG values (left Y-axis, reads recruited per Kb of sequence per Gb of metagenome). Median coverage of each CGR is shown as a red line (right Y-axis). Host prediction for each CGR, wherever available, is indicated by a colored circle on top of each bar (orange: pelagiphage, green: cyanophage, see key top right). Absence of MVIs in a CGR as deduced from recruitment analysis is indicated by a gray square on top of the bars. All CGR names have been abbreviated e.g., uvMED-CGR-C97-S42-C7 is shown instead of the complete name uvMED-CGR-C97-MedDCM-OCT-S42-C7 **(B–E)** Recruitment plots showing coverage of selected CGRs in the metavirome. These CGRs are also labeled and highlighted in the same color in the top panel. The horizontal red dotted line indicates the median base coverage and MVIs are shown as red rectangles in each plot.

We have focussed our analyses on 224 abundant contigs (RPKG >1) of which 51 were CGRs and 173 were CGFs. The results of the CGR recruitment analysis against the metavirome are summarized in Figure 1. These phage genomes and the metavirome originate from the same location, but 4 years apart. All but one of the predicted hosts for these abundant phages were Alphaproteobacteria, e.g., *Ca*. Pelagibacter (SAR11) and *Ca*. Puniceispirillum (SAR116) (Mizuno et al., 2013). This was not unexpected, as these cellular taxa are among the most abundant in the marine habitat (Delong et al., 2006; Ghai et al., 2010; Oh et al., 2010). However, there are also very abundant phage genomes for which the host is as yet unidentified (for example, those belonging to group G16) (Figure 1A, Data S1). We used a very high recruitment threshold (98% identity over at least 50 bp), designed to recruit only reads that belong to the same phage clone (accounting for an error rate of ca. 1%). It is a well proven fact that MDA, used to amplify the DNA of the metavirome, introduces biases. However, we believe the under-recruiting regions (by the large difference in coverage required) actually reflect reduced frequencies of the genes within the population.

We identified a total of 165 contigs (40 of them CGRs) out of the 224 that contained at least one MVI (See Methods, Data S1, S2). Among the CGRs where MVIs were identified, the size and recruitment levels of each MVI was variable, ranging from very small, barely covering a protein domain in a gene (close to our established lower island size limit of 500 bp) to up to 4.6 kb, covering sometimes three or four genes (Figure 1, Data S1). The fraction of the genome represented by MVIs was on average ca. 13%, (ranging from 1.53 to 29%). Figure 1 shows the coverage plots of the most recruiting individual CGRs from groups G15, G16, G17, and G19. The CGRs belonging to G15 and G17 (likely infecting microbes from the “Pelagibacterales” clade) have a similar coverage profile, with the islands representing around 10% of the phage genomes and a maximum island length of 1.5 kb (Figures 1B,D). A very different pattern with much more uneven recruitment was found for the G16 CGR representative with islands covering around 20% of the phage genome. Besides, the 3.7 kb largest island in this genome had almost no coverage (Figure 1C), suggesting that the whole fragment is absent in the metavirome.

This presence of under-recruiting islands in phage genomes is reminiscent to what was discovered years ago for cellular genomes (both bacteria and archaea) in which similar regions were found even when the metagenomes were from the same habitat from which the strains providing the genomes were isolated (Cuadros-Orellana et al., 2007). There are two possible explanations for this remarkable phenomenon. One would be that during the time span between the isolation of the strain and the sample taken to generate the metagenome the microbe has changed and most of the clones present in the metagenome are different in the under-recruiting region, in effect a Red-Queen (R-Q) arms race between phage and host accounting for the variability (Stern and Sorek, 2011). The other alternative is that prokaryotic species live in populations that are heterogeneous and diverse, containing several clonal lineages that share stretches of their genomes (more or less coincidental with their core genomes), and other regions that vary from one clone to another, and that correspond to the flexible genome. Moreover, there is evidence indicating that clones survive without significant changes for periods of decades in natural environments (López-Pérez et al., 2013). In addition, although there are fewer available examples to examine, even when the strain genome and the metagenome are from the same sample the under-recruiting islands are still detectable (Gonzaga et al., 2012). Some of us proposed that to maintain this diversity of coexisting clonal lineages, a kill-the-winner dynamics has to occur, involving phages that equalize the prokaryotic populations, preventing any clone from sweeping the others out of the population (C-D) (Rodriguez-Valera et al., 2009). To carry out this role, phage population would have to be also polyclonal with multiple concurrent lineages. This would also explain the situation depicted by Figure 1.

We also found 11 CGRs (ca. 20% of the abundant ones) in which not a single island could be identified (Figures 1A,E and Data S2). All except one of the MVI free CGRs belong to G19, a group that was described as putative pelagiphage based on similarity to a prophage detected in the genome of the alpha proteobacterium HIMB114, a member of the proposed order “Pelagibacterales” obtained from the coastal tropical North Pacific (Grote et al., 2012). Along similar lines, we recently found a nearly identical genome fragment in the Mediterranean (ca. 40 kb) with >97% identity to the cyanophage isolate S-CAM1 from the Pacific Ocean (South California Coast) which indicates that some phage clones are very stable and durable (Mizuno et al., 2013). These phage clones appear to be conserved and present in significant amounts in two independent samples separated by more than 4 years. However, even in this case, the phage population was still composed of different clones as illustrated by the sequence differences detected among individual genomes and by the uneven recruitment. It is possible that these are generalistic phages that recognize multiple host clonal lineages (Flores et al., 2011).

### Genomic islands in phages and MVIs

**Figure 2:**
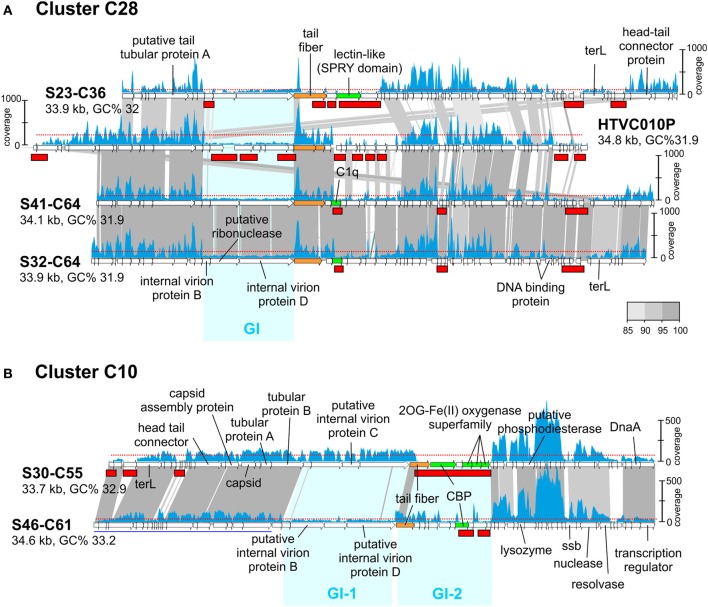
**Genomic comparisons of phages from cluster C28 and C10 and their fragment recruitment in the DCM metavirome. (A)** Three CGRs from C28 predicted to be pelagiphages and the genome of isolated *Ca*. Pelagibacter ubique phage HTVC010P are compared. **(B)** Two CGRs from C10 are compared. Some genes of S46-C61 are shown underlined in blue indicating that they have been moved to improve comparison. The read coverage in the metavirome is plotted on top of each genome (a scale is shown either to the left or right of the genome). The horizontal red dotted line indicates the median base coverage and MVIs are shown as red rectangles in each plot. Similar regions between adjacent genomes are colored according to their nucleotide identity (color scale is shown to the right). Phage genome identifiers are abbreviated e.g., S23-C36 for uvMED-CGR-MedDCM-OCT-S23-C36. Genomic Islands (GI) are highlighted in cyan.

Like in prokaryotic genomes, phage genomes are composed of more conserved regions that could be called “core” and regions that vary (“flexible”) among otherwise closely related genomes (Angly et al., 2009). In a previous work (Mizuno et al., 2013), comparing the same sets of CGRs we detected that these genomes display a somewhat uneven similarity in which nearly identical genomic regions are juxtaposed with regions with no sequence similarity whatsoever, that is, with essentially completely different genes. Most phage genomes, even from the same location and sample, appear to be extremely plastic with large flexible genomic islands that vary in sequence from one clone to another within a framework of relatively similar genomes (Mizuno et al., 2013). This is similar to what happens in prokaryotic genomes where the flexible genome (variable from one strain to another) tends to concentrate in genomic islands. In prokaryotic cells, the flexible genomic islands tend to be also under-recruiting in metagenomes. In other words, they are also MGIs. This seems to be the case with phages and genomic islands (GIs), as shown in Figure 2A where we have compared the pelagiphage genome HTVC010P retrieved from Bermuda Hydrostation S (Sargasso Sea) (Zhao et al., 2013) with a cluster (C28) of three related MedDCM pelagiphage genomes and their recruitments from the metavirome. The regions that appear more specific to a particular phage lineage, reminiscent of flexible genomic islands of prokaryotes, also recruited at lower levels than the more conserved tracts of these genomes. Although this might be considered an obvious expectation, it reflects a persistence of the situation that is remarkable. It shows that the conserved regions among these closely related lineages transcend large geographic distances (Bermuda to the Mediterranean) and significant time gaps (4 years).

Another interesting example can be seen in Figure 2B, where both phage genomes in cluster C10 share around 60% of the genome at a high sequence identity (more than 90%). However, there are two regions identified in Figure 2B as GI-1 and GI-2 with little or no sequence similarity. GI-1 in S30-C55 recruits similarly to the rest of the genome, but the corresponding region in S46-C61 does not. In the same manner, the S46-C61 version of GI-2 recruits (albeit unevenly) while the corresponding region in S30-C55 does not recruit at all. This illustrates that some phage genomic regions are extremely labile. In this case GI-2 that codes (among other proteins) for the tail fiber and the carbohydrate binding protein (CBP) of the S30-C55 phage genome appears to have disappeared from the population; either as a result of the lineage that contained this island becoming extinct during the 4 years elapsed between the retrieval of the genomes and the metavirome, or it is present at extremely low abundance and is not detected at this level of sequencing. To a lesser degree, the same happens with GI-1 of S46-C61.

In conclusion, the results indicate clearly how the most variable regions when comparing closely related genomes are also the ones under-recruiting, similarly to what happens with cellular genomes. The patterns found for the phage GIs are very similar to those found to affect the O-chain of the LPS in Gram-negative marine bacteria (Rodriguez-Valera et al., 2009; Hooton et al., 2011) that is supposed to be a major phage receptor (Avrani et al., 2011; Hooton et al., 2011). However, a major difference is that while the gene clusters in the cellular genomes are extremely divergent, even at the level of gene content and size of the clusters, in the phages, the size and number of genes in the MVIs were quite well preserved. That is, while the cellular genomes MGIs contain different gene clusters, MVIs seemingly contain the same genes but with very different sequence.

### Genes found in MVIs

We found a total of 390 predicted genes in CGRs MVIs. The vast majority (252) could not be assigned any function (hypothetical proteins) (Table 1, Data S1). The most frequent identifiable genes in MVIs were annotated as containing domains involved in molecular recognition. Specifically, these were the putative carbohydrate binding domain containing protein (14), the tail fiber protein (12), C1q globular head like domain containing protein (10), and lectin like domain protein (9). The tail fiber protein is known to be involved in host recognition (Yu and Mizushima, 1982; Bartual et al., 2010; Garcia-Doval and Van Raaij, 2012) and many studies have reported the high variability of this protein between nearly identical phages (Angly et al., 2009; Labrie et al., 2013).

**Table 1:** **Annotation and frequency of the most frequent genes found in MVIs**.

**Annotation**	**Frequency**	**Inferred function**
Putative carbohydrate binding domain containing protein	14	Host recognition
Tail fiber protein	12	Host recognition
Terminase small subunit	10	DNA packaging
C1q like domain containing protein	10	Host recognition
Lectin-like domain containing protein	9	Host recognition
Fe(II)-dependent oxygenase superfamily protein	8	Host recognition
Terminase large subunit	6	DNA packaging
Phage-like element PbsX protein XkdW	4	unknown
Putative tail fiber assembly protein	4	Phage structure
Fibrinogen-like coiled coil protein	3	Host recognition
Phage tail fiber adhesin Gp38	3	Host recognition
Tape measure protein	3	Phage structure
DNA binding domain	3	DNA binding
Putative internal virion protein	3	Host cell penetration
Hypothetical protein	252	unknown
Others	46	other
Total	390	

In phages there are two stages in identifying the cells to be infected (Rakhuba et al., 2010). The initial step is reversible and is carried out by the tail fiber. The second leads to irreversible binding to the host cell and is carried out by the receptor binding protein (RBP) that is highly variable depending on the phage structure and classification. The genes coding for both steps are typically located close to each other in phage genomes, what facilitates the identification of this recognition module (Miller et al., 2003; Leiman et al., 2004). From the most frequent under-recruiting genes, we identified three as the most likely candidates to be the RBP due their well-known capability to interact with other proteins or other substrates. These are proteins containing C1q, carbohydrate binding or a lectin like domains. In addition, fibrinogen-like coiled coil and filamentous hemagglutinin-like proteins were also identified as such, considering the structural similarity with the viral receptor-recognition proteins of Influenza virus (Skehel and Wiley, 2000). In summary, out of 138 identifiable genes in MVIs, 59 (Table 1) are clearly related to the host recognition module and therefore could reflect the specialization in infecting different clones of host, and they are enriched in MVIs as the C-D model would predict.

Another conspicuous component of under-recruiting islands (Table 1) were the small and large subunits of the terminase (these proteins are involved in packaging of the phage genome into the capsid) and other genes whose involvement in host-phage interaction appears more remote (if any). For example, three internal virion proteins, a protein involved in host cell wall penetration appeared also in MVIs. The reasons for the under-recruitment of these genes are more obscure. The repeated identification of these genes within MVIs in different phage genomes makes it unlikely that their under-recruitment is artifactual and probably reflects our incomplete understanding of phage biology.

### Metagenomic recruitment within the host recognition module genes

A way to glimpse at the biological meaning of the differences in recruitment throughout phage genomes is to dissect the recruitment of different regions, with well-established roles, within a single gene. Regions that under-recruit within a single gene are either very variable among the different lineages in the population (C-D) or within the same lineage at different points in time (R-Q). Since the pelagiphages are the highest recruiting phages in the metavirome (Figure 1), we have chosen them for more detailed recruitment analyses. Both cluster C28 and C24 contain CGRs that are predicted to prey on *Ca*. Pelagibacter due their relatedness to the pelagiphage HTVC010P (Mizuno et al., 2013). We have selected those genes in these genomes (clusters C28 and C24) that could be identified as involved in both host recognition steps of the phage life cycle (see above). Specifically, these are the tail fiber (the first step) and C1q domain containing genes (RBPs, the second step). We examined the recruitment patterns of the different functional protein domains identifiable within these genes.

**Figure 3:**
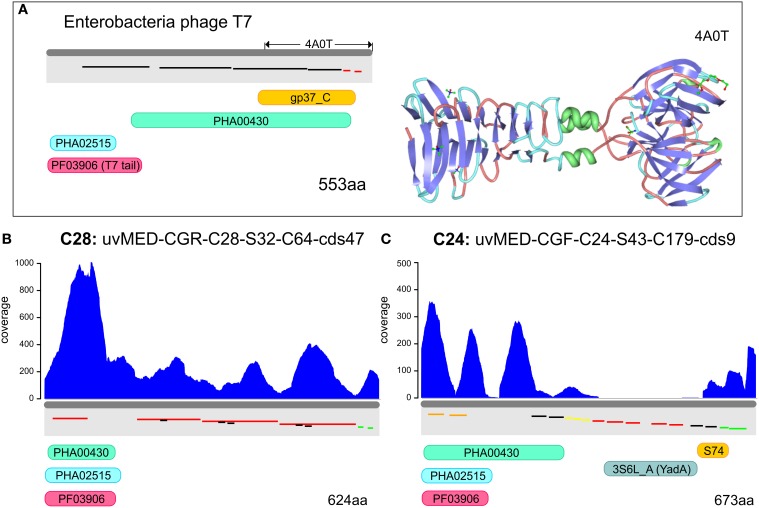
**Metagenomic read recruitment and protein domain structures of putative phage tail fiber proteins. (A)** The protein domain structure of Enterobacteria phage T7 tail fiber protein and the solved crystal structure of the C-terminus (PDB ID: 4A0T) is shown for reference. Repeats are shown staggered within the gray rectangles and colored according to repeat type. **(B,C)** Read coverage and protein domain structure in two tail fiber proteins. Read coverage is plotted in blue above each protein. Protein domains are shown below (PF03906, PFAM domain of phage tail fiber N-terminus; gp37_C, PFAM domain of phage gp37 C-terminus; S74, peptidase domain; 3S6L_A, YadA-like protein; PHA02515, hypothetical phage protein; and PHA00430, putative phage tail fiber).

In order to identify the most variable region within the tail fiber protein, we have used the tail fiber of the phage T7 of *Escherichia coli* as a reference (Garcia-Doval and Van Raaij, 2012). This protein (553 aa) shown in the top panel of Figure 3 has a very characteristic domain structure, including a well conserved N-terminal domain (PF03906), followed by several repeats. The C-terminal domain (gp37_C) is known to be involved in host interaction in the T7 bacteriophage. The crystal structure of the C-terminal domain has been described (Figure 3A) and a number of possible binding receptors suggested, leaving little doubt that this region is involved in host recognition by T7 (Garcia-Doval and Van Raaij, 2012). The tail fiber protein sequences from the selected phage genomes of cluster C28 and C24 preserved the same domain structure (T7-like) at the N terminus (PF03096). This was followed by a repeat-pattern specific to each tail fiber protein but without any sequence similarity. A similar repetitive stretch is also seen in the T7 tail fiber protein, suggesting a similar secondary structure (Figure 3). No similarity was discernible in the C-terminal regions. One of the tail fiber proteins has a C-terminal YadA domain (Figure 3C) that is a typical adhesion like domain (Casutt-Meyer et al., 2010; Edwards et al., 2010). These observations taken together suggested that the C-terminal region of the tail fiber of these pelagiphages are much more variable than the N terminus and are likely candidates to be involved in host recognition, as shown for the Enterobacteria phage T7. Along these lines, the metagenomic coverage plot of both tail fibers shows a marked decrease for the C terminal region, particularly in the case of the representative of cluster C24, the region where the YadA domain is located.

**Figure 4:**
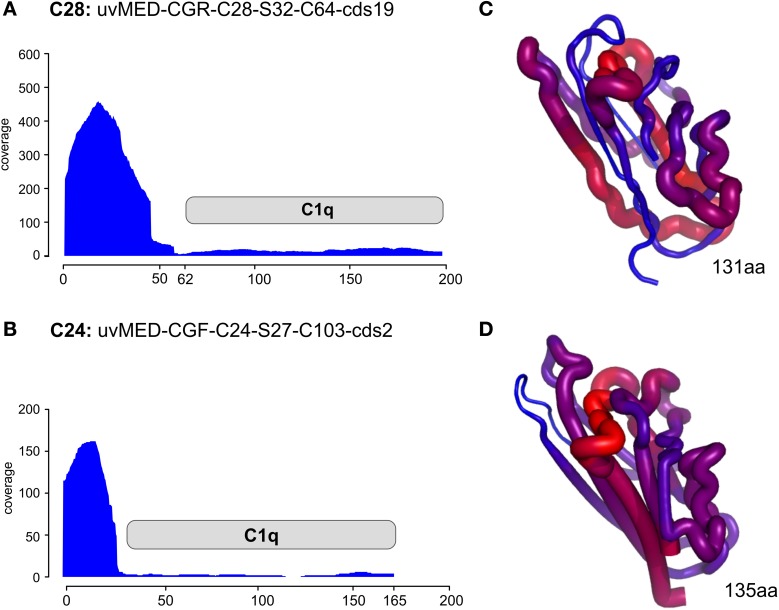
**Metagenomic recruitment by the C1q domain containing proteins**. Two different C1q domain containing proteins are shown. **(A,B)** Read coverage in the DCM metavirome is shown on the vertical axis on the left. The location of the C1q domain is marked by a gray rectangle. **(C,D)** the predicted corresponding structure of only the C1q domain in each protein is shown. The thickness of the backbone chain and the color indicate the coverage levels (thick/red- high coverage, thin/blue- low coverage).

Even more compelling is the example of the putative RBP in these phages (e.g., the C1q domain containing gene) found next or close to the tail fiber. The C1q protein is a well-known target recognition molecule of the classical vertebrate serum complement pathway (Kishore et al., 2004; Ghai et al., 2007). Figure 4 shows the recruitment levels of two C1q domain containing proteins from pelagiphages of both C28 and C24. These domains always under-recruited conspicuously, fitting well with the expectation of a highly variable protein that is likely involved in host recognition. In both the examples shown, the N-terminal end recruits far more than the C1q domain. We created homology-based protein structure models for both these C1q domains and mapped the recruitment from the metagenome to the protein structures. Both these C1q domains are only 38% similar to each other, and display different regions of variability (blue in the right-hand panels of Figure 4). It appears that these regions, that under-recruit in comparison to the rest of the sequence may be the sites of hyper-variability in these proteins. These results indicate several levels of variability even among concurrent phage lineages, ranging from large MVIs specific to each clonal lineage, and likely also existence of numerous sub-lineages even amongst each, as can be observed by the variation in the structural scaffold of C1q. Phage genomes are naturally constrained by a fixed size and show much more differences in specific sequence regions within genes rather than different genes altogether. This could just reflect the obvious constraints of genome size affecting phages and viruses in general.

### Conclusions, red queen or constant-diversity

The identification of genomic islands in these genomes (metaviromic and flexible) and their differential persistence across time and space leads us to speculate on the nature of the dynamics in these populations. While R-Q predicts that the molecular modules involved in host-phage interaction will vary very rapidly with evolutionary time, C-D posits that many versions of such modules are present simultaneously, at any given time, within the population. The differences between these two models are subtle and linked to time. In our datasets, the time span between genome and metavirome retrieval is only 4 years, which, evolutionarily speaking, is a short period in this relatively stable habitat. There is also evidence that some host-recognition modules (e.g., tail fiber protein) are preserved over large geographic distances (S31-C64 from MedDCM and HTVC010P from Bermuda, Figure 2). Moreover, within this short time span, it was possible to retrieve phage genomes that do not present any MVIs (i.e., persistent lineages). However, we have also found cases where a MVI did not recruit any reads at all (for example, GI-2 in S30-C55, Figure 2), suggesting that this particular lineage might have disappeared altogether (or is undetectable at current sequencing depth). Even so, taken together these results appear to support persistence in lieu of continuous variability, and hence favor more a C-D, than a R-Q dynamics in these populations.

It seems clear that the genomic diversity of phage populations is outstanding and yet, some genomes appear very well preserved over long distances and time spans. Overall, the data support the C-D model (Rodriguez-Valera et al., 2009) and a more recent reformulation (Rodriguez-Valera and Ussery, 2012) in which some of us proposed that prokaryotic species and their accompanying phages form consortia that have been evolving together over many millions of years. Akin to the starters used in dairy, such consortia work well as a package that have been selected for in nature and can be found with very similar structure at similar habitats worldwide. If this hypothesis is true, the consortium that develops in the deep (below 20 m) photic zone of stratified waters such as the MedDCM could be one of the most extensive on Earth and of enormous relevance to the global ecology of the planet.

## Author contributions

Francisco Rodriguez-Valera conceived the work. Carolina Megumi Mizuno and Rohit Ghai performed all analyses. The manuscript was written by Francisco Rodriguez-Valera and Rohit Ghai.

## Conflict of interest statement

The authors declare that the research was conducted in the absence of any commercial or financial relationships that could be construed as a potential conflict of interest.
