# Supplementary material for: Evidence for metaviromic islands in marine phages
Source: Front Microbiol. 2014 Feb 3;5:27. doi: 10.3389/fmicb.2014.00027 (PMC3909814; doi:10.3389/fmicb.2014.00027)
Supplement: Supplementary file 2 [file DataSheet2.PDF]

Data S2

Evidence for Metaviromic Islands in Marine Phages

Carolina Megumi Mizuno, Rohit Ghai and Francisco Rodriguez-Valera

This file contains 51 coverage plots of CGRs arranged in two sections.

(A): Coverage plots of 40 complete phage genome representatives (CGRs) against the metavirome. MVIs were detected in all these CGRs. The islands are plotted as red rectangles. Gaps in the contigs (if present) are plotted as green rectangles. A dotted horizontal line indicates the median coverage of the contig.

(B): Coverage plots of 11 complete phage genome representatives (CGRs) against the metavirome. No MVIs were detected in all these CGRs. Gaps in the contigs (if present) are plotted as green rectangles. A dotted horizontal line indicates the median coverage of the contig.

(These legends above are repeated at the beginning of each section as well)

(A): Coverage plots of 40 complete phage genome representatives (CGRs) against the metavirome. MVIs were detected in all these CGRs. The islands are plotted as red rectangles. Gaps in the contigs (if present) are plotted as green rectangles. A dotted horizontal line indicates the median coverage of the contig.

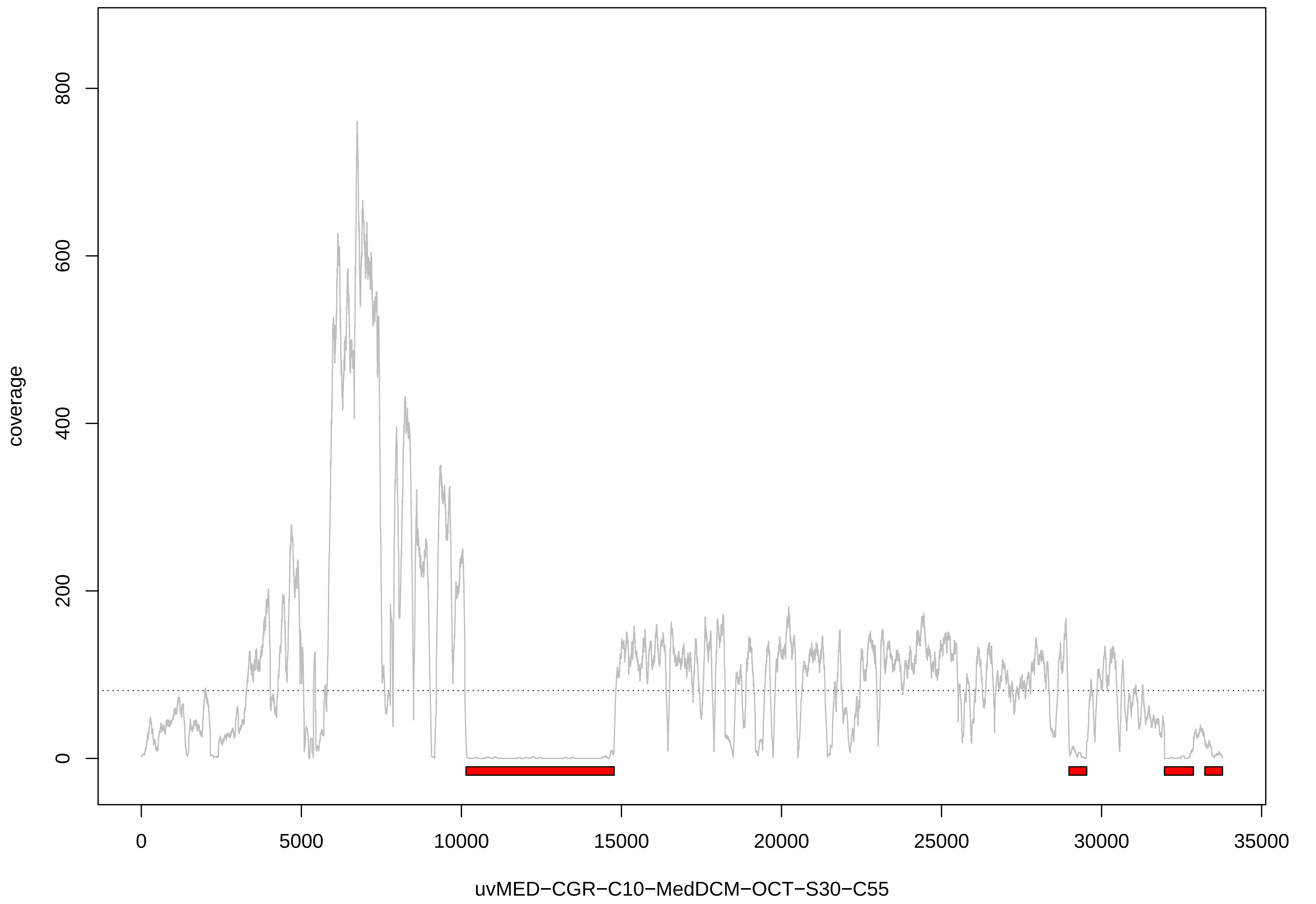
coverage
0
200
400
600
800
0
5000
10000
15000
20000
25000
30000
35000
uvMED−CGR−C10−MedDCM−OCT−S30−C55

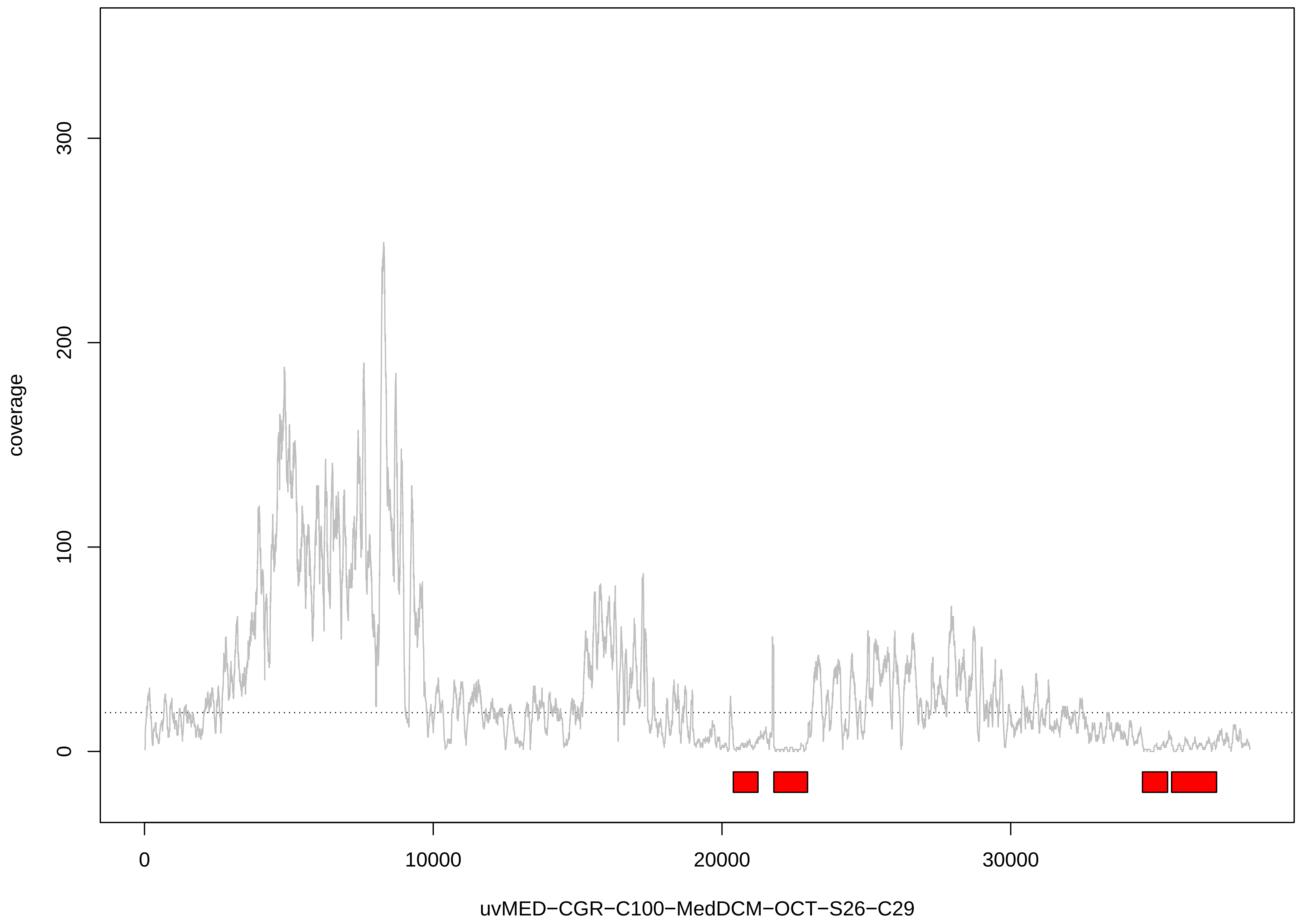

coverage
0
100
200
300
0
10000
20000
30000
uvMED−CGR−C100−MedDCM−OCT−S26−C29

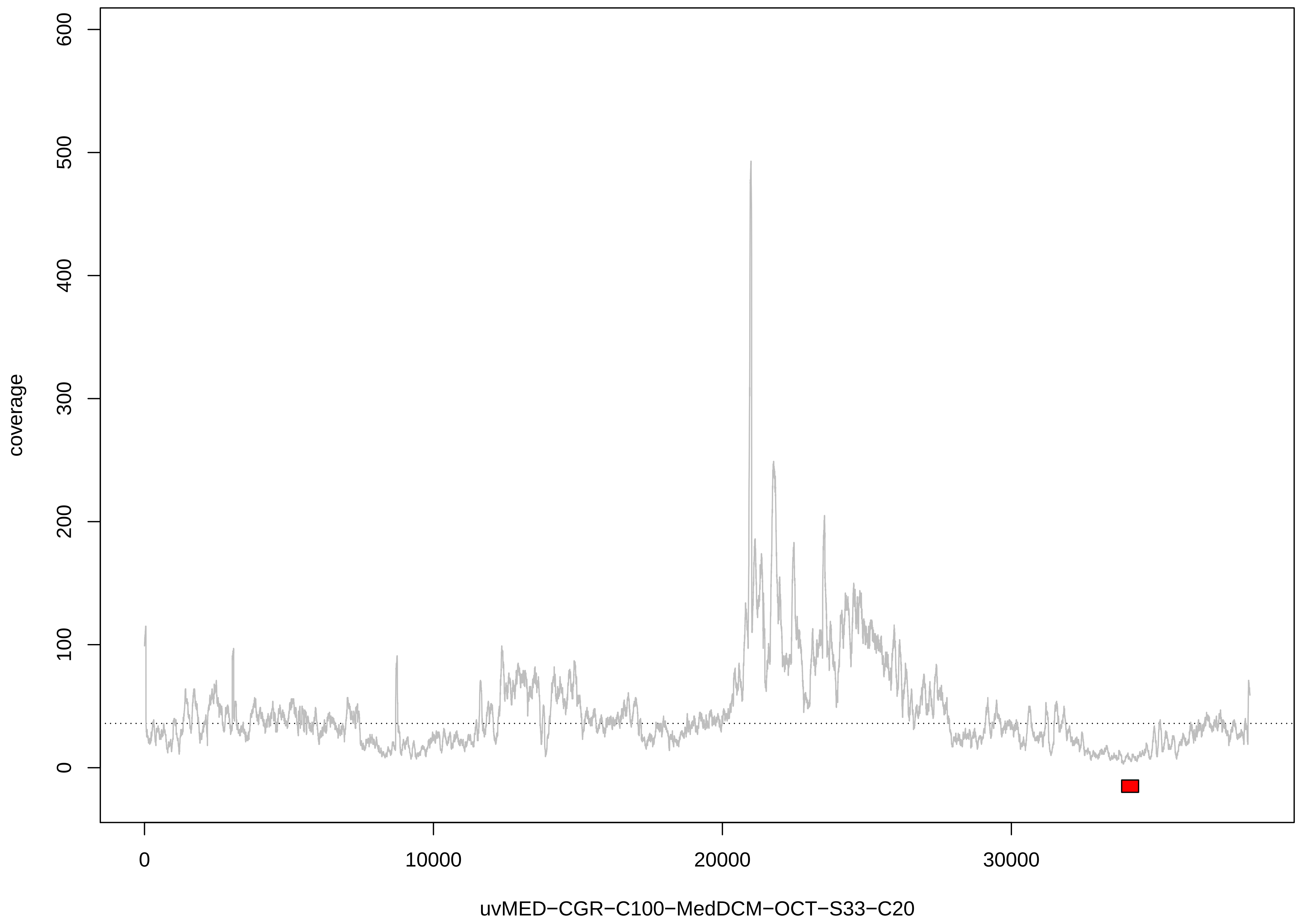

coverage
0
100
200
300
400
500
600
0
10000
20000
30000
uvMED−CGR−C100−MedDCM−OCT−S33−C20

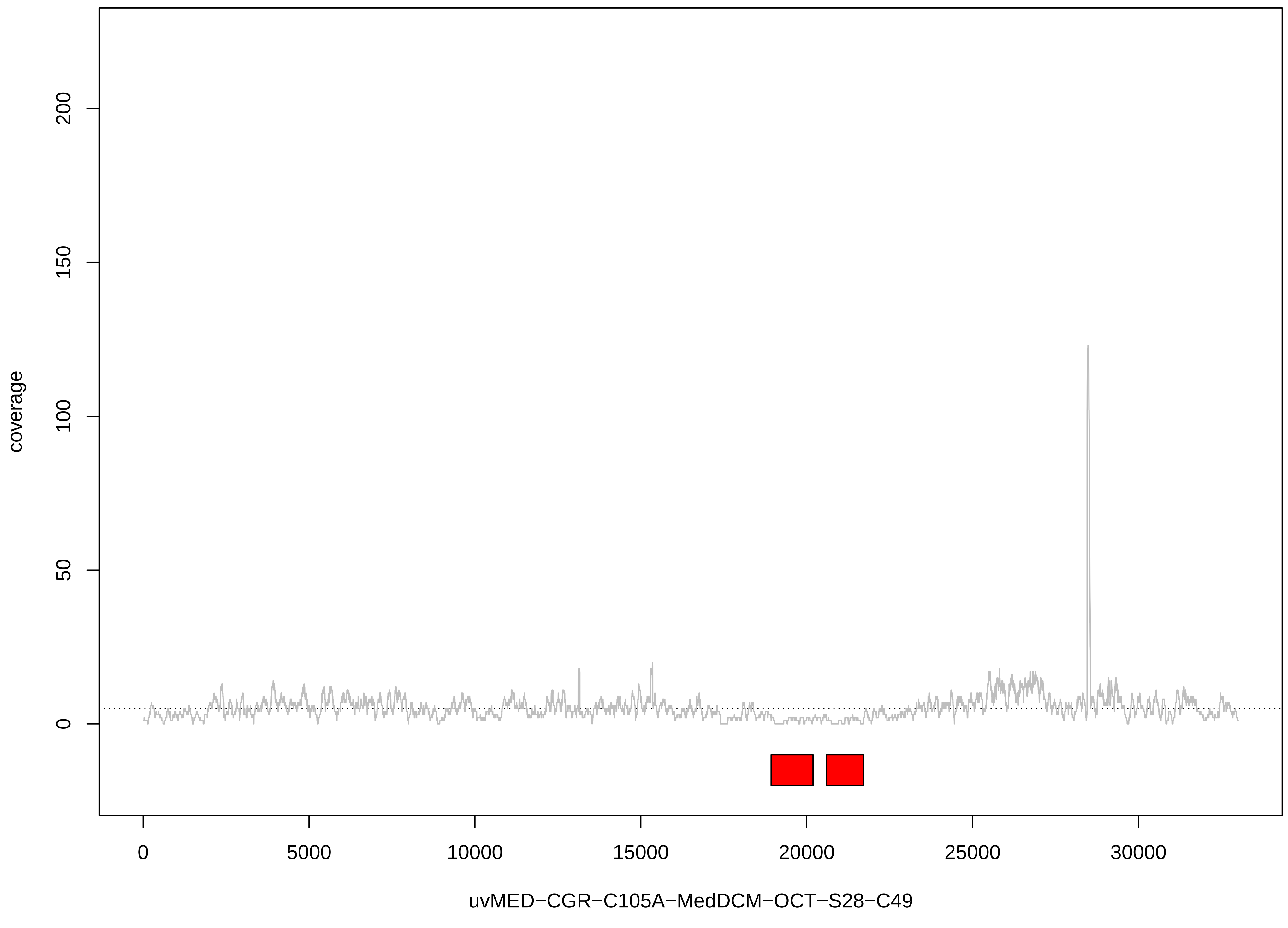

coverage
0
50
100
150
200
0
5000
10000
15000
20000
25000
30000
uvMED−CGR−C105A−MedDCM−OCT−S28−C49

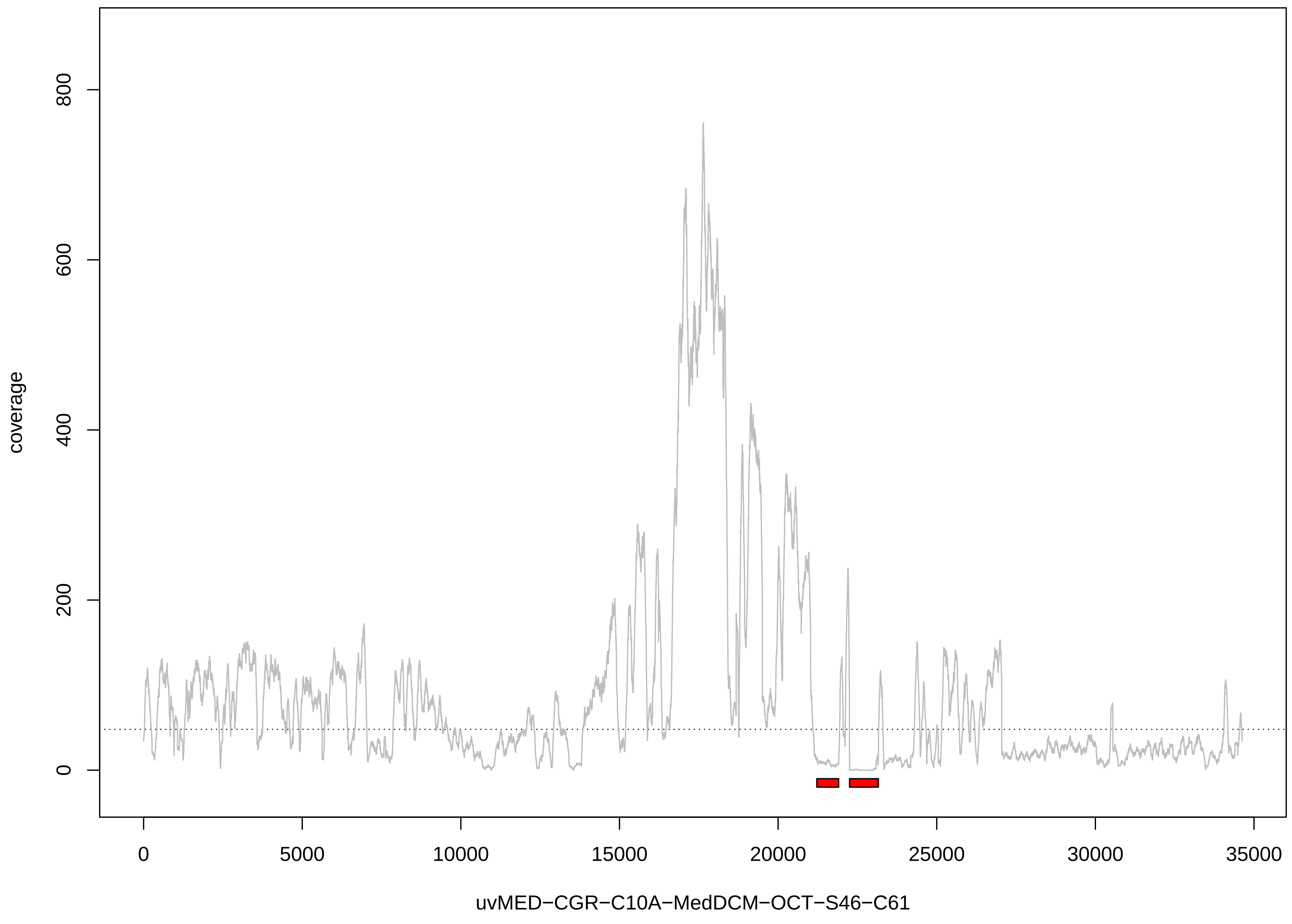

coverage
0
200
400
600
800
0
5000
10000
15000
20000
25000
30000
35000
uvMED−CGR−C10A−MedDCM−OCT−S46−C61

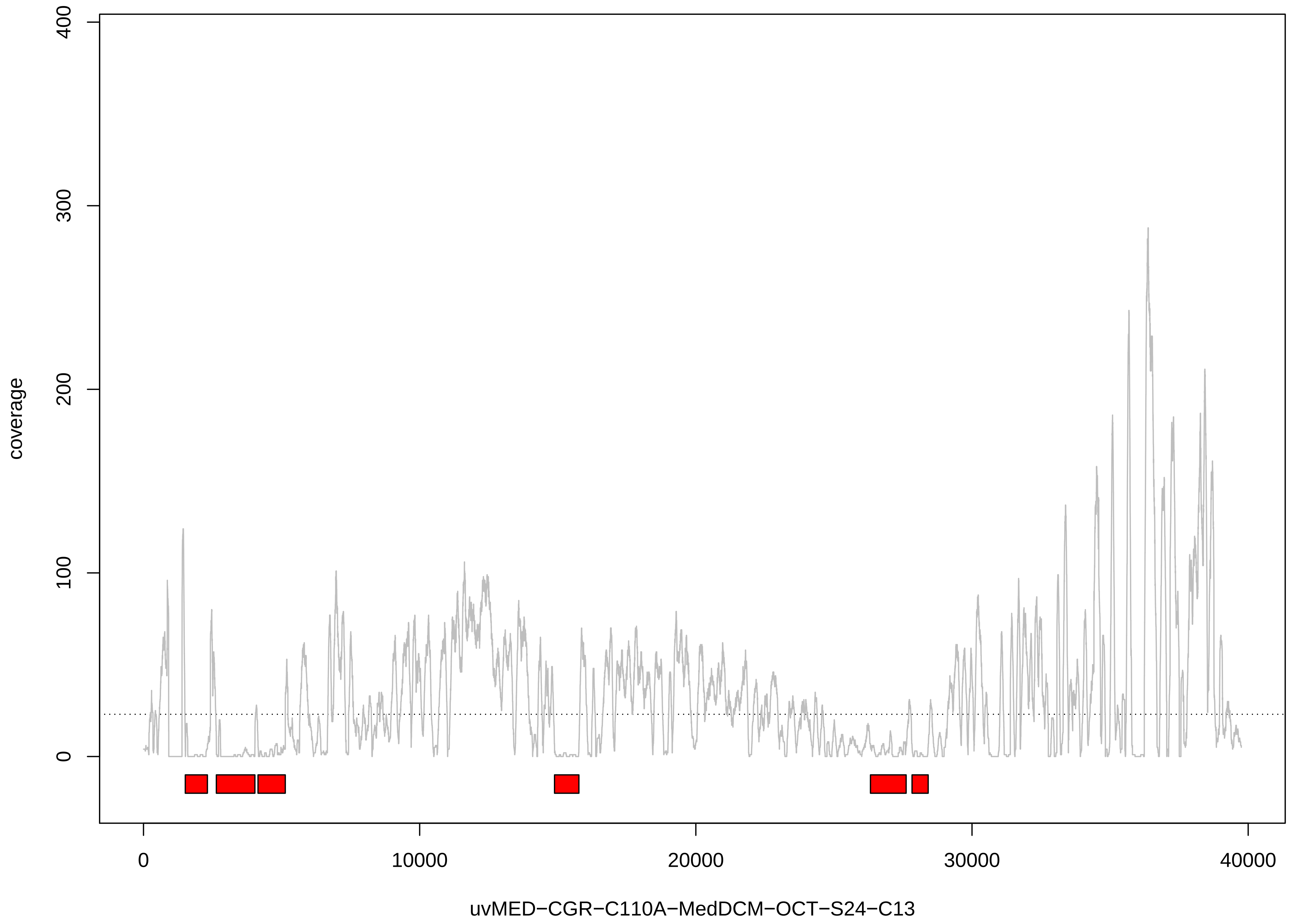

coverage
400
300
200
100
0
0
10000
20000
30000
40000
uvMED−CGR−C110A−MedDCM−OCT−S24−C13

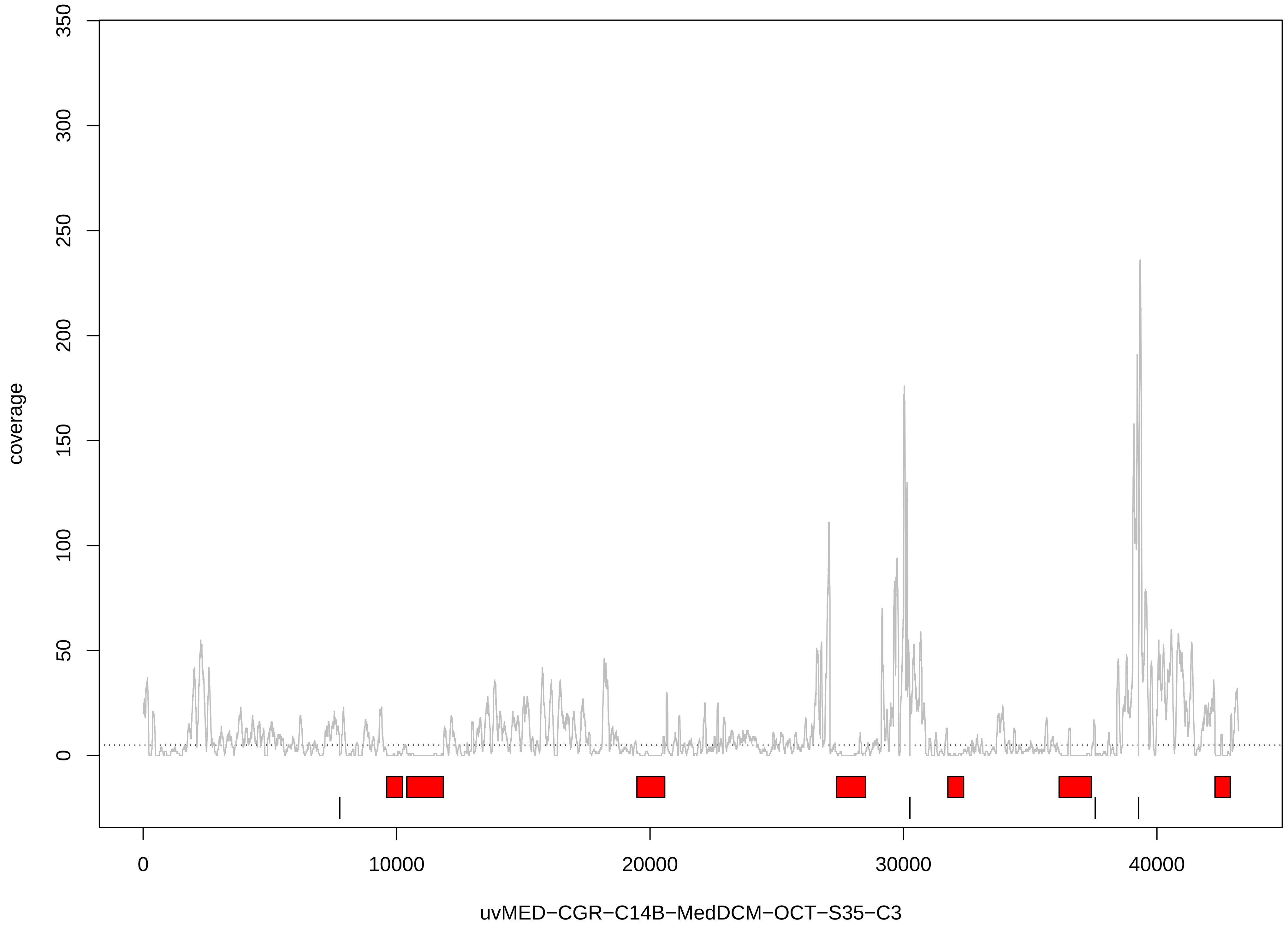

coverage
0
50
100
150
200
250
300
350
0
10000
20000
30000
40000
uvMED−CGR−C14B−MedDCM−OCT−S35−C3

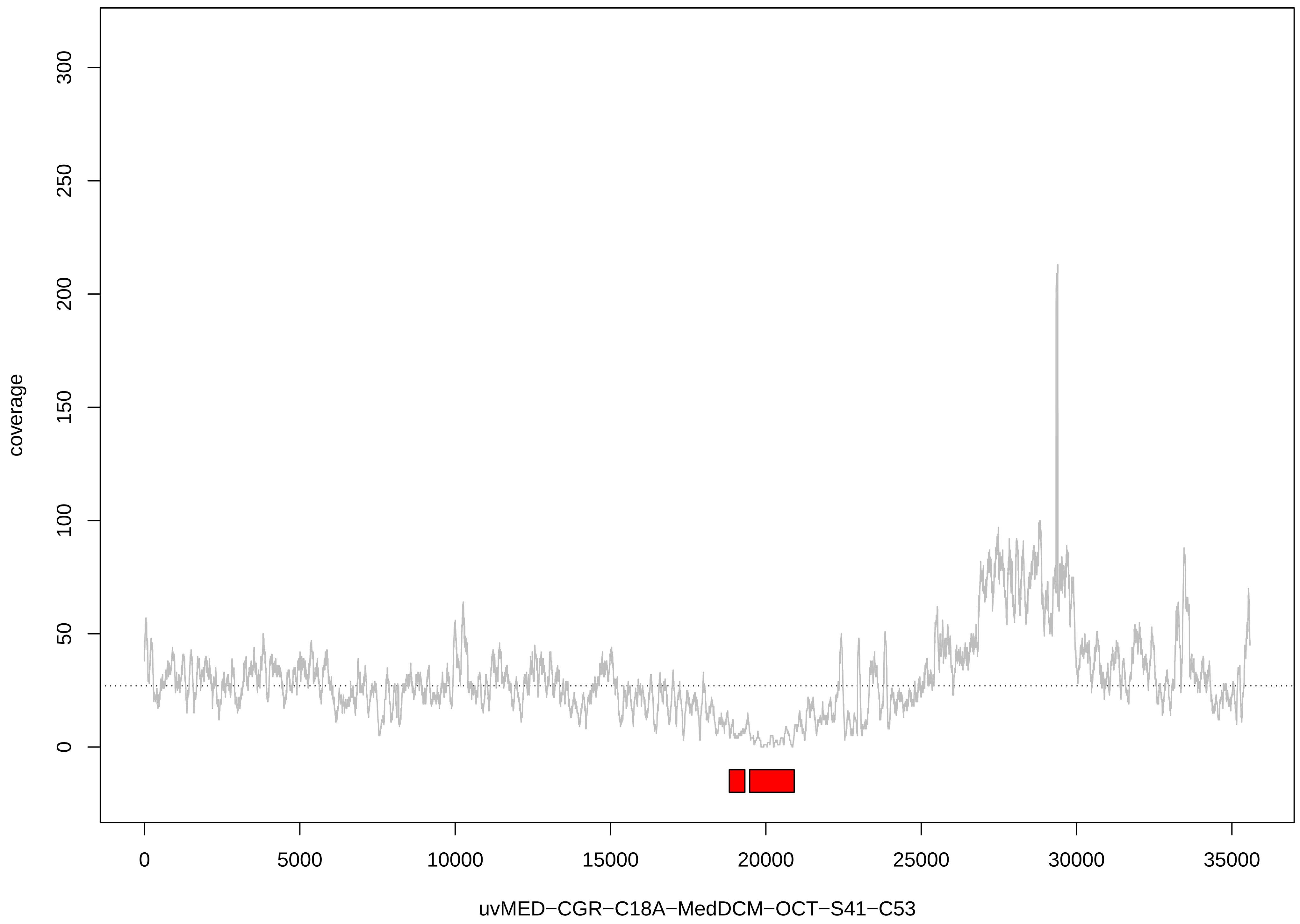
coverage
0
50
100
150
200
250
300
0
5000
10000
15000
20000
25000
30000
35000
uvMED−CGR−C18A−MedDCM−OCT−S41−C53

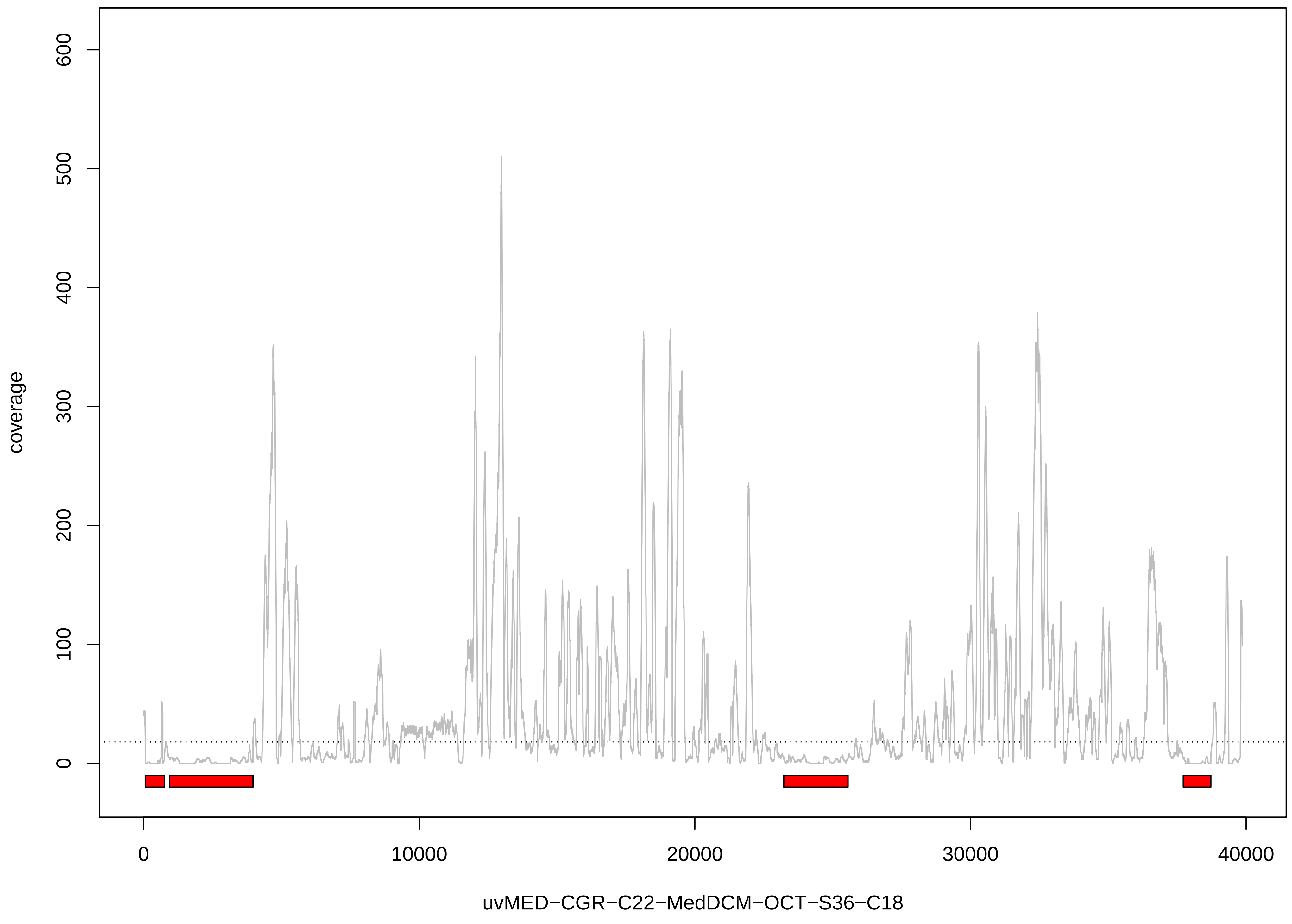
coverage
0
100
200
300
400
500
600
0
10000
20000
30000
40000
uvMED−CGR−C22−MedDCM−OCT−S36−C18

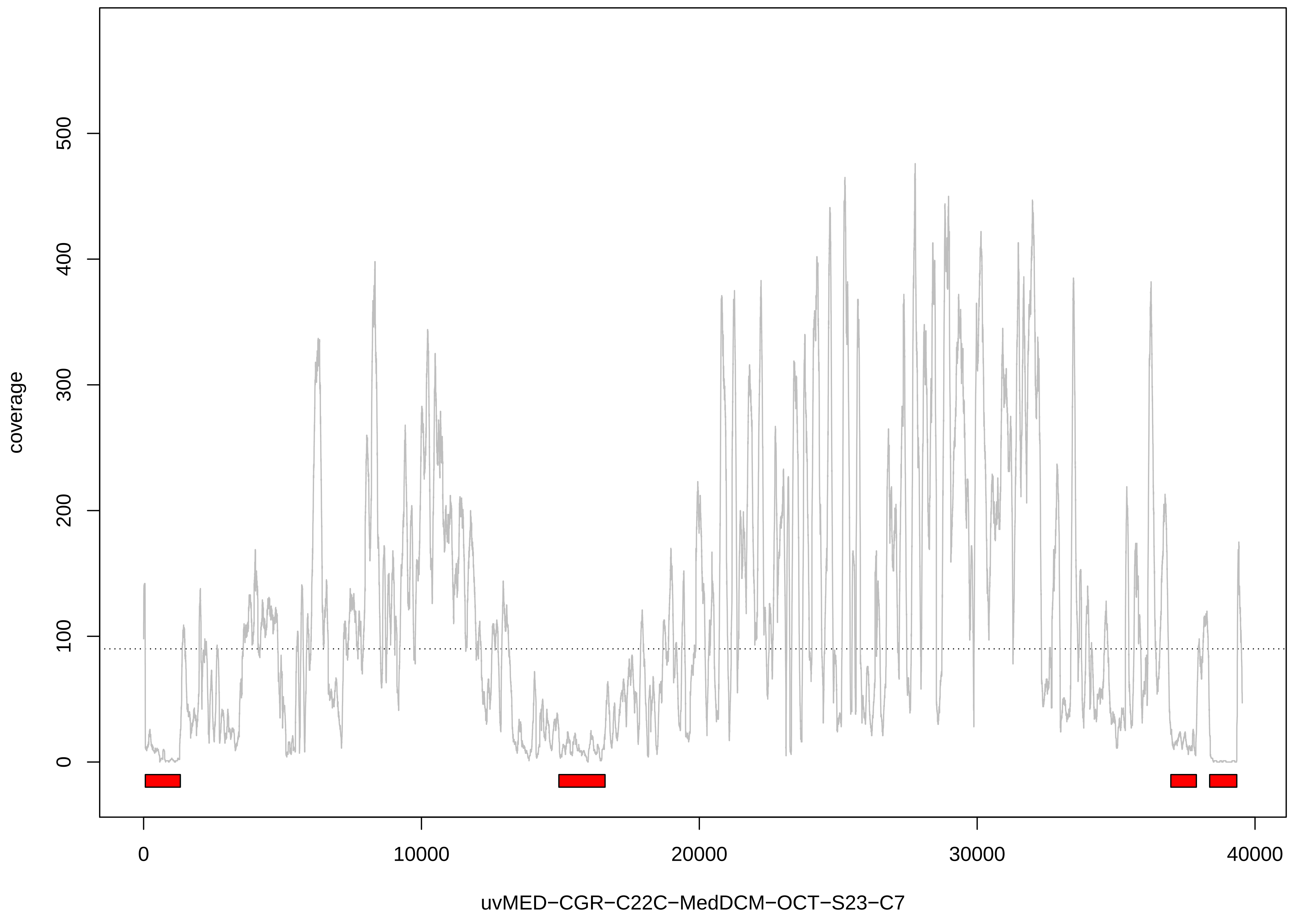
coverage
0
100
200
300
400
500
0
10000
20000
30000
40000
uvMED−CGR−C22C−MedDCM−OCT−S23−C7

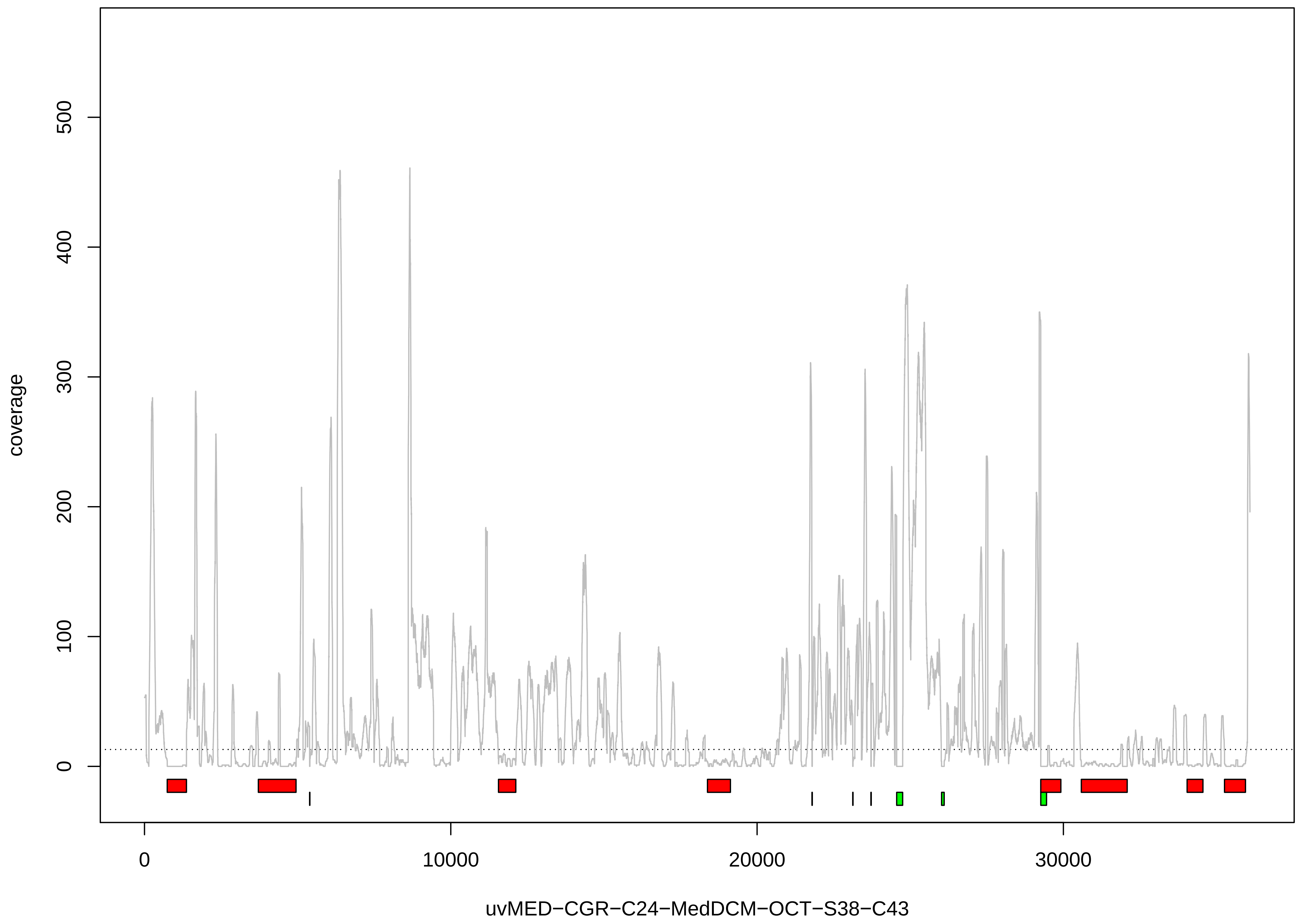
coverage
0
100
200
300
400
500
0
10000
20000
30000
uvMED−CGR−C24−MedDCM−OCT−S38−C43

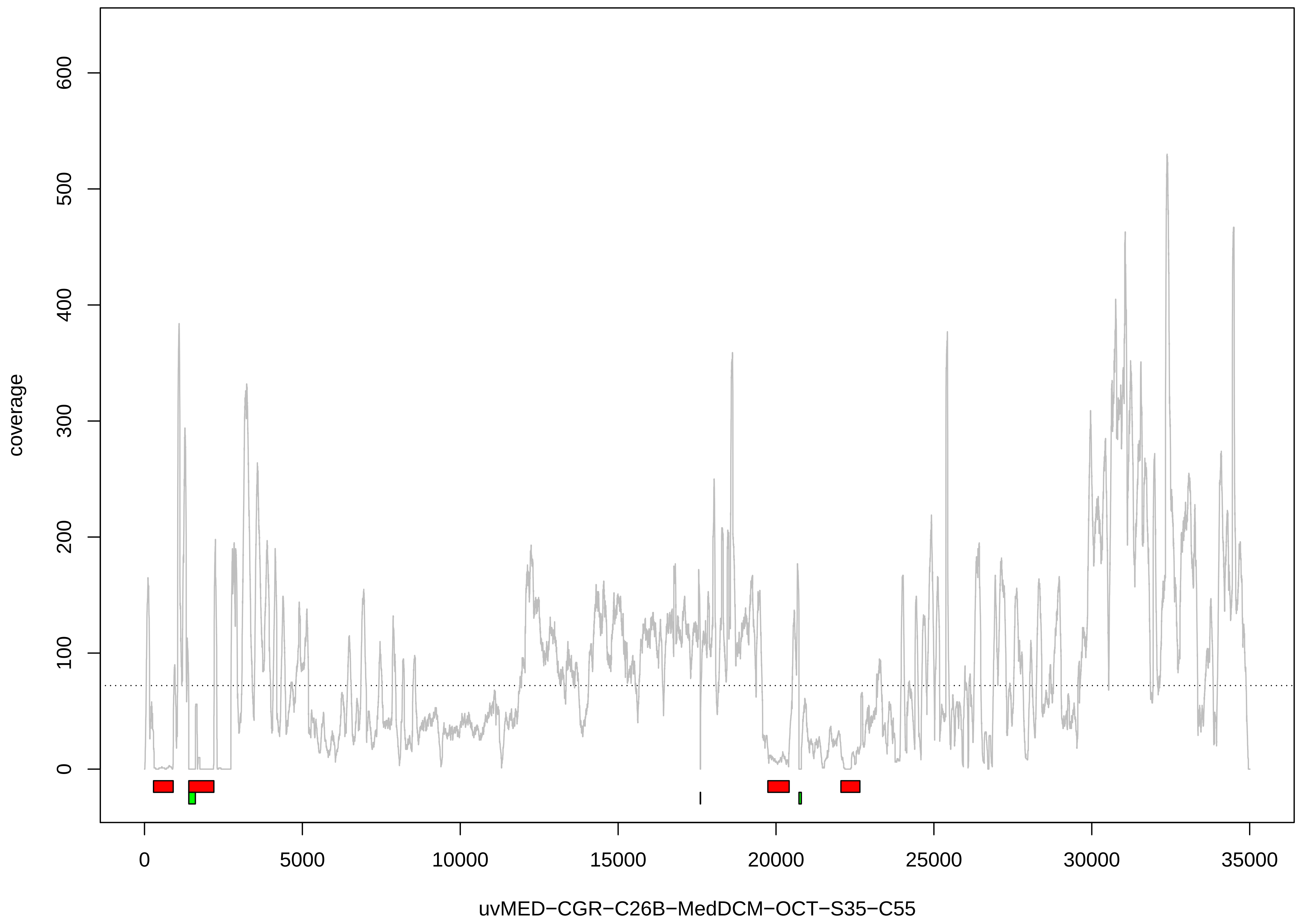

coverage
0
100
200
300
400
500
600
0
5000
10000
15000
20000
25000
30000
35000
uvMED−CGR−C26B−MedDCM−OCT−S35−C55

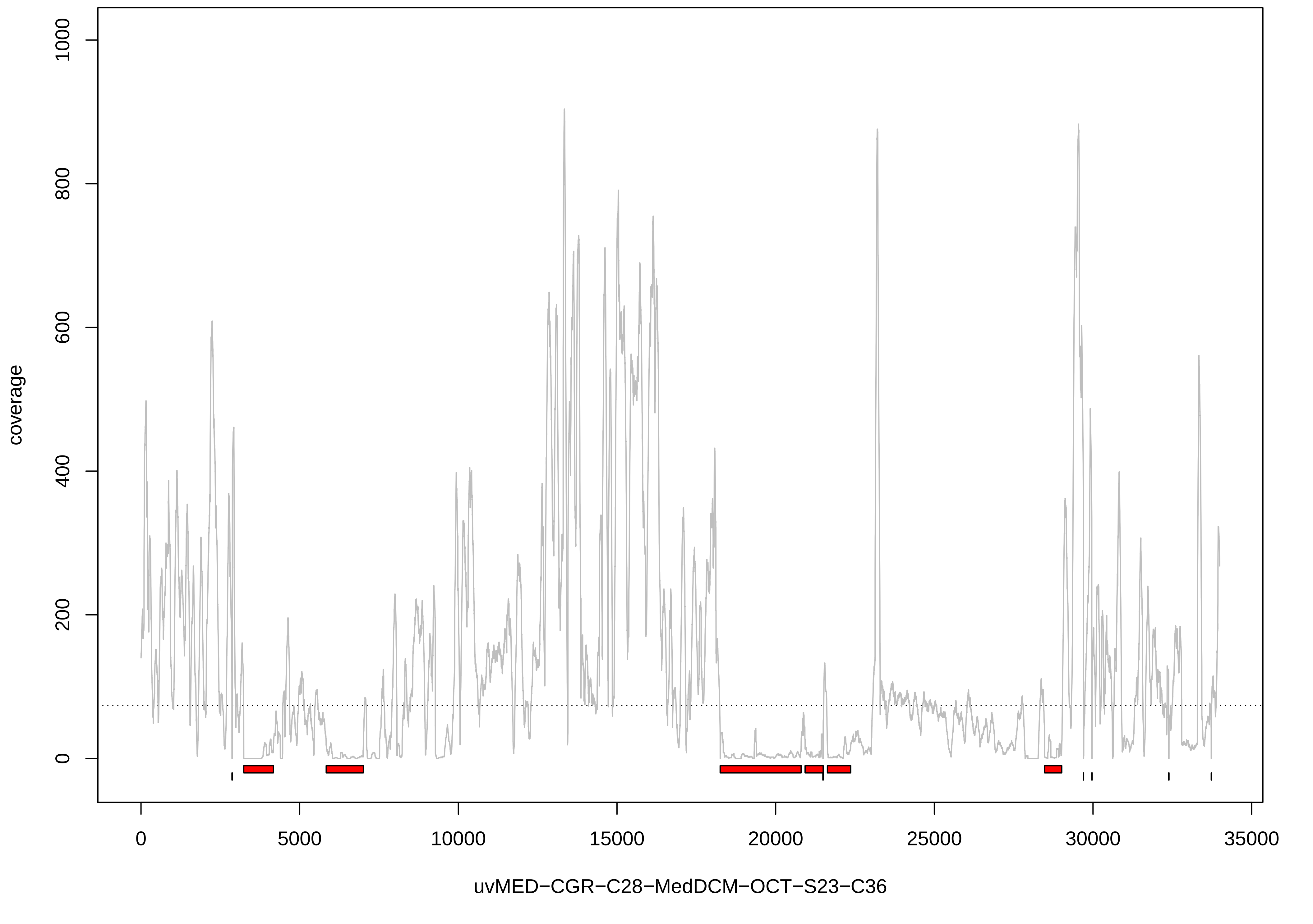

coverage
0
200
400
600
800
1000
0
5000
10000
15000
20000
25000
30000
35000
uvMED−CGR−C28−MedDCM−OCT−S23−C36

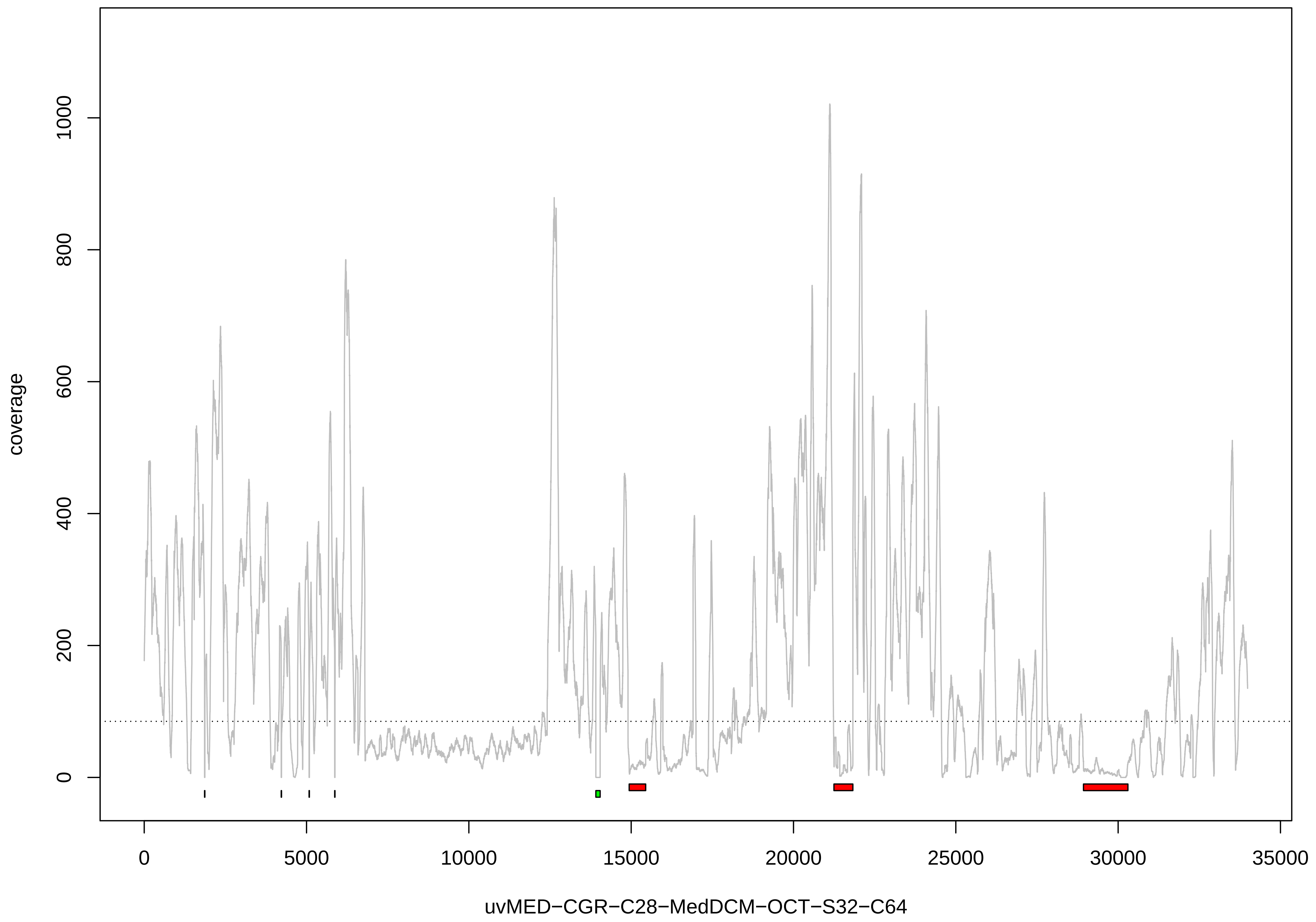

coverage
0
200
400
600
800
1000
0
5000
10000
15000
20000
25000
30000
35000
uvMED−CGR−C28−MedDCM−OCT−S32−C64

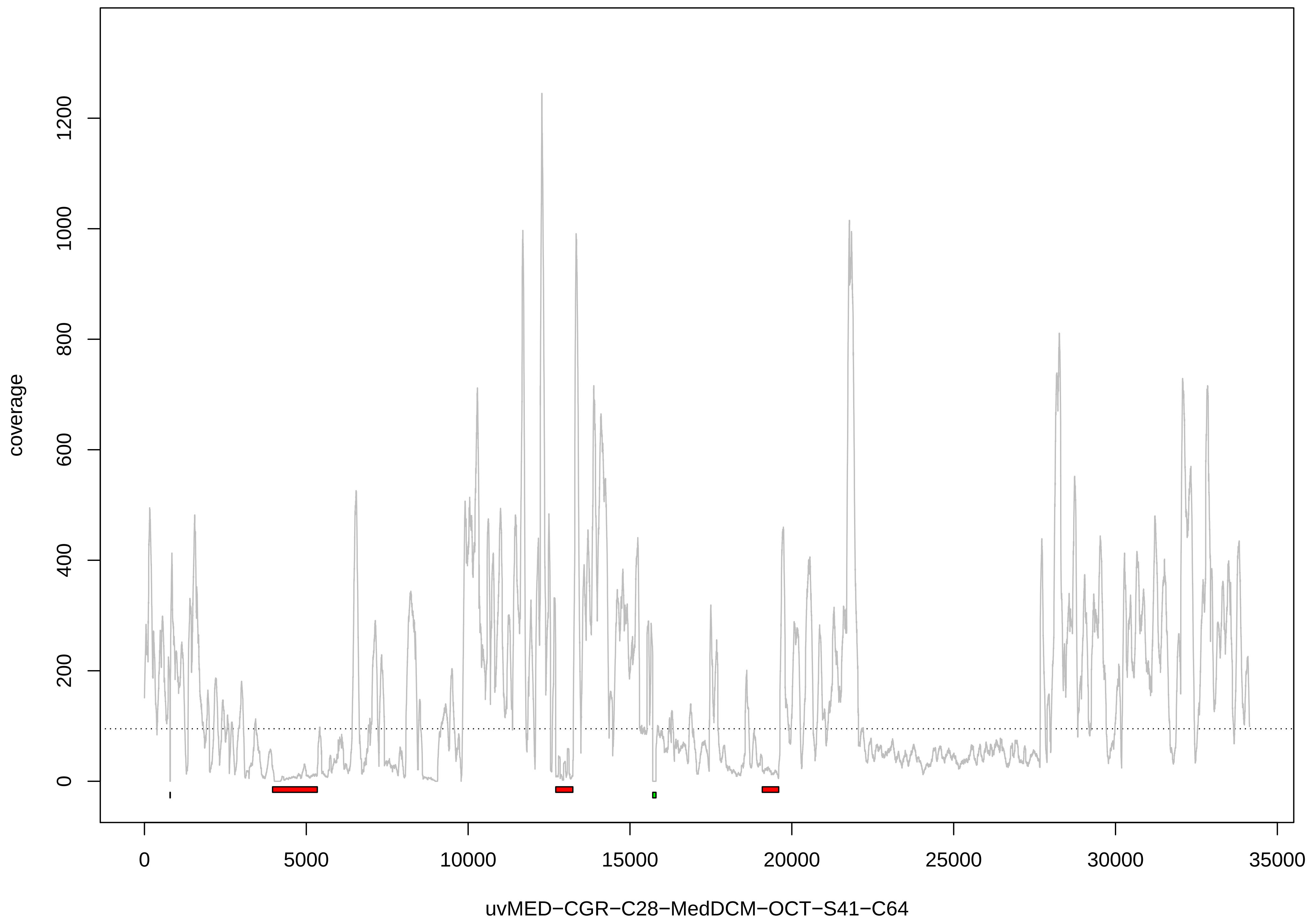
coverage
0
200
400
600
800
1000
1200
0
5000
10000
15000
20000
25000
30000
35000
uvMED−CGR−C28−MedDCM−OCT−S41−C64

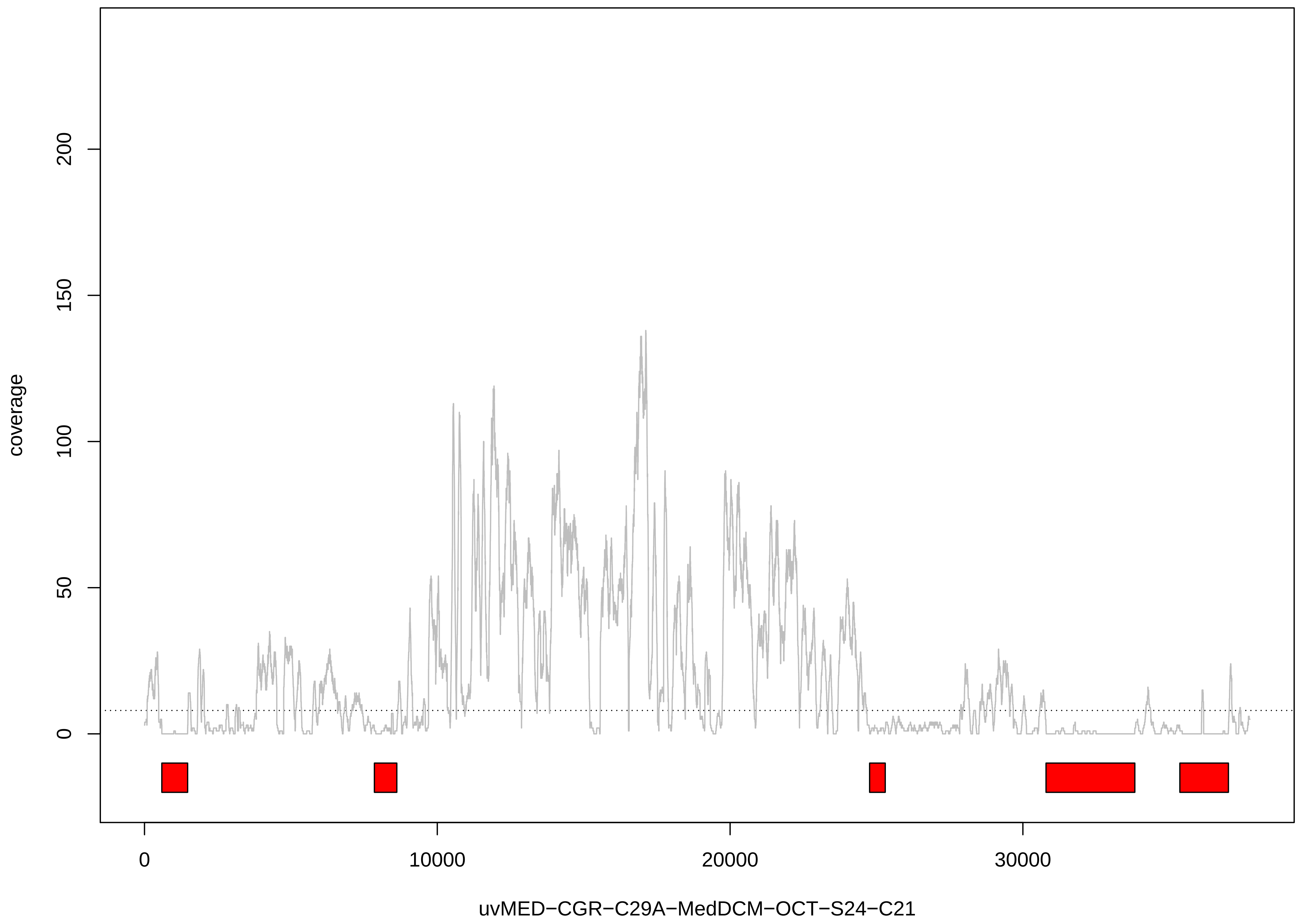

coverage
0
50
100
150
200
0
10000
20000
30000
uvMED−CGR−C29A−MedDCM−OCT−S24−C21

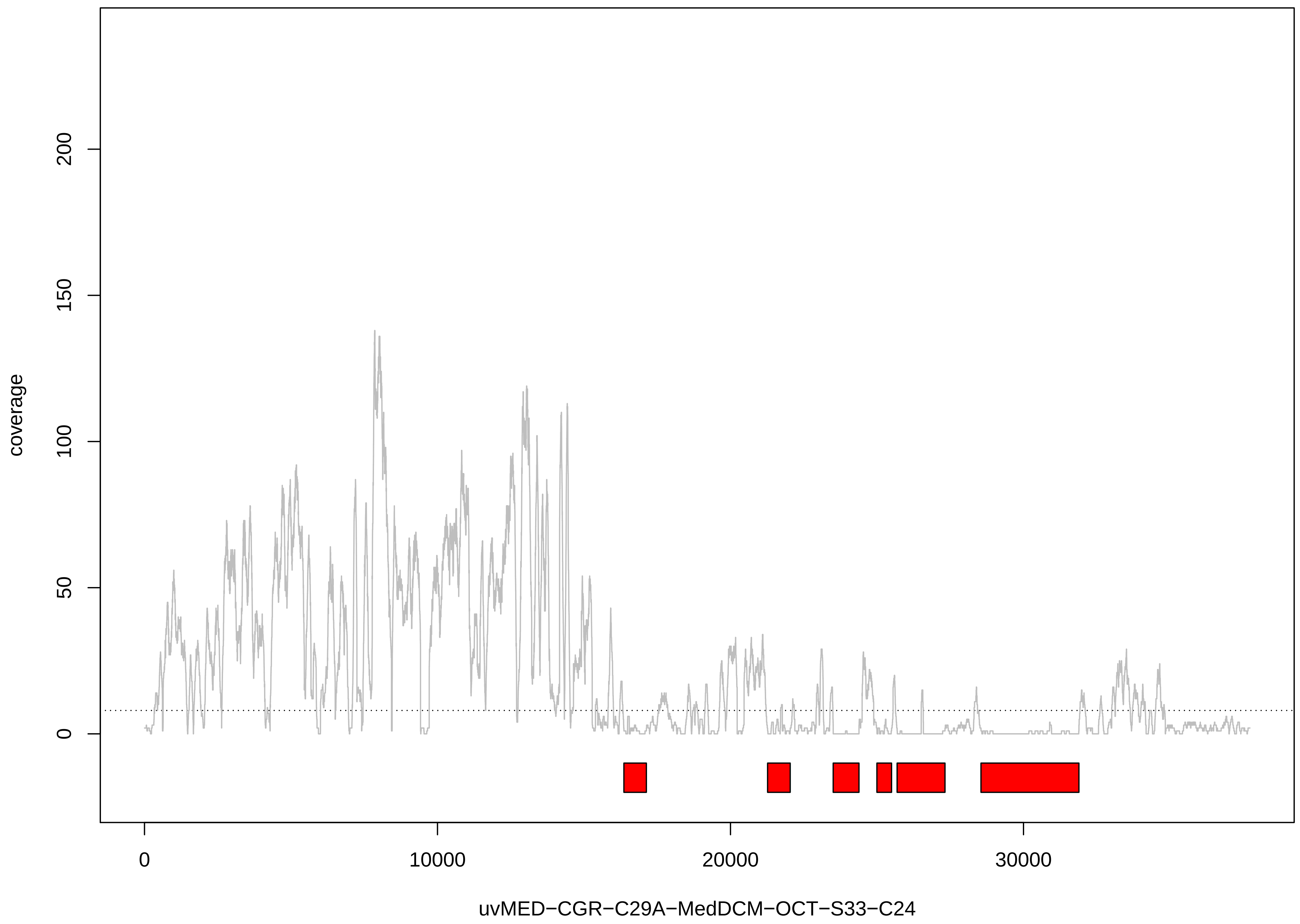
coverage
0
50
100
150
200
0
10000
20000
30000
uvMED−CGR−C29A−MedDCM−OCT−S33−C24

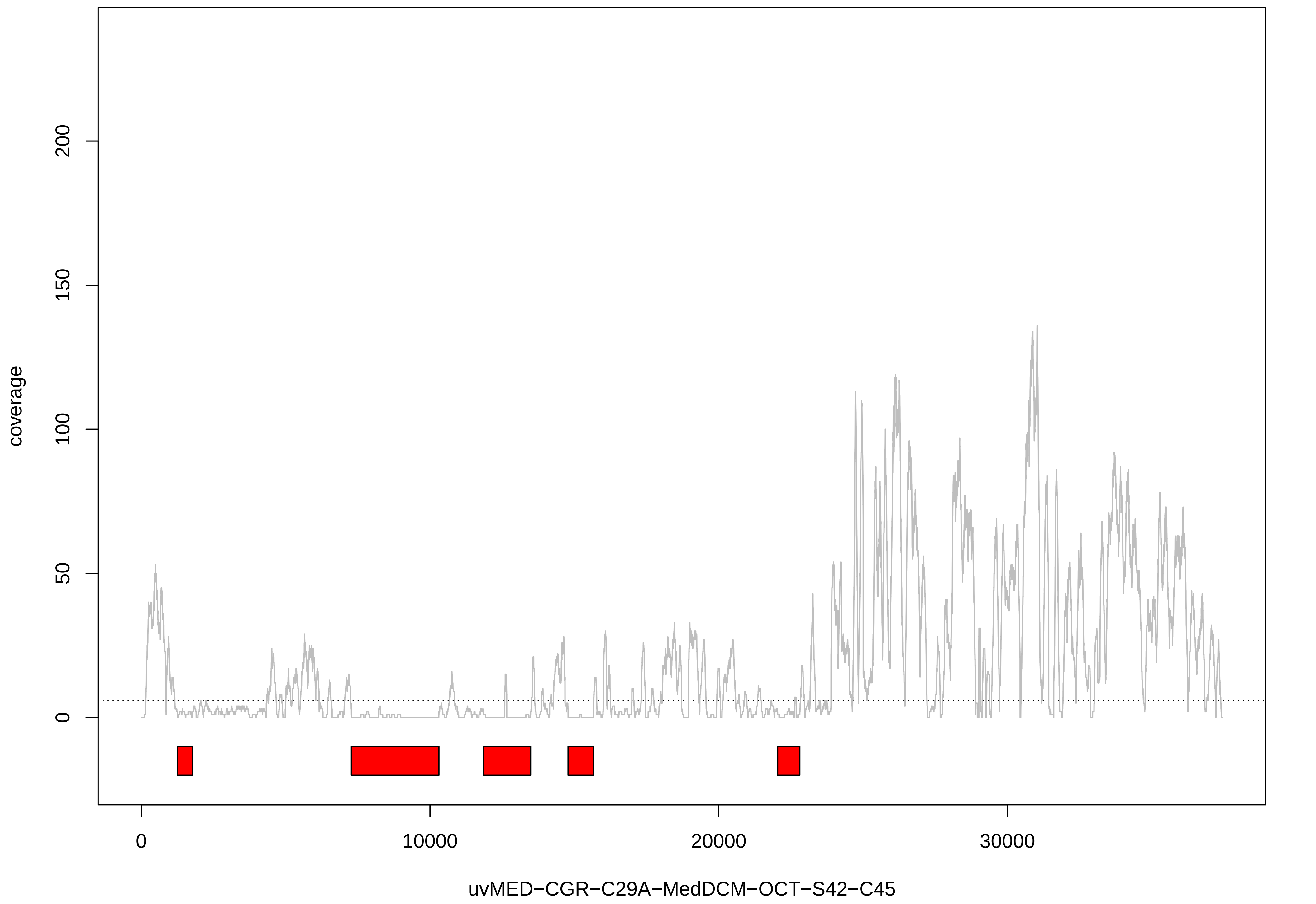

coverage
0
50
100
150
200
0
10000
20000
30000
uvMED−CGR−C29A−MedDCM−OCT−S42−C45

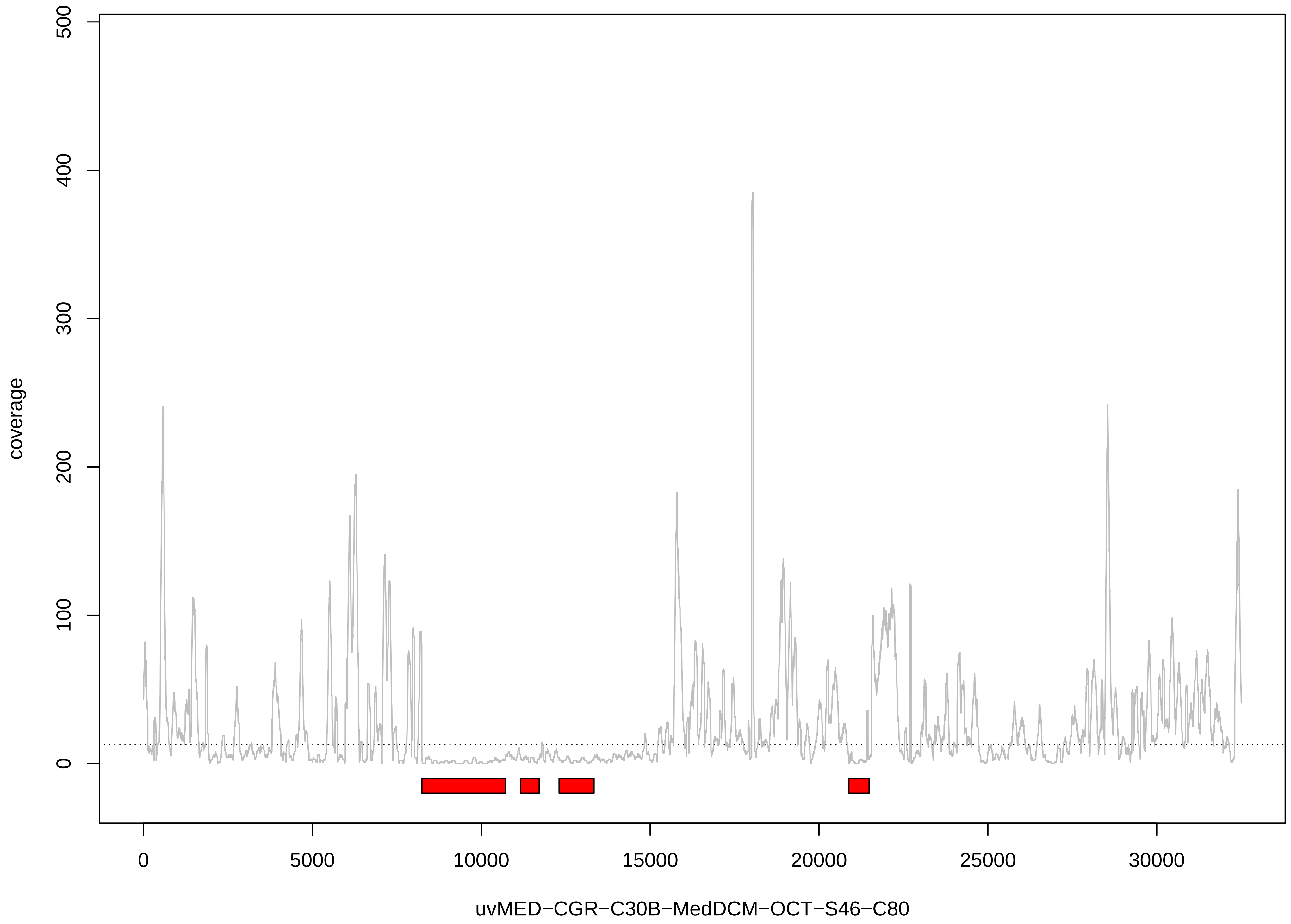

coverage
0
100
200
300
400
500
0
5000
10000
15000
20000
25000
30000
uvMED−CGR−C30B−MedDCM−OCT−S46−C80

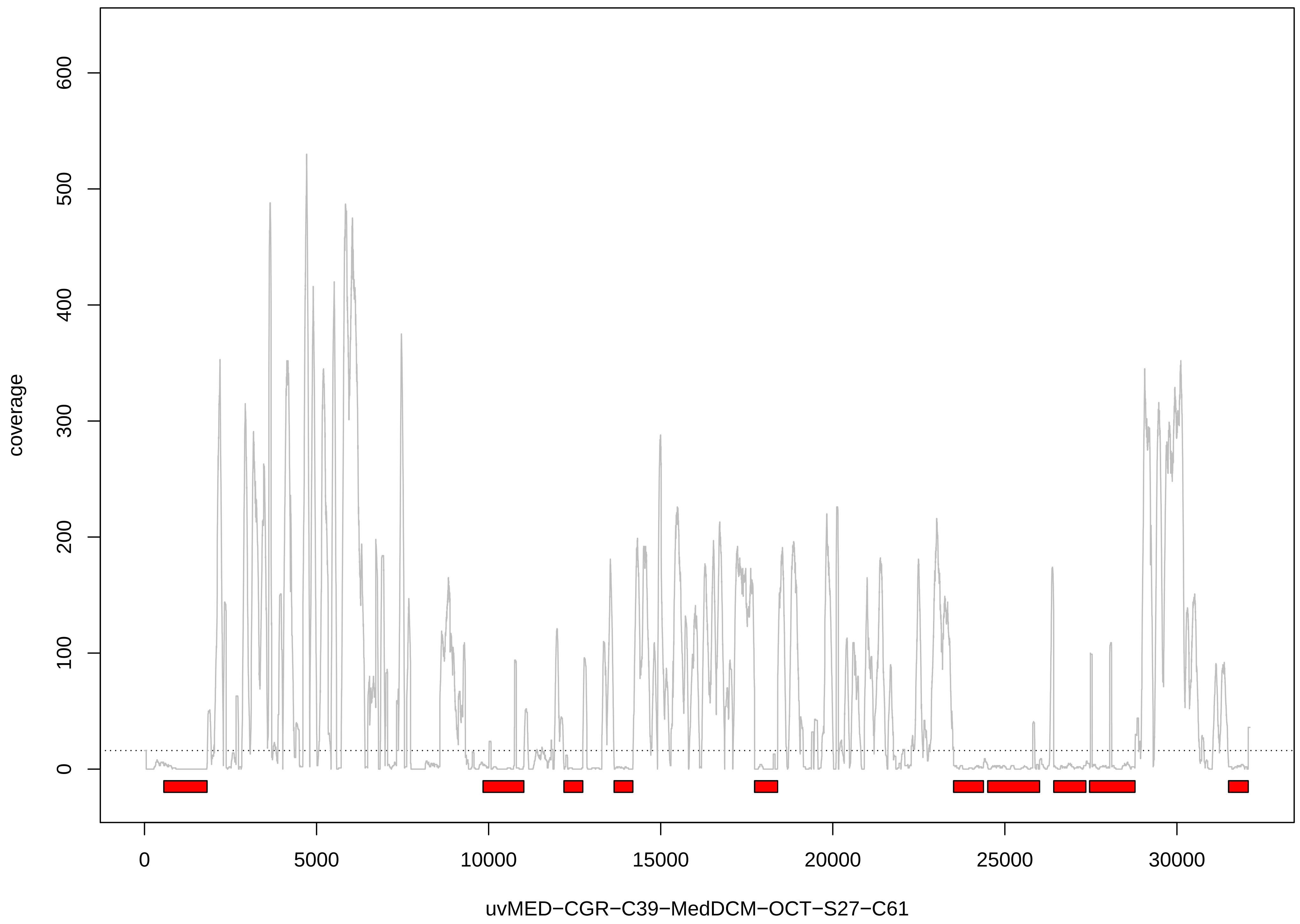

coverage
0
100
200
300
400
500
600
0
5000
10000
15000
20000
25000
30000
uvMED−CGR−C39−MedDCM−OCT−S27−C61

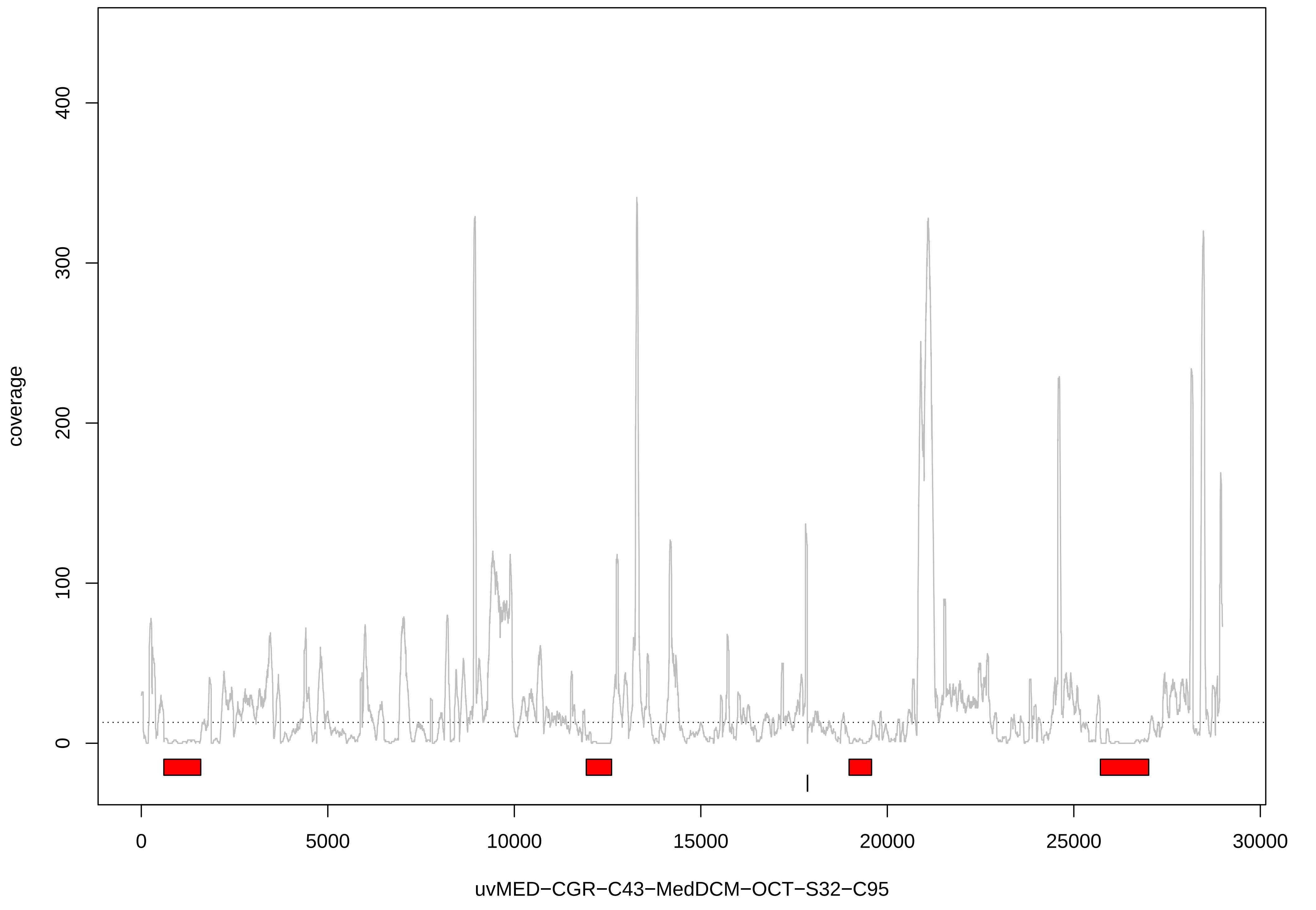

coverage
0
100
200
300
400
0
5000
10000
15000
20000
25000
30000
uvMED−CGR−C43−MedDCM−OCT−S32−C95

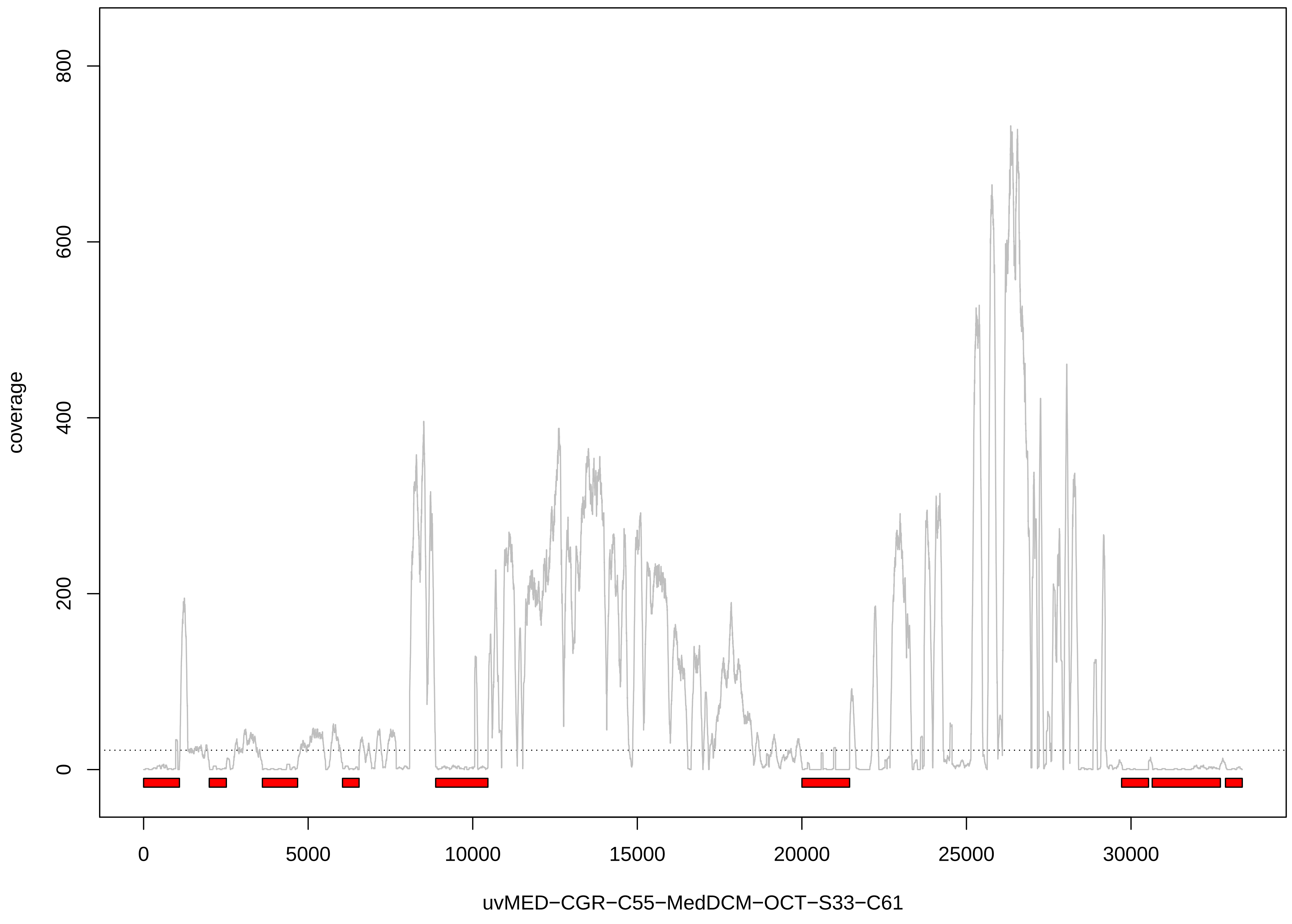

coverage
800
600
400
200
0
0
5000
10000
15000
20000
25000
30000
uvMED−CGR−C55−MedDCM−OCT−S33−C61

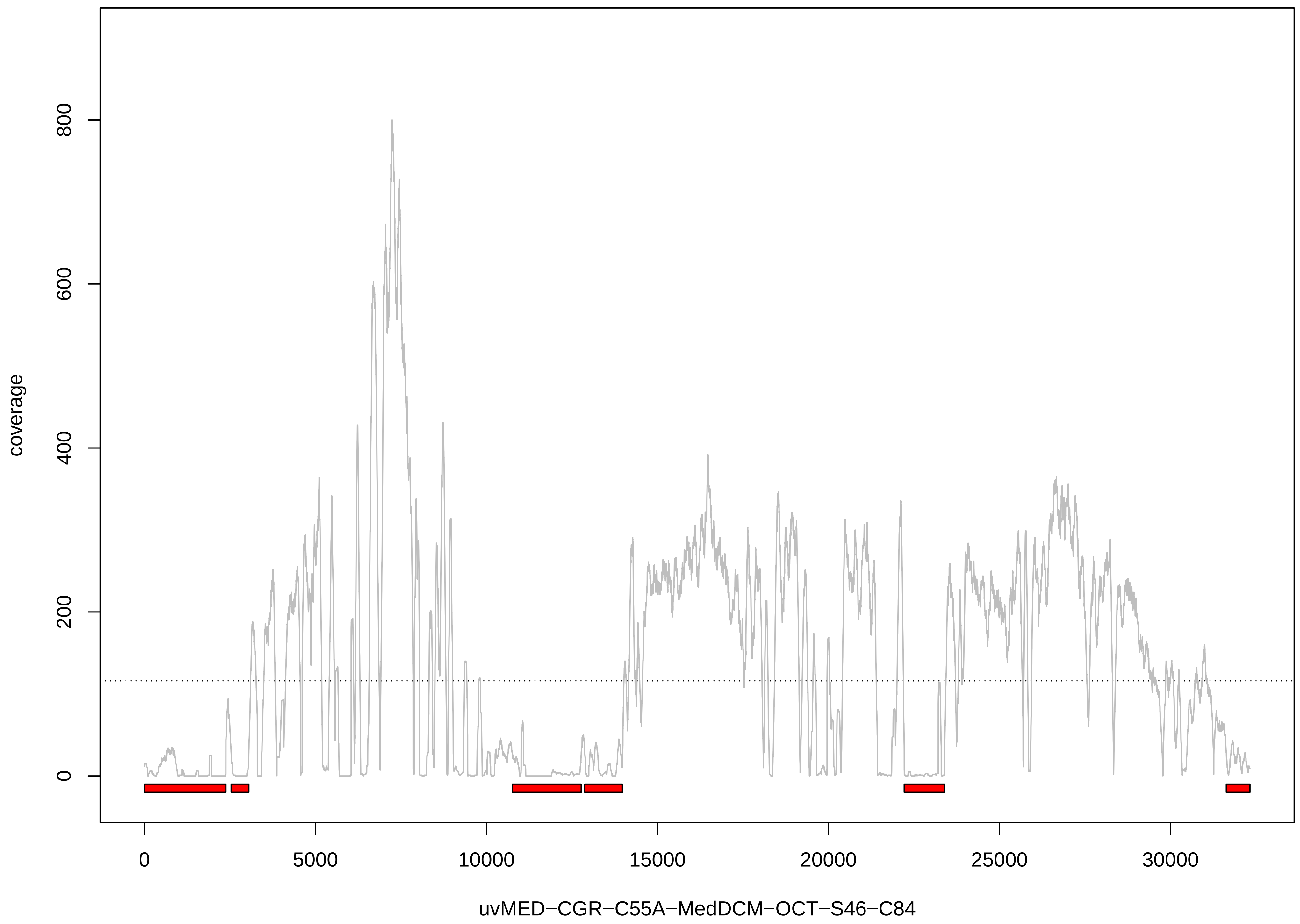

coverage
800
600
400
200
0
0
5000
10000
15000
20000
25000
30000
uvMED−CGR−C55A−MedDCM−OCT−S46−C84

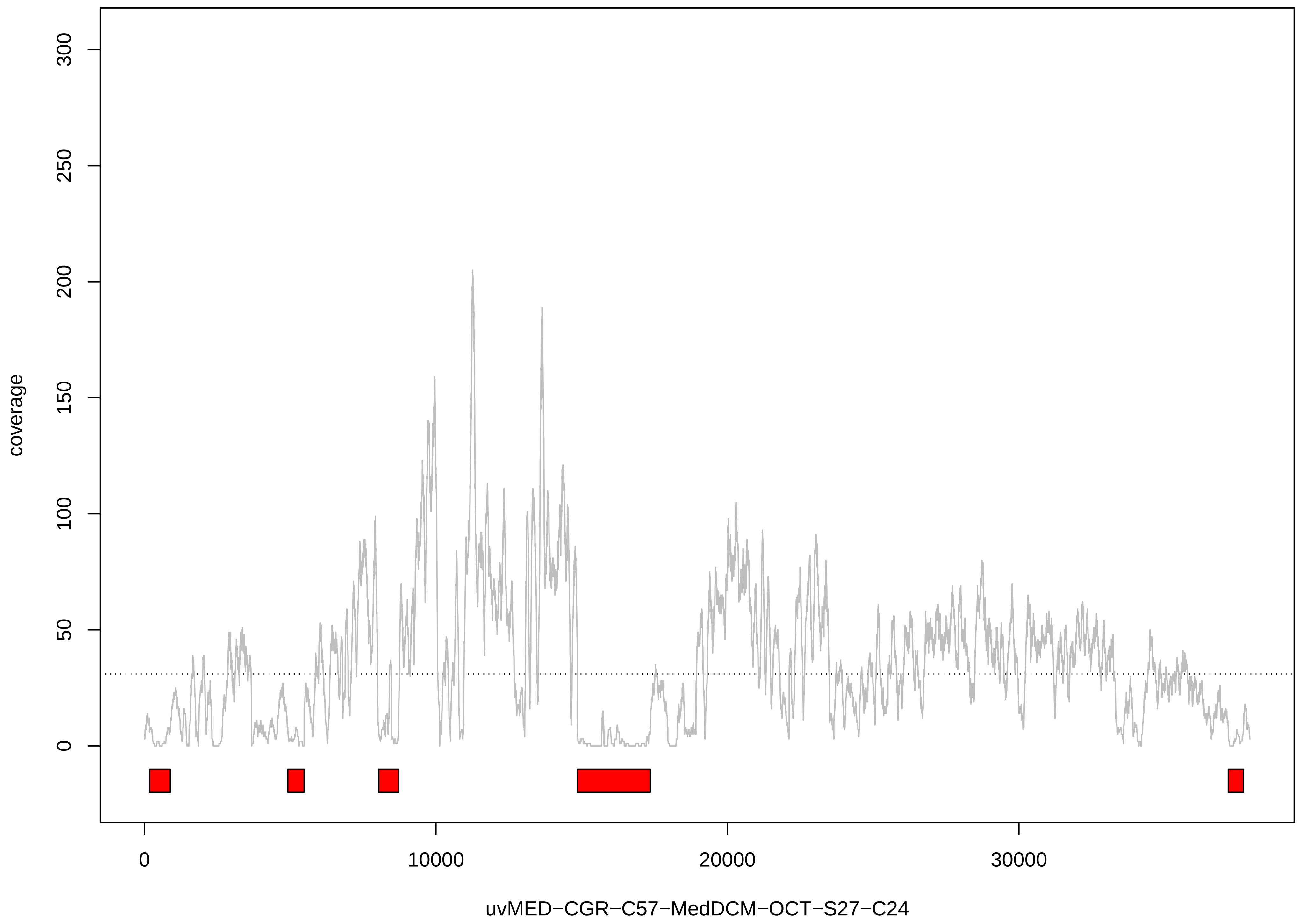

coverage
0
50
100
150
200
250
300
0
10000
20000
30000
uvMED−CGR−C57−MedDCM−OCT−S27−C24

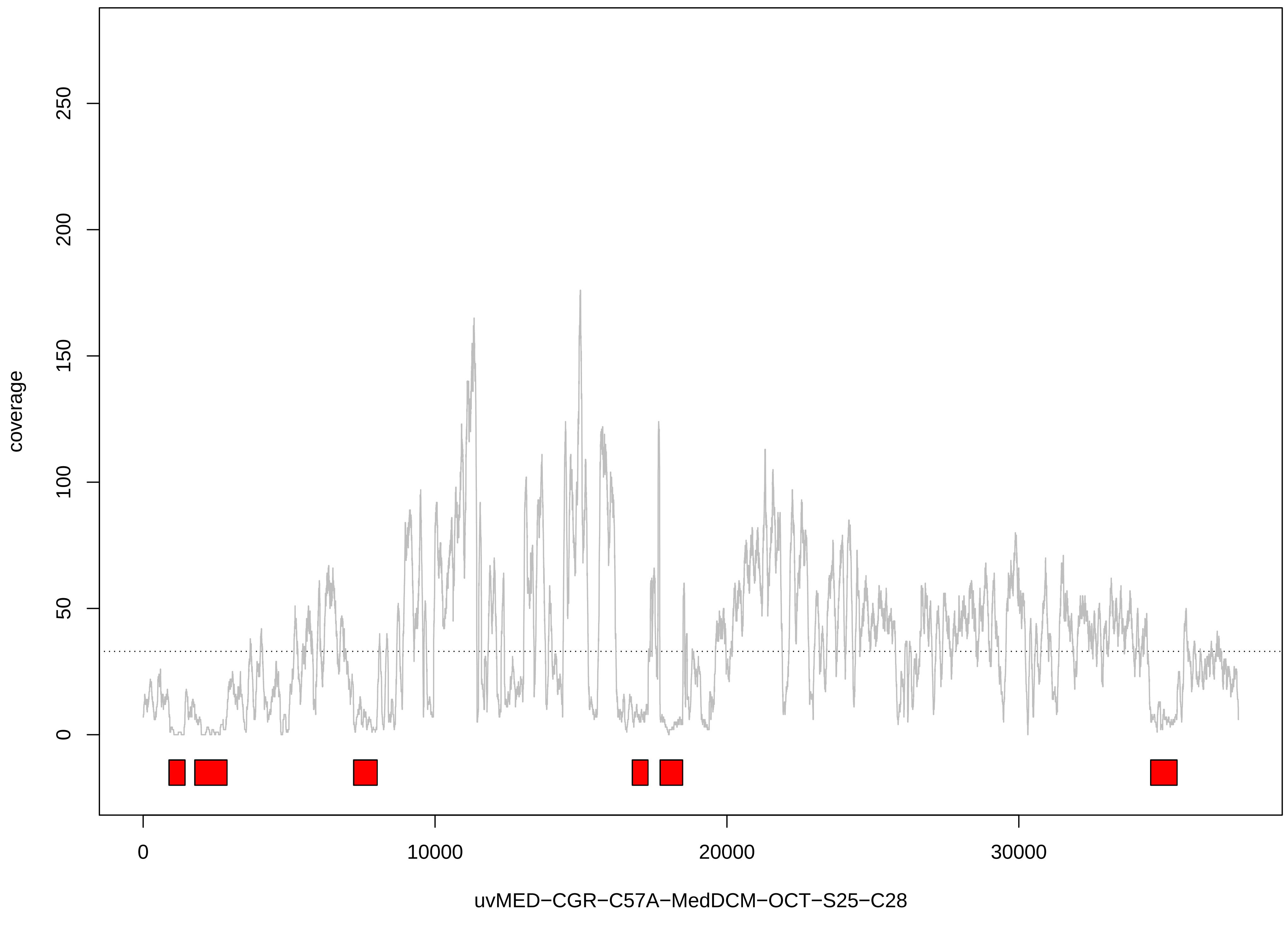

coverage
0
50
100
150
200
250
0
10000
20000
30000
uvMED−CGR−C57A−MedDCM−OCT−S25−C28

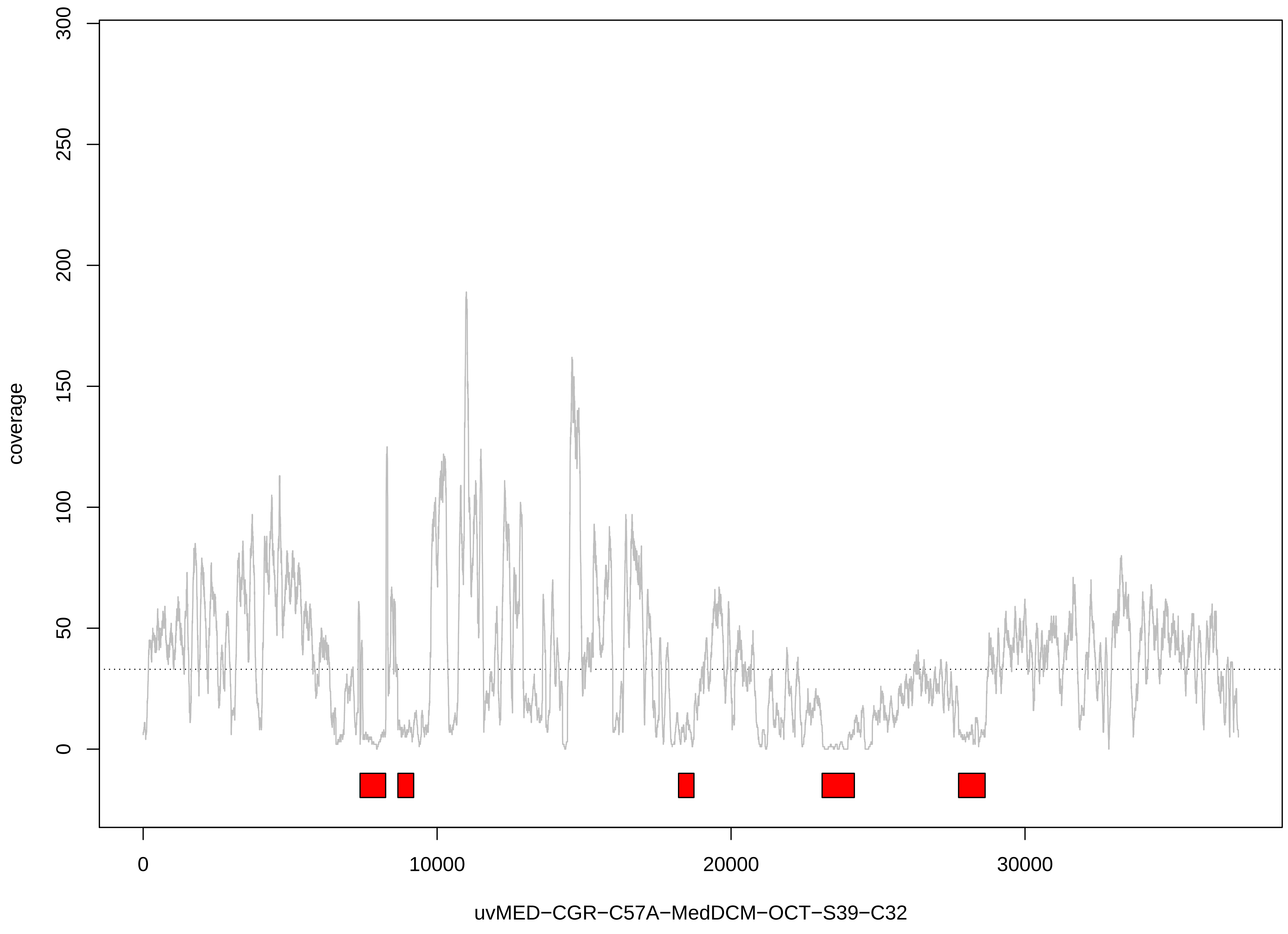

coverage
0
50
100
150
200
250
300
0
10000
20000
30000
uvMED−CGR−C57A−MedDCM−OCT−S39−C32

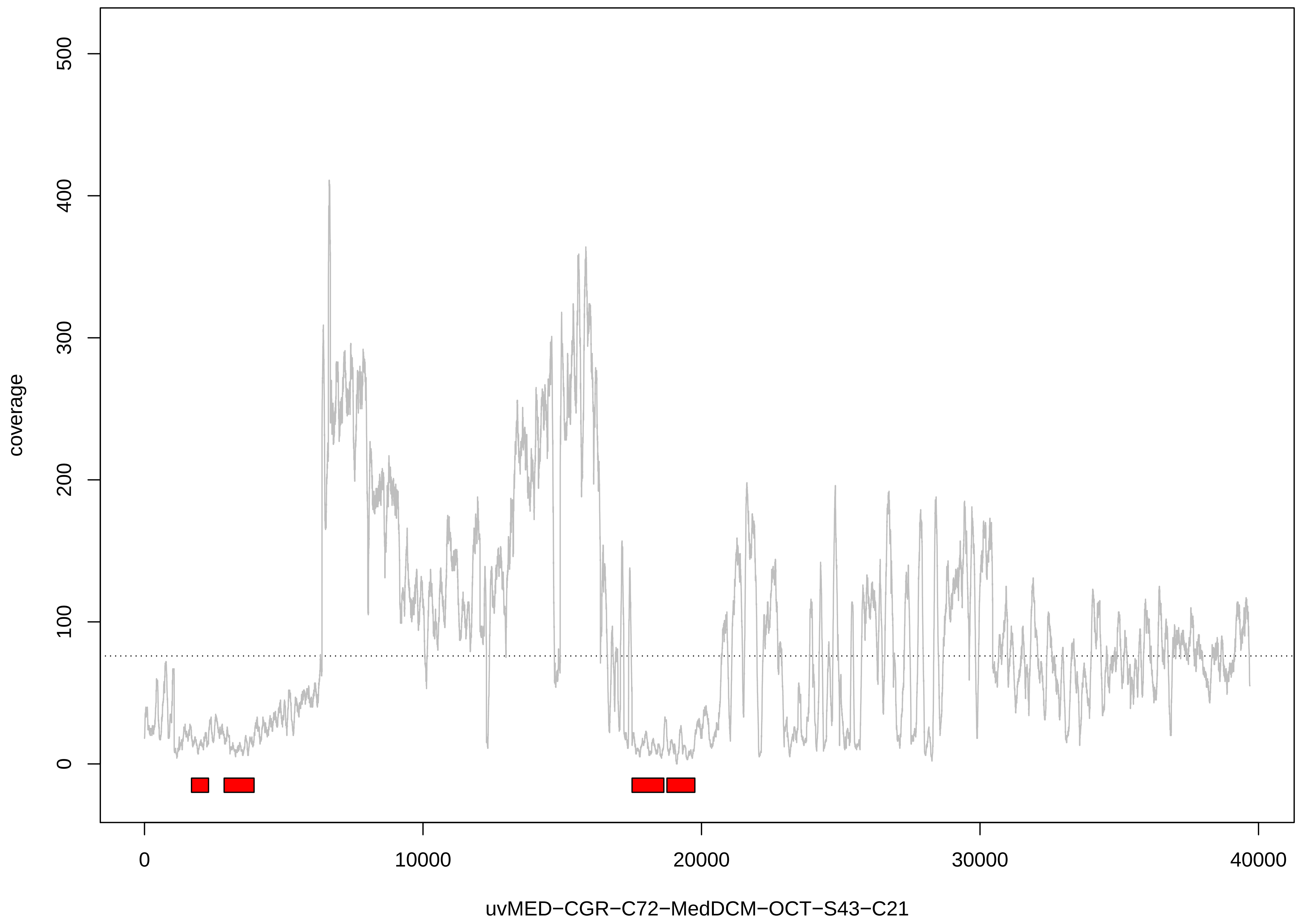
coverage
0
100
200
300
400
500
0
10000
20000
30000
40000
uvMED−CGR−C72−MedDCM−OCT−S43−C21

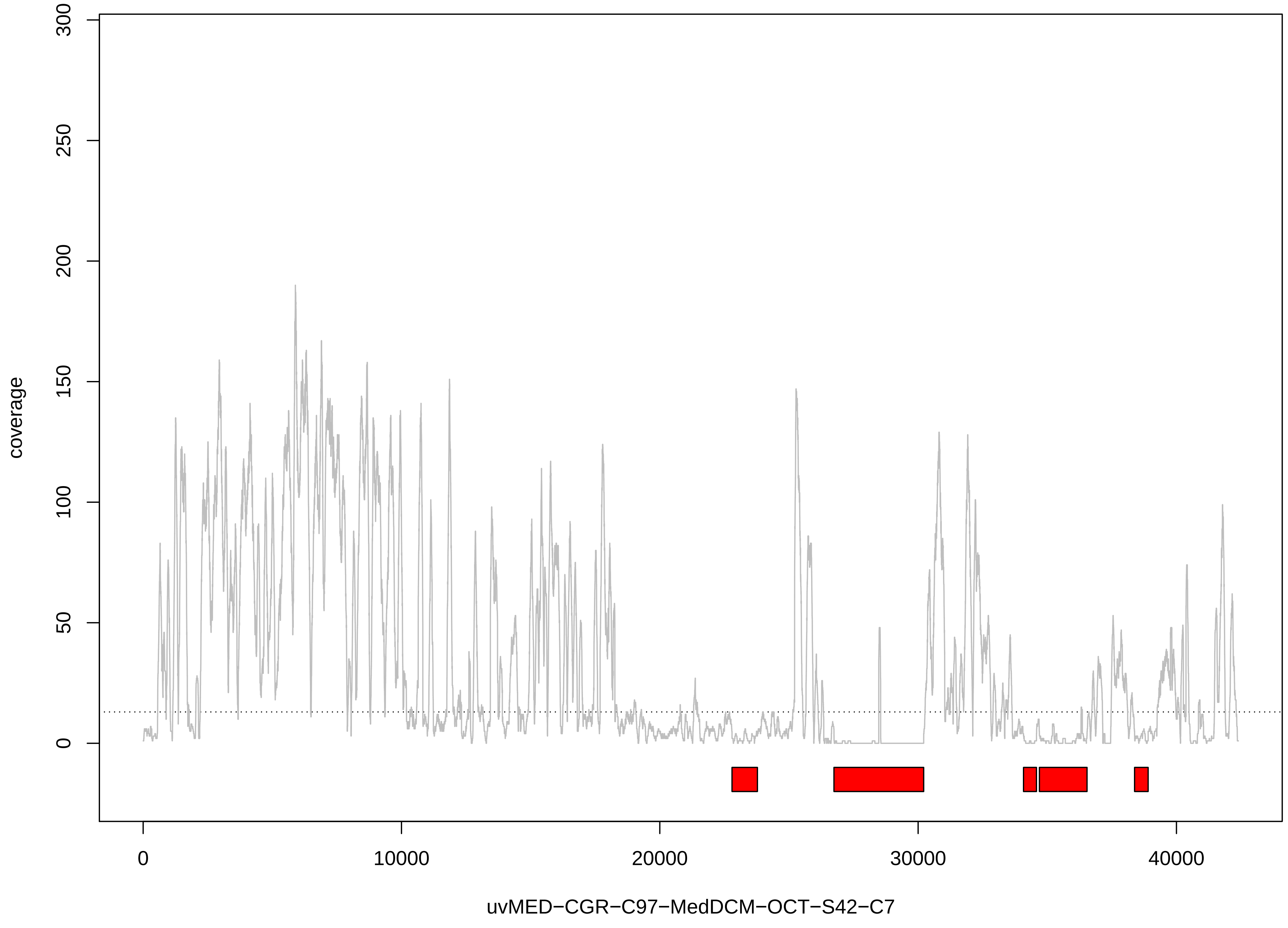

coverage
0
50
100
150
200
250
300
0
10000
20000
30000
40000
uvMED−CGR−C97−MedDCM−OCT−S42−C7

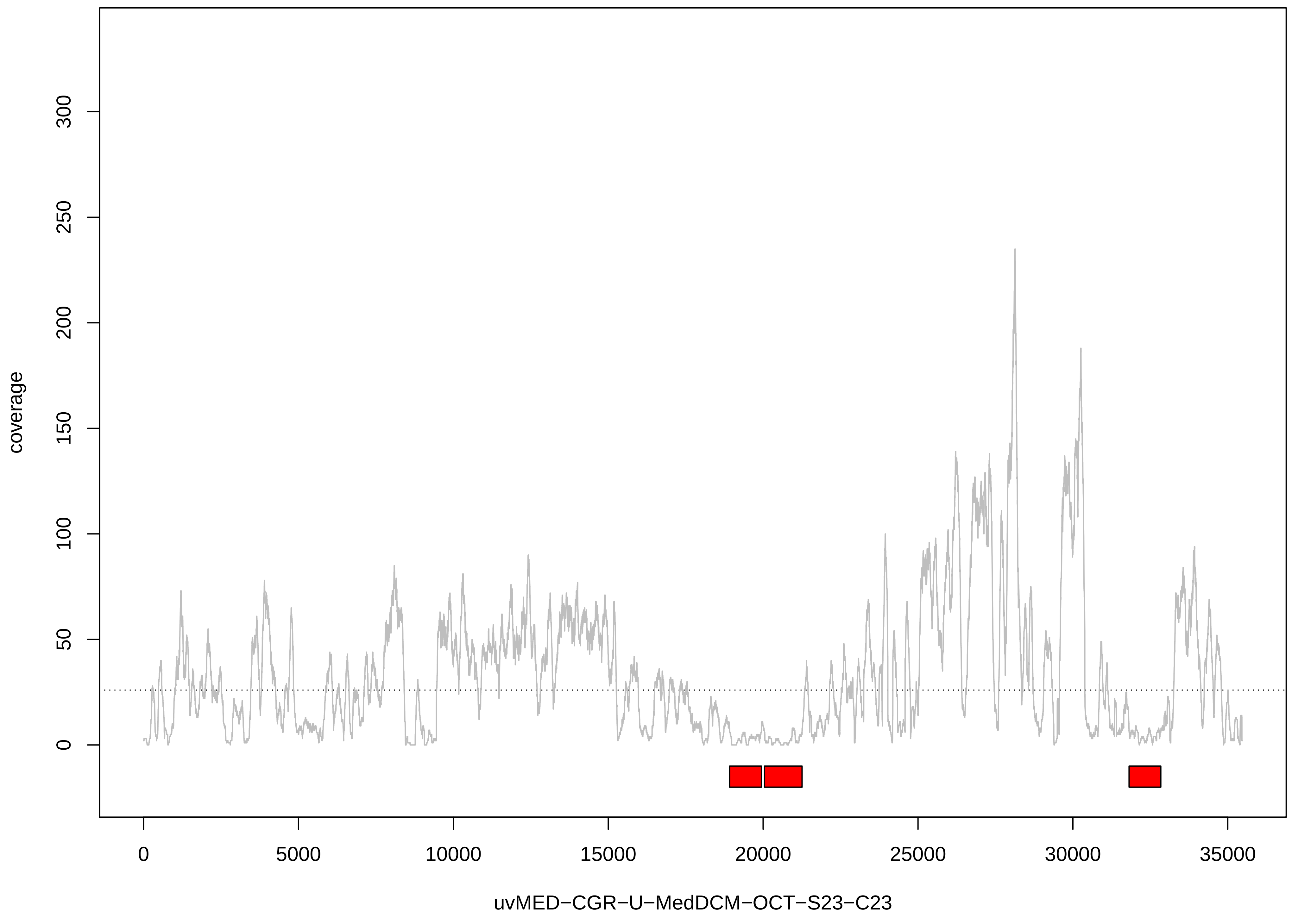

coverage
0
50
100
150
200
250
300
0
5000
10000
15000
20000
25000
30000
35000
uvMED−CGR−U−MedDCM−OCT−S23−C23

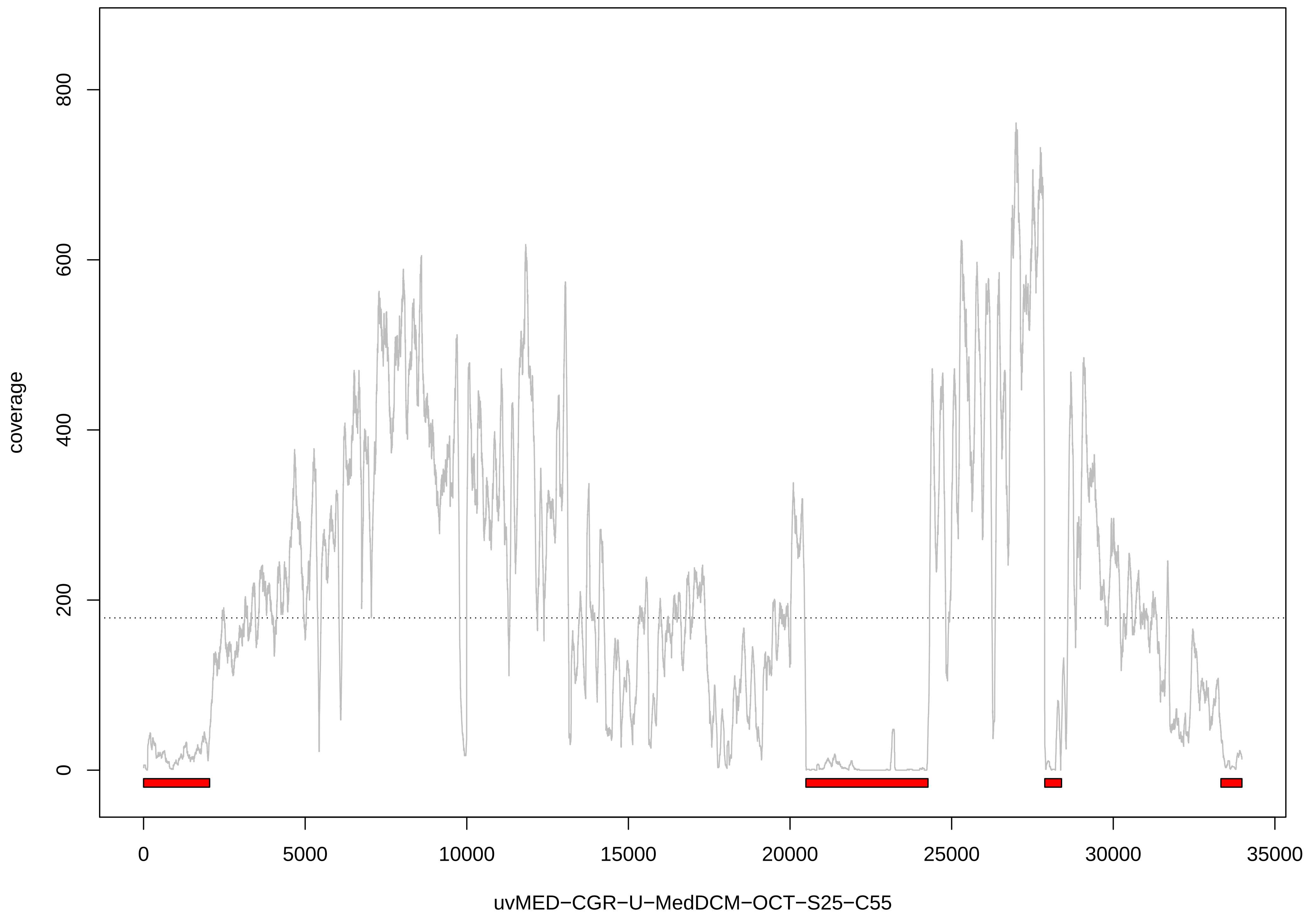

coverage
0
200
400
600
800
0
5000
10000
15000
20000
25000
30000
35000
uvMED−CGR−U−MedDCM−OCT−S25−C55

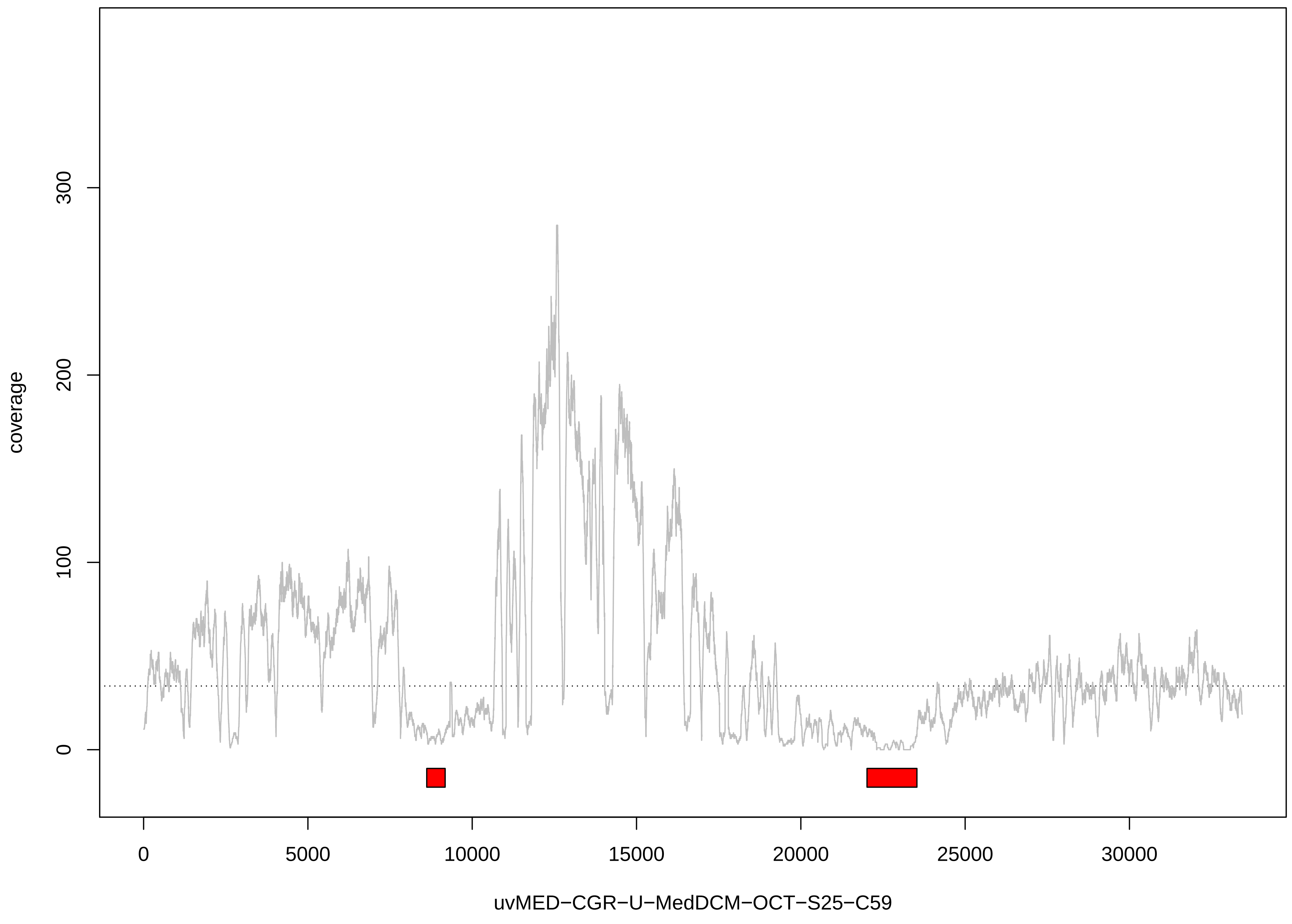

coverage
0
100
200
300
0
5000
10000
15000
20000
25000
30000
uvMED−CGR−U−MedDCM−OCT−S25−C59

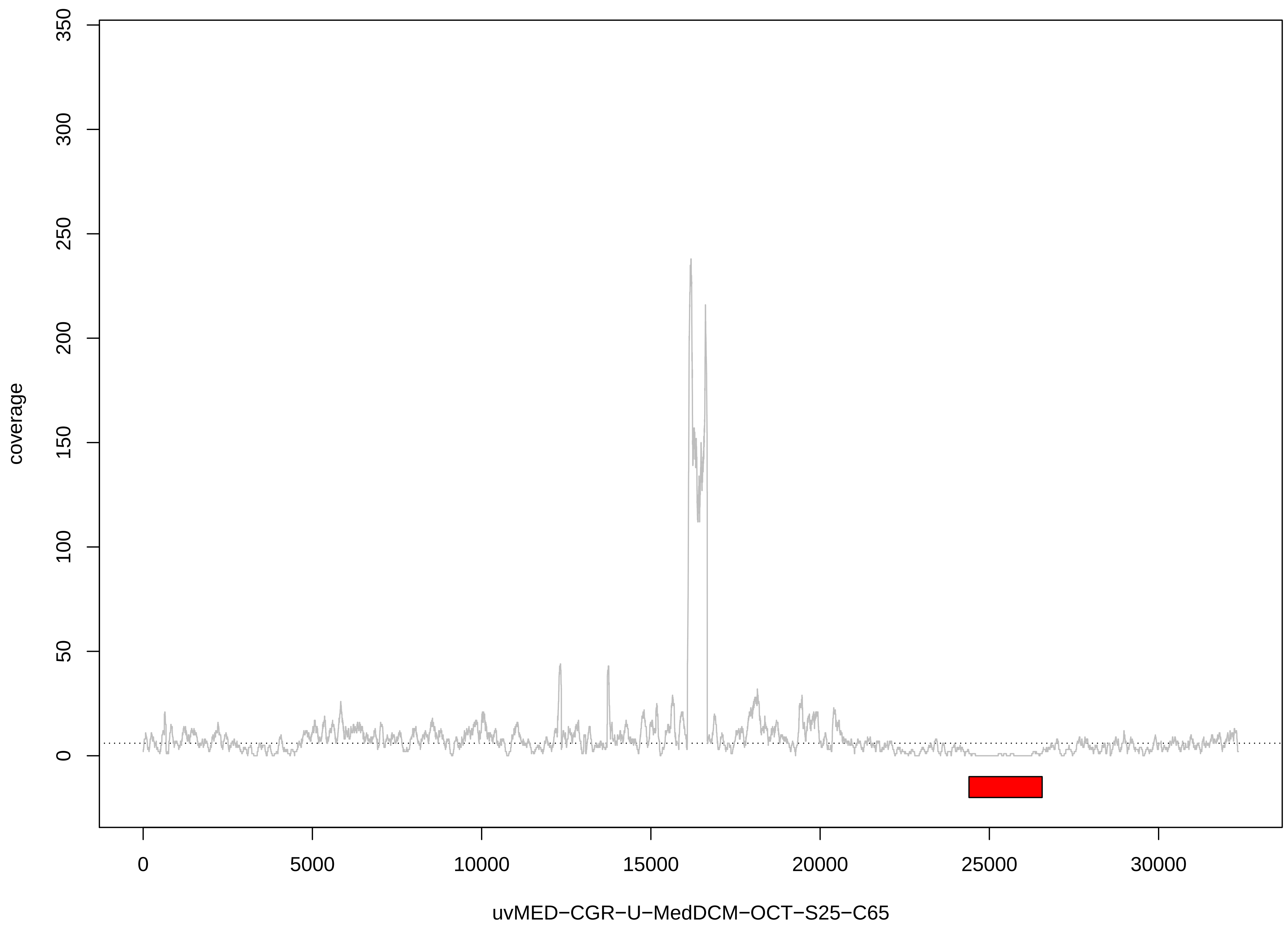
coverage
0
50
100
150
200
250
300
350
0
5000
10000
15000
20000
25000
30000
uvMED−CGR−U−MedDCM−OCT−S25−C65

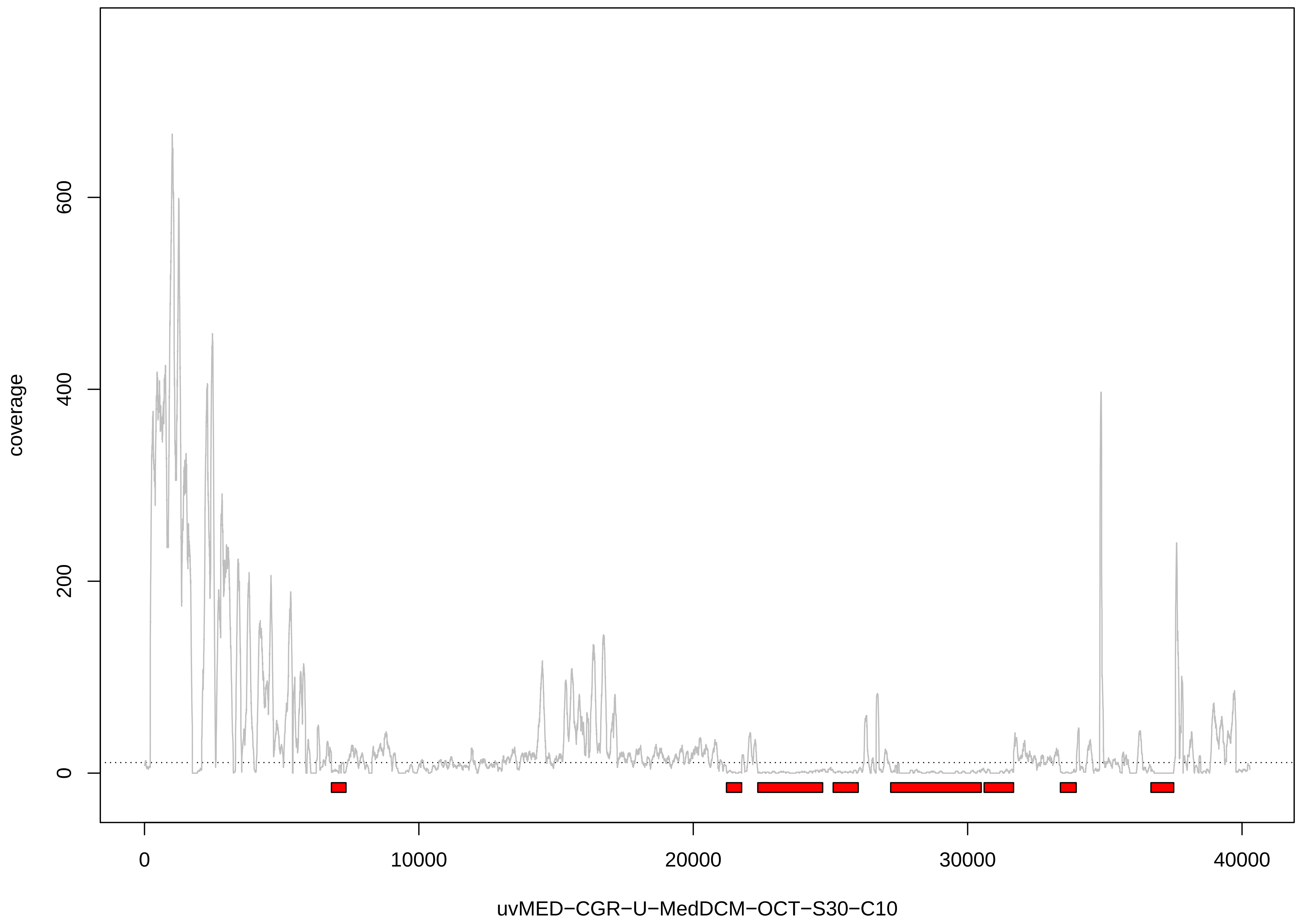

coverage
0
200
400
600
0
10000
20000
30000
40000
uvMED−CGR−U−MedDCM−OCT−S30−C10

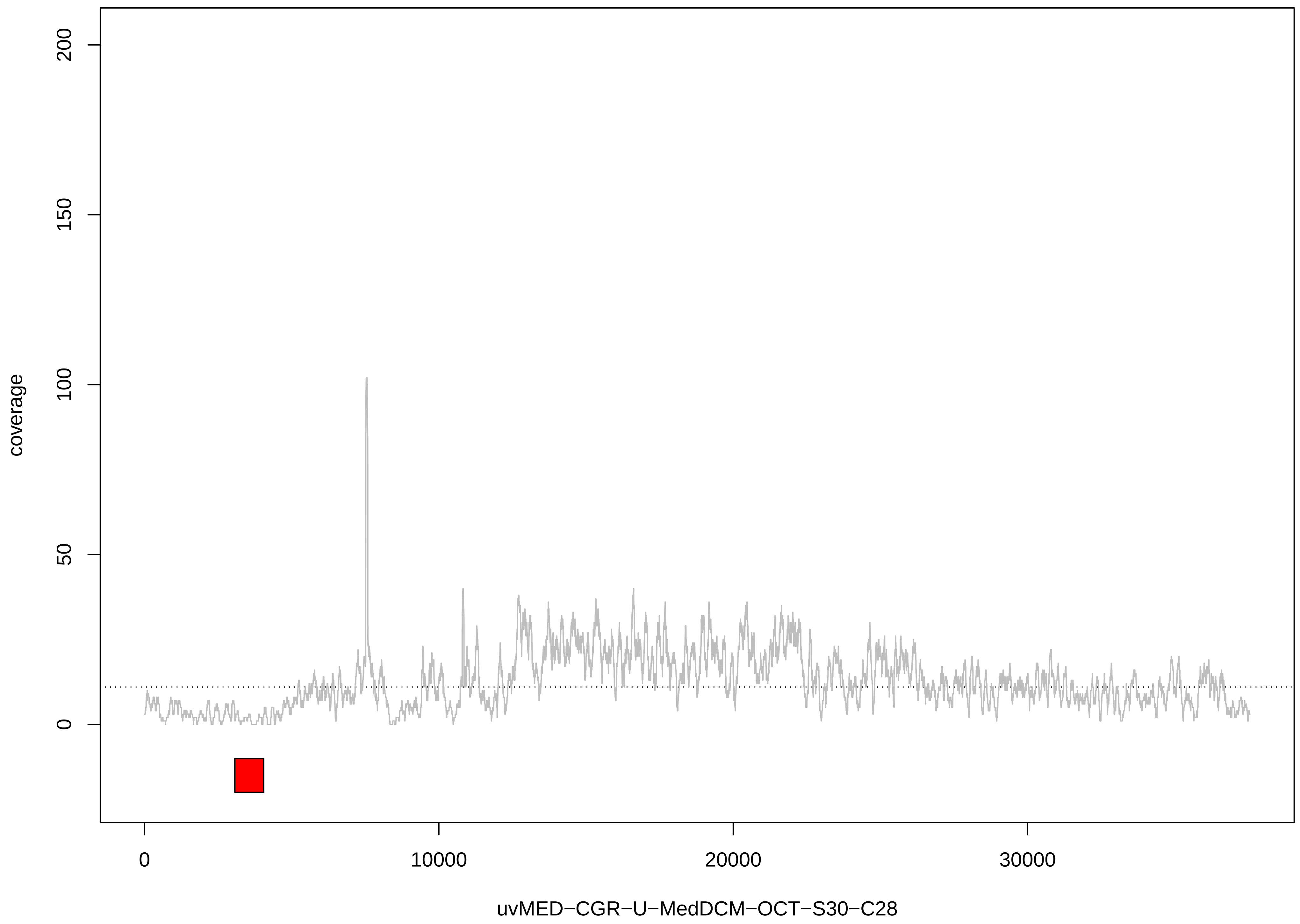

200
150
100
50
0
coverage
0
10000
20000
30000
uvMED−CGR−U−MedDCM−OCT−S30−C28

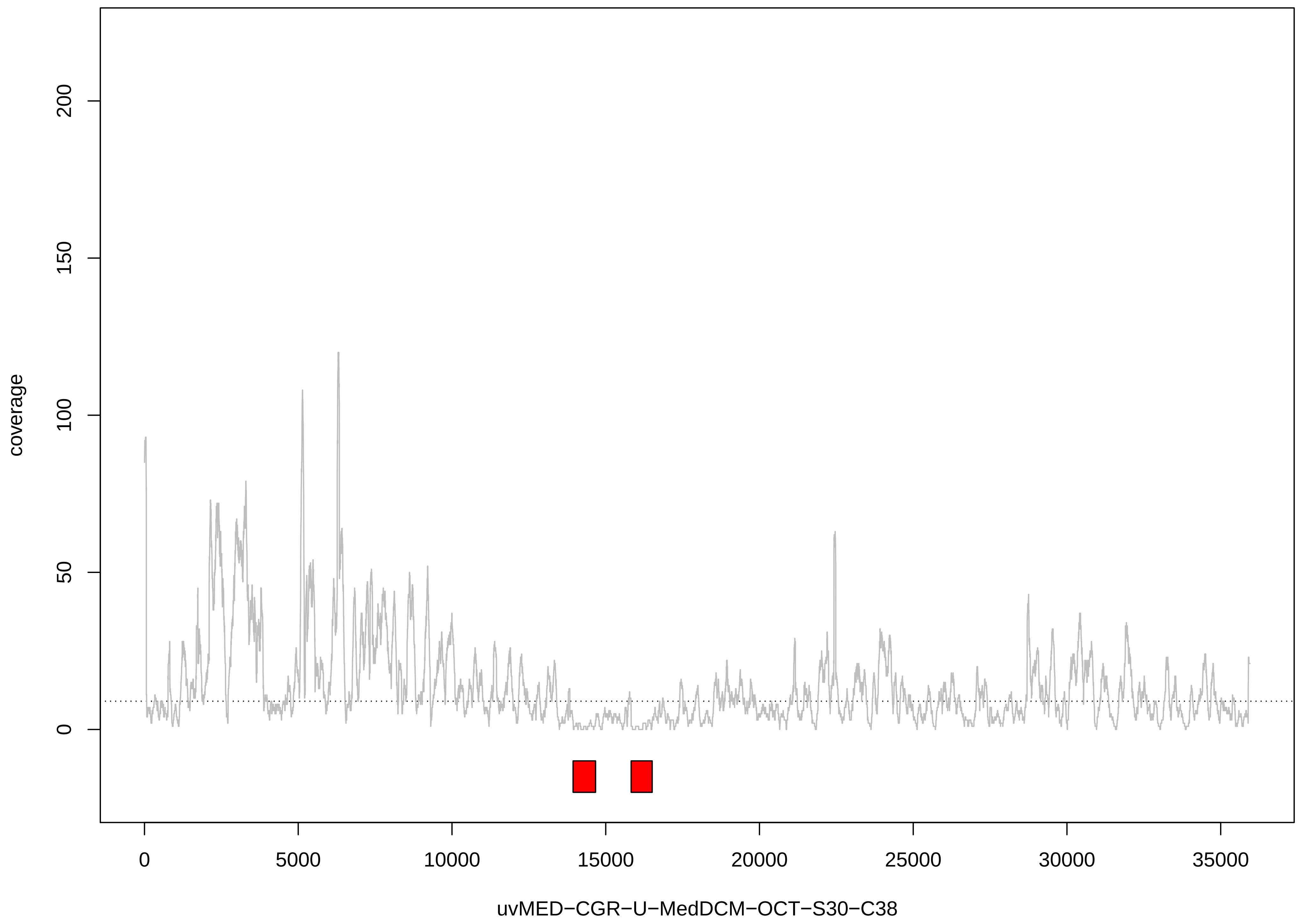
coverage
0
50
100
150
200
0
5000
10000
15000
20000
25000
30000
35000
uvMED−CGR−U−MedDCM−OCT−S30−C38

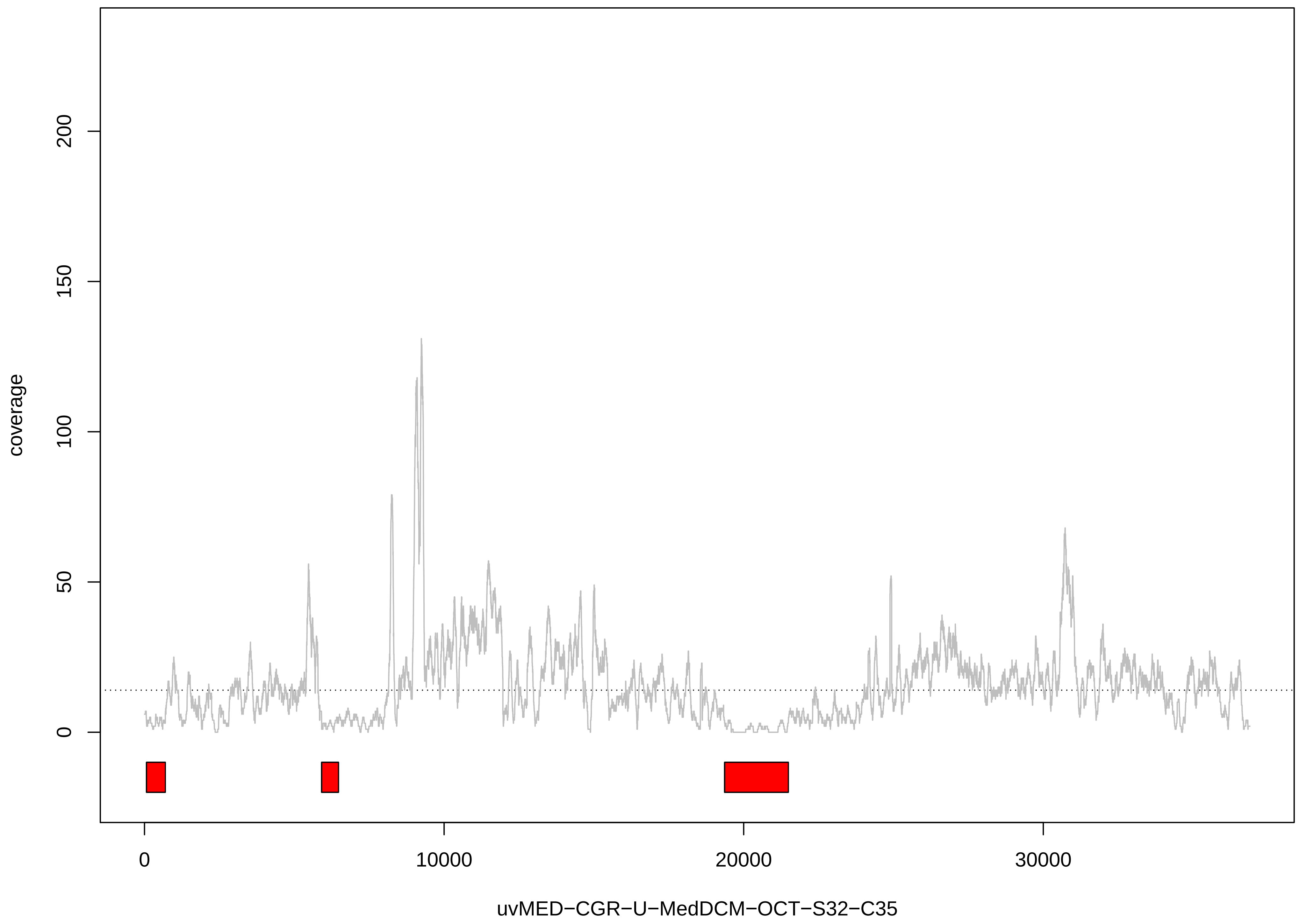
coverage
0
50
100
150
200
0
10000
20000
30000
uvMED−CGR−U−MedDCM−OCT−S32−C35

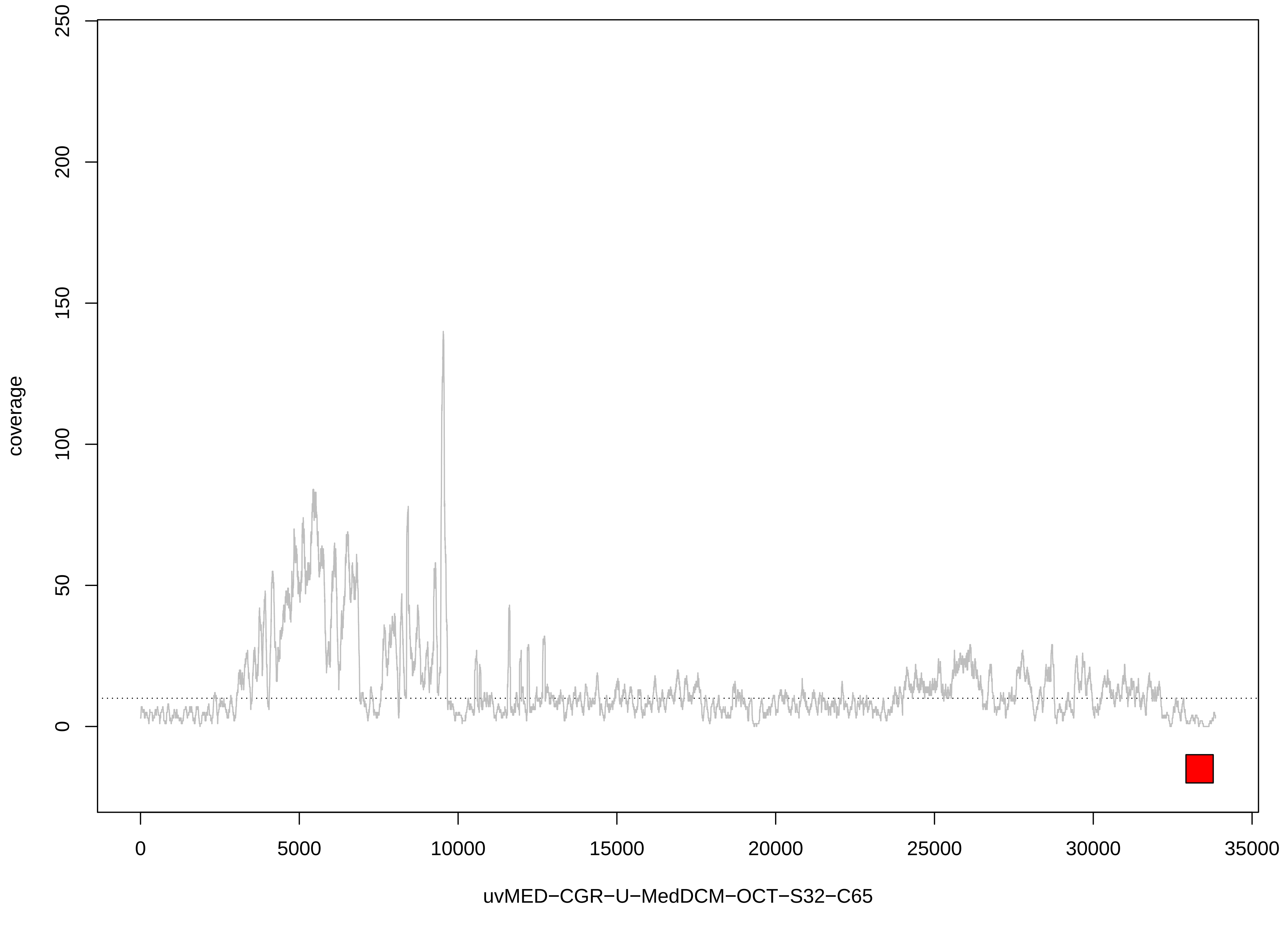

coverage
0
50
100
150
200
250
0
5000
10000
15000
20000
25000
30000
35000
uvMED−CGR−U−MedDCM−OCT−S32−C65

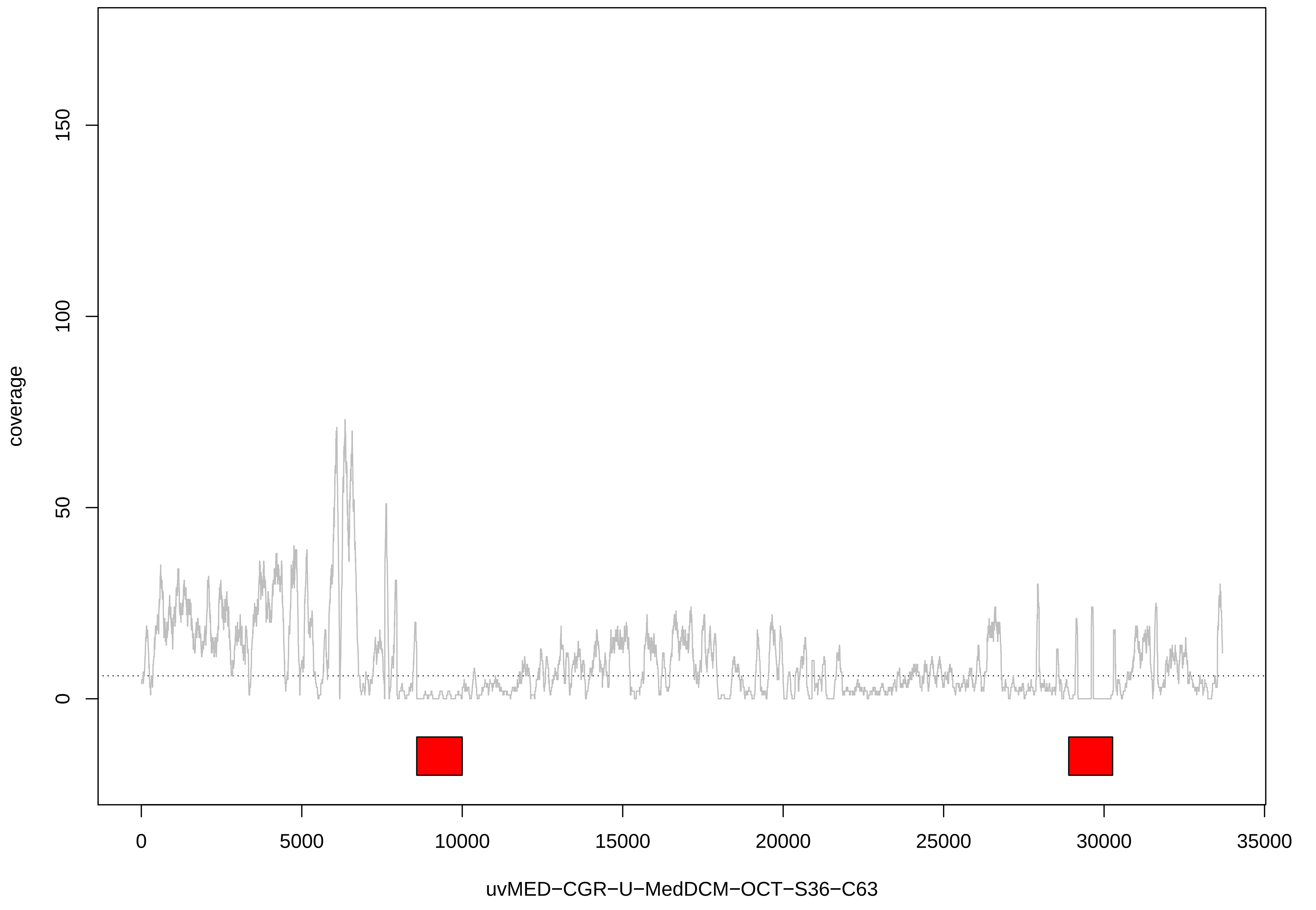

coverage
0
50
100
150
0
5000
10000
15000
20000
25000
30000
35000
uvMED−CGR−U−MedDCM−OCT−S36−C63

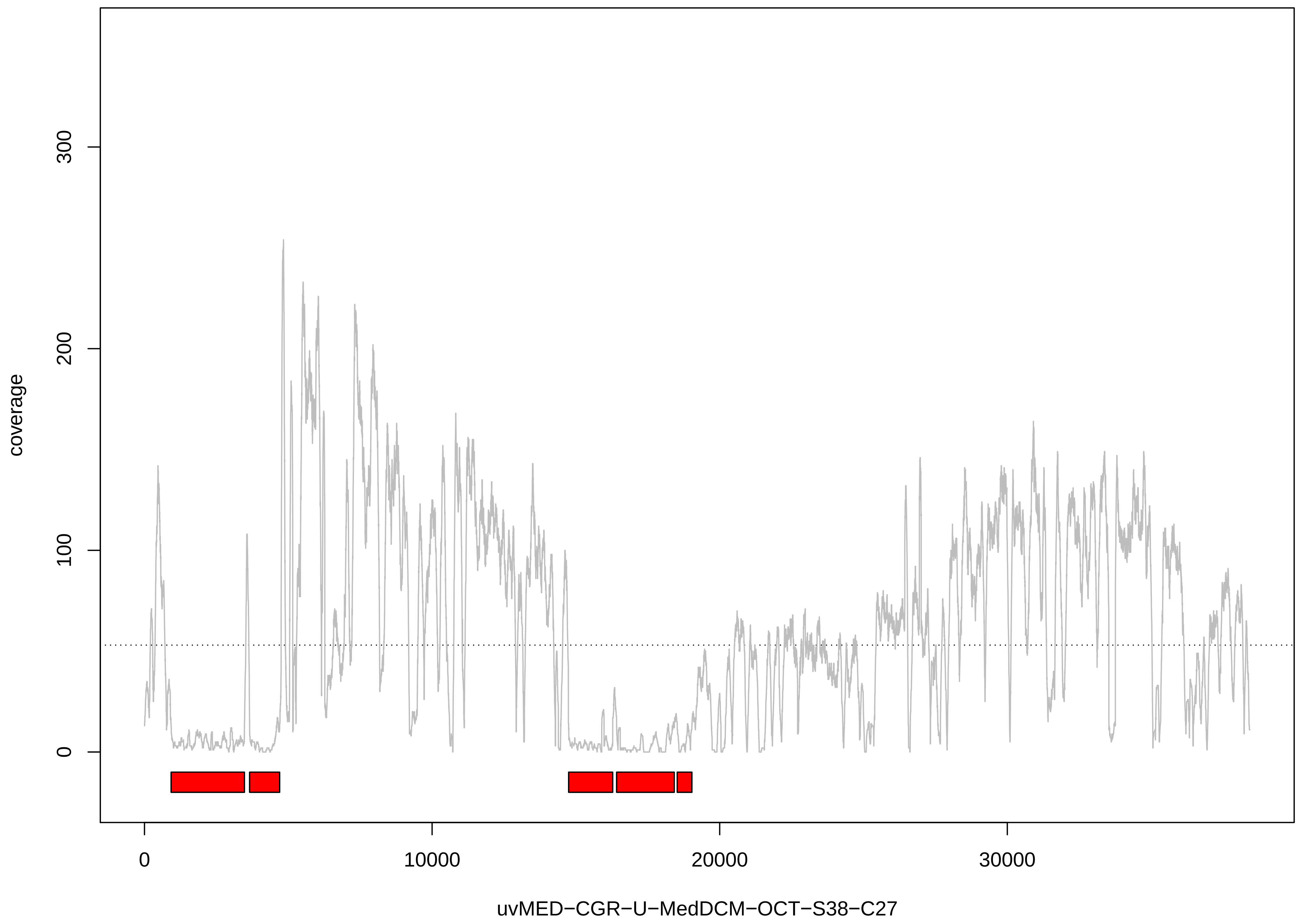
coverage
0
100
200
300
0
10000
20000
30000
uvMED−CGR−U−MedDCM−OCT−S38−C27

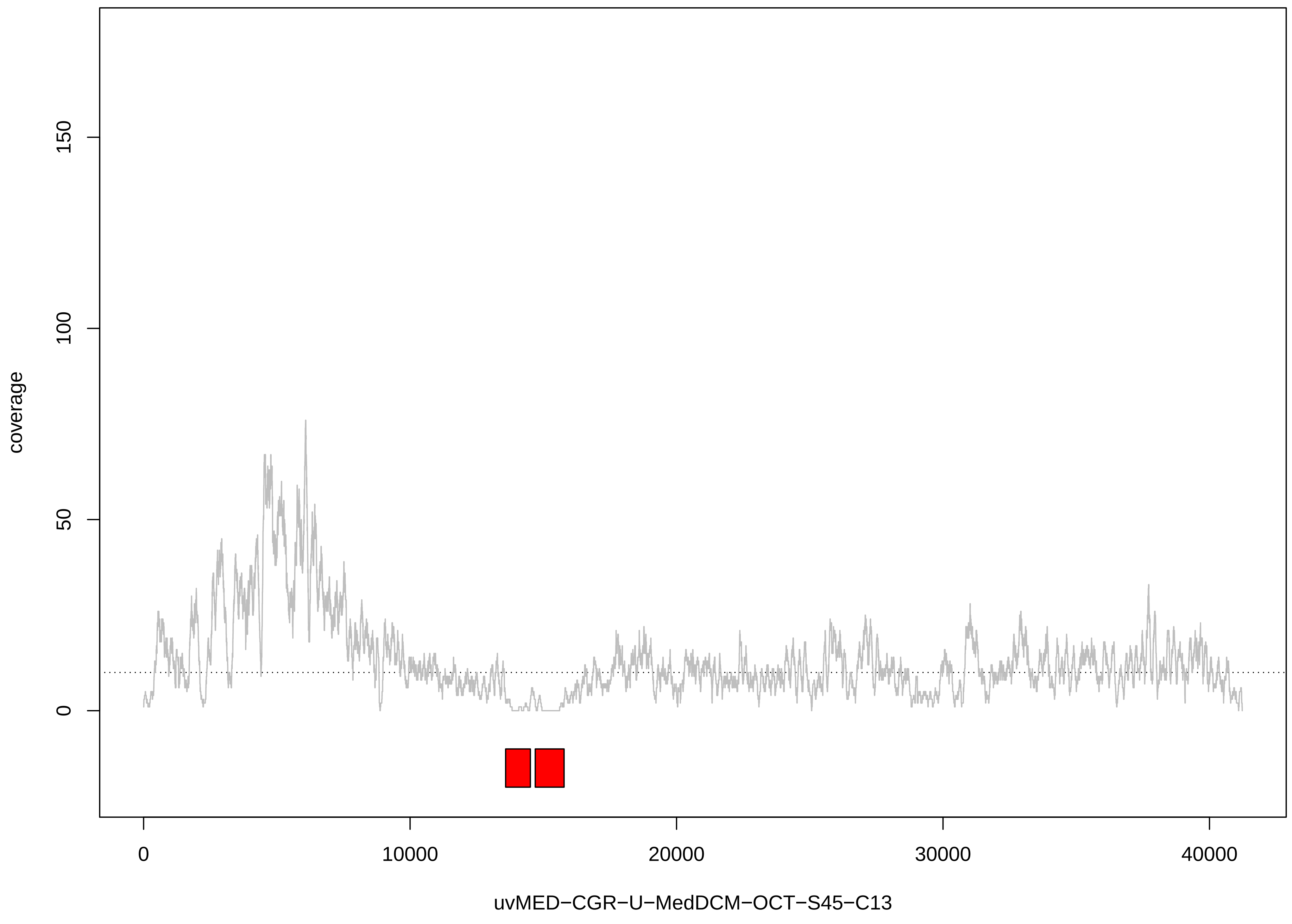
coverage
0
50
100
150
0
10000
20000
30000
40000
uvMED−CGR−U−MedDCM−OCT−S45−C13

(B): Coverage plots of 11 complete phage genome representatives (CGRs) against the metavirome. No MVIs were detected in all these CGRs. Gaps in the contigs (if present) are plotted as green rectangles. A dotted horizontal line indicates the median coverage of the contig.

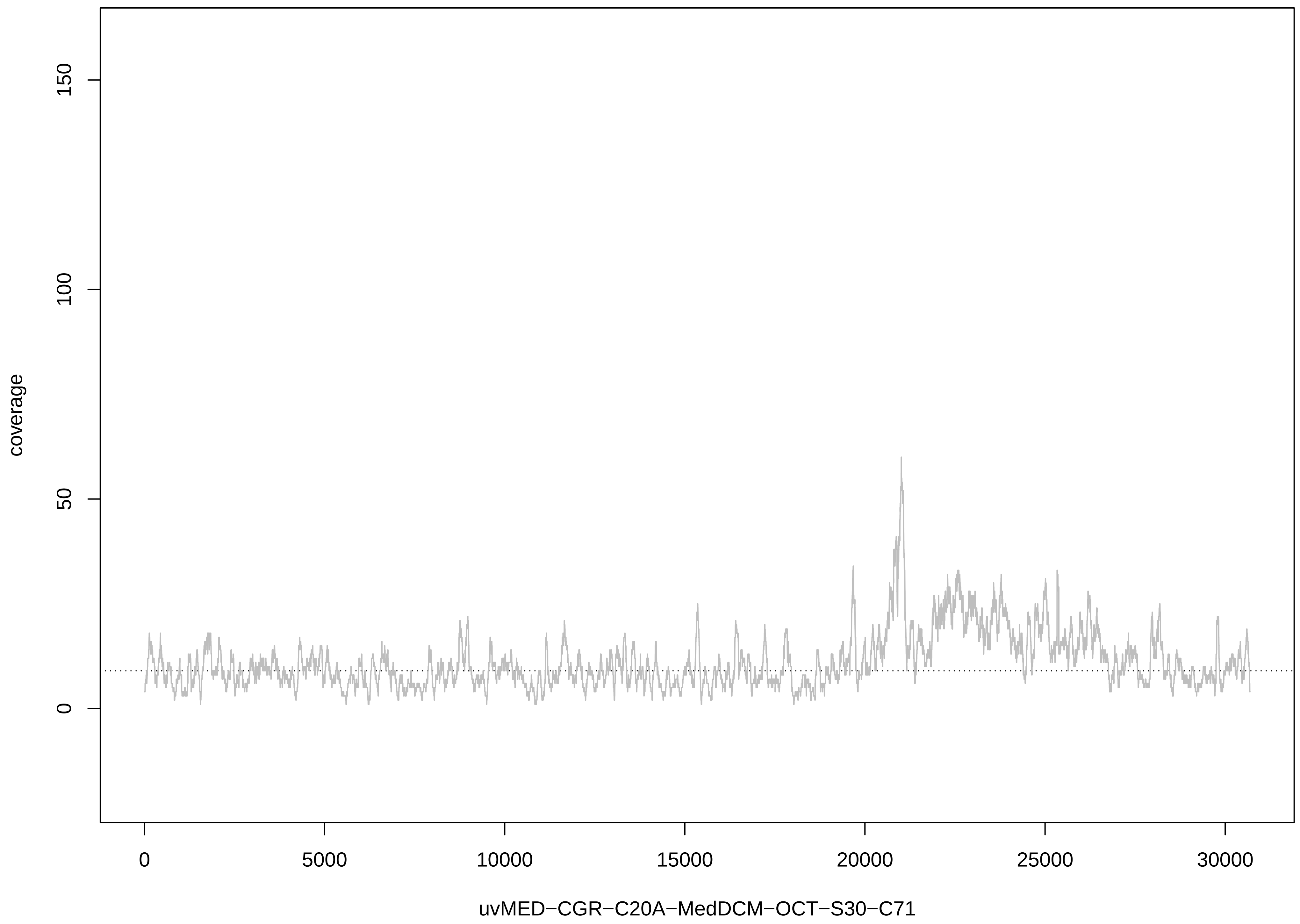

coverage
150
100
50
0
0
5000
10000
15000
20000
25000
30000
uvMED−CGR−C20A−MedDCM−OCT−S30−C71

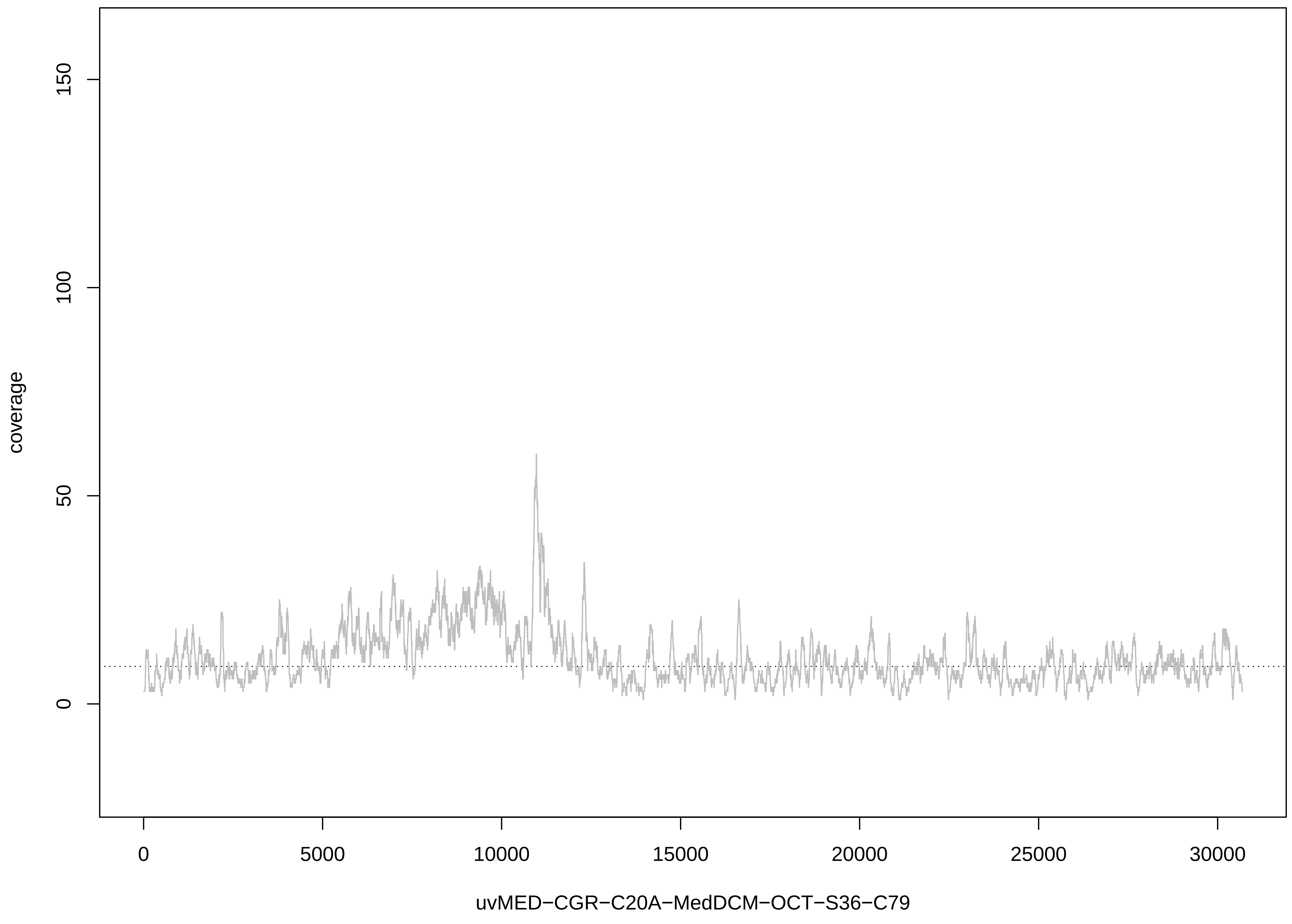

coverage
150
100
50
0
0
5000
10000
15000
20000
25000
30000
uvMED−CGR−C20A−MedDCM−OCT−S36−C79

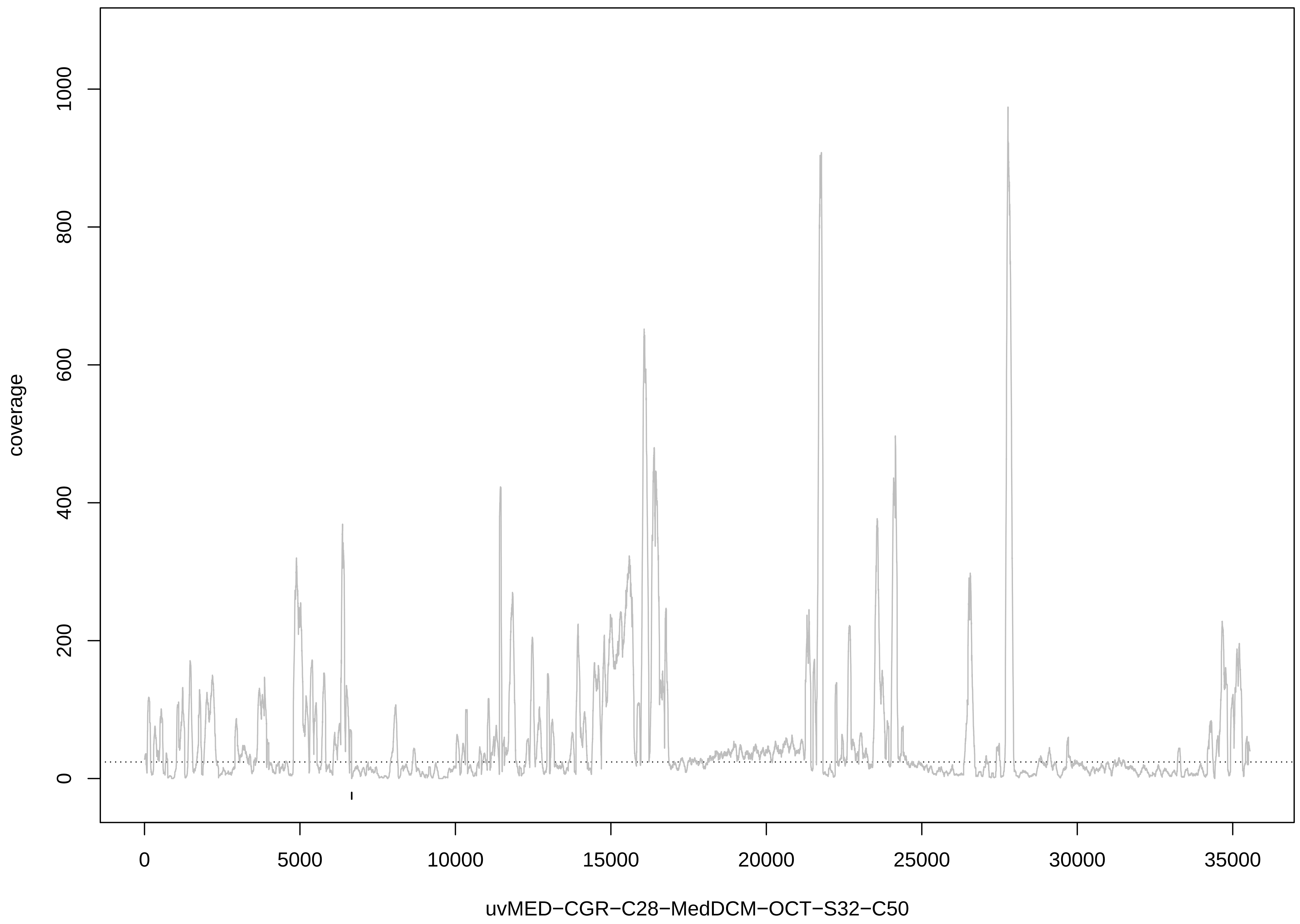
coverage
0
200
400
600
800
1000
0
5000
10000
15000
20000
25000
30000
35000
uvMED−CGR−C28−MedDCM−OCT−S32−C50

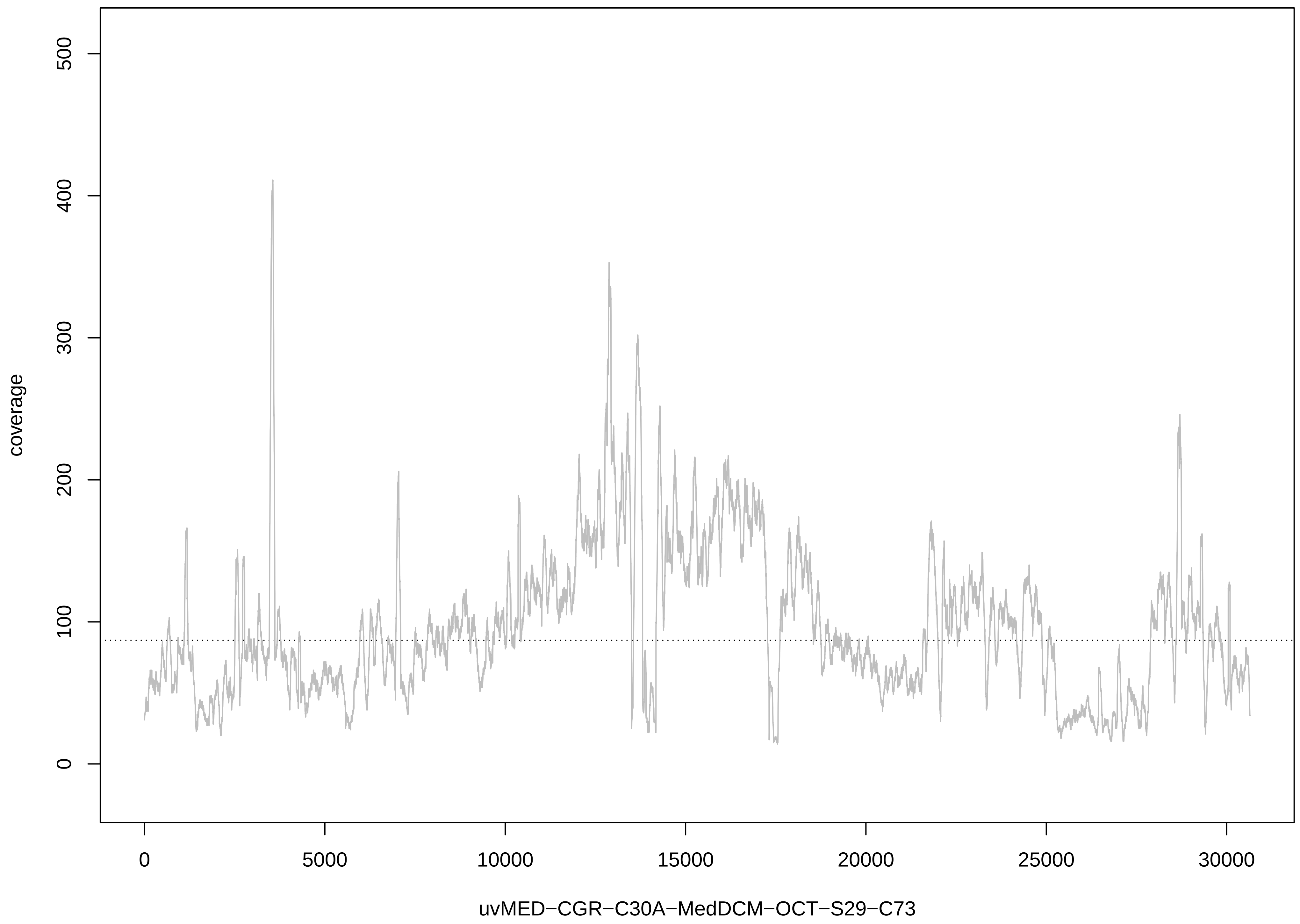

coverage
0
100
200
300
400
500
0
5000
10000
15000
20000
25000
30000
uvMED−CGR−C30A−MedDCM−OCT−S29−C73

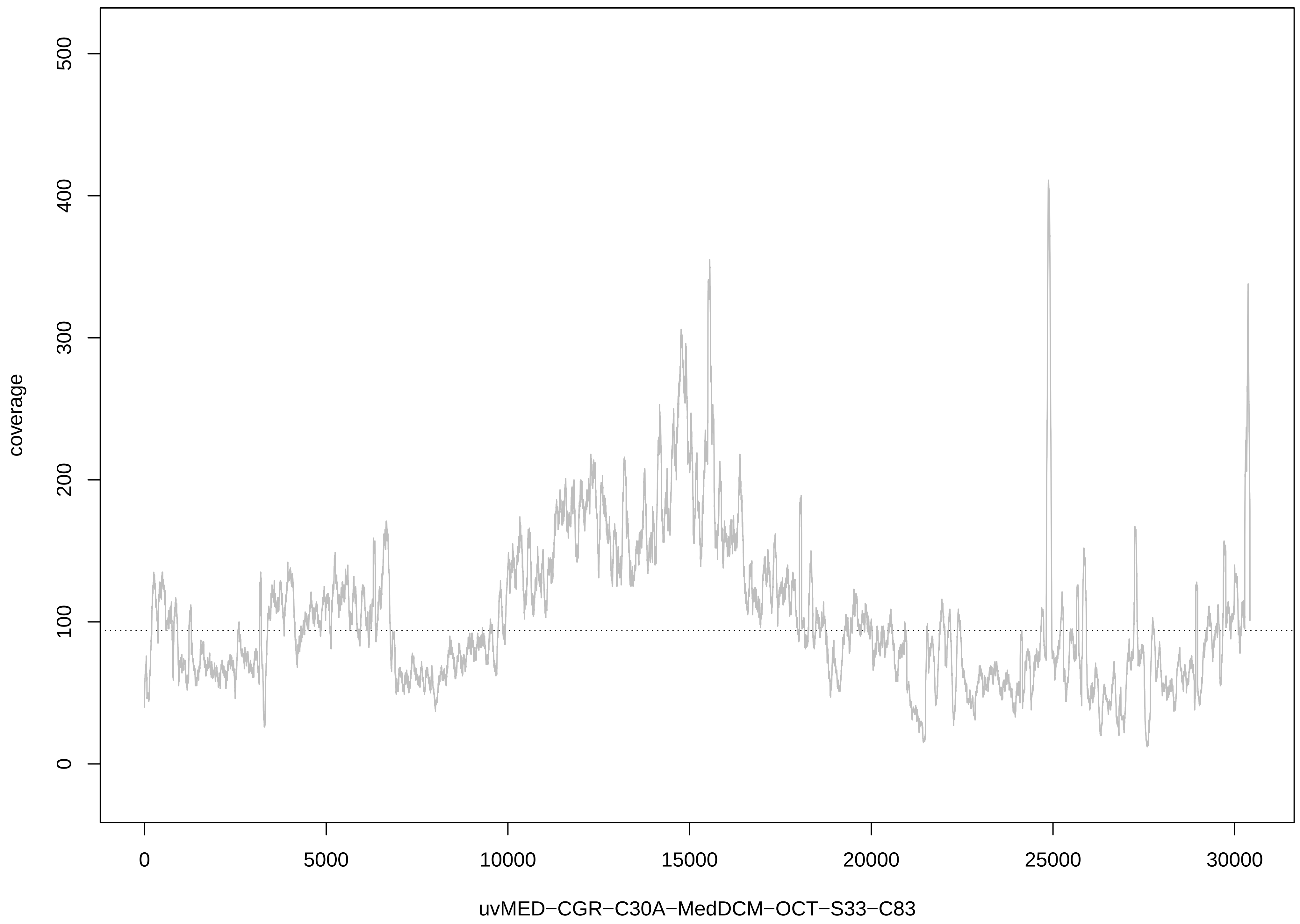

coverage
0
100
200
300
400
500
0
5000
10000
15000
20000
25000
30000
uvMED−CGR−C30A−MedDCM−OCT−S33−C83

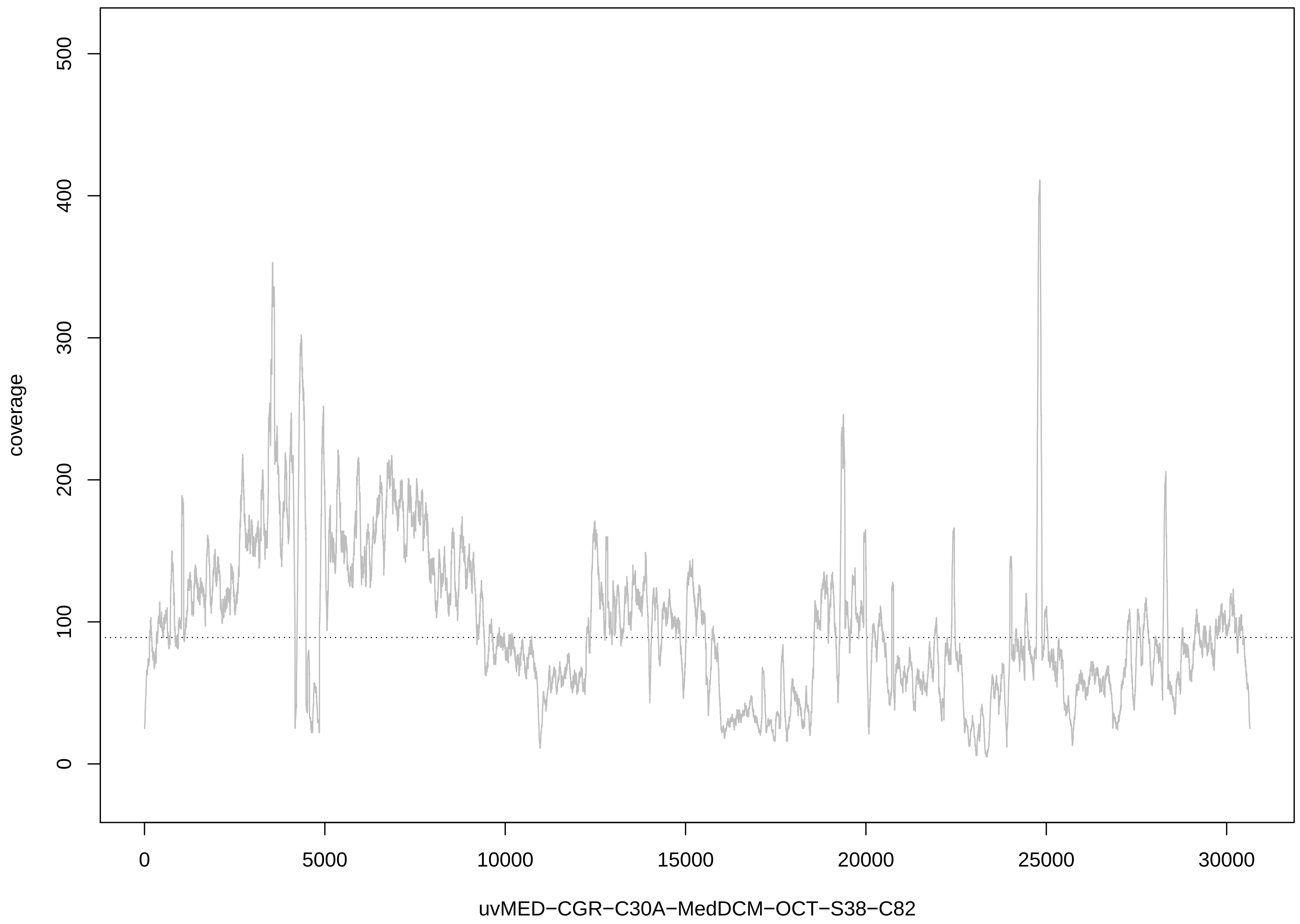

coverage
0
100
200
300
400
500
0
5000
10000
15000
20000
25000
30000
uvMED−CGR−C30A−MedDCM−OCT−S38−C82

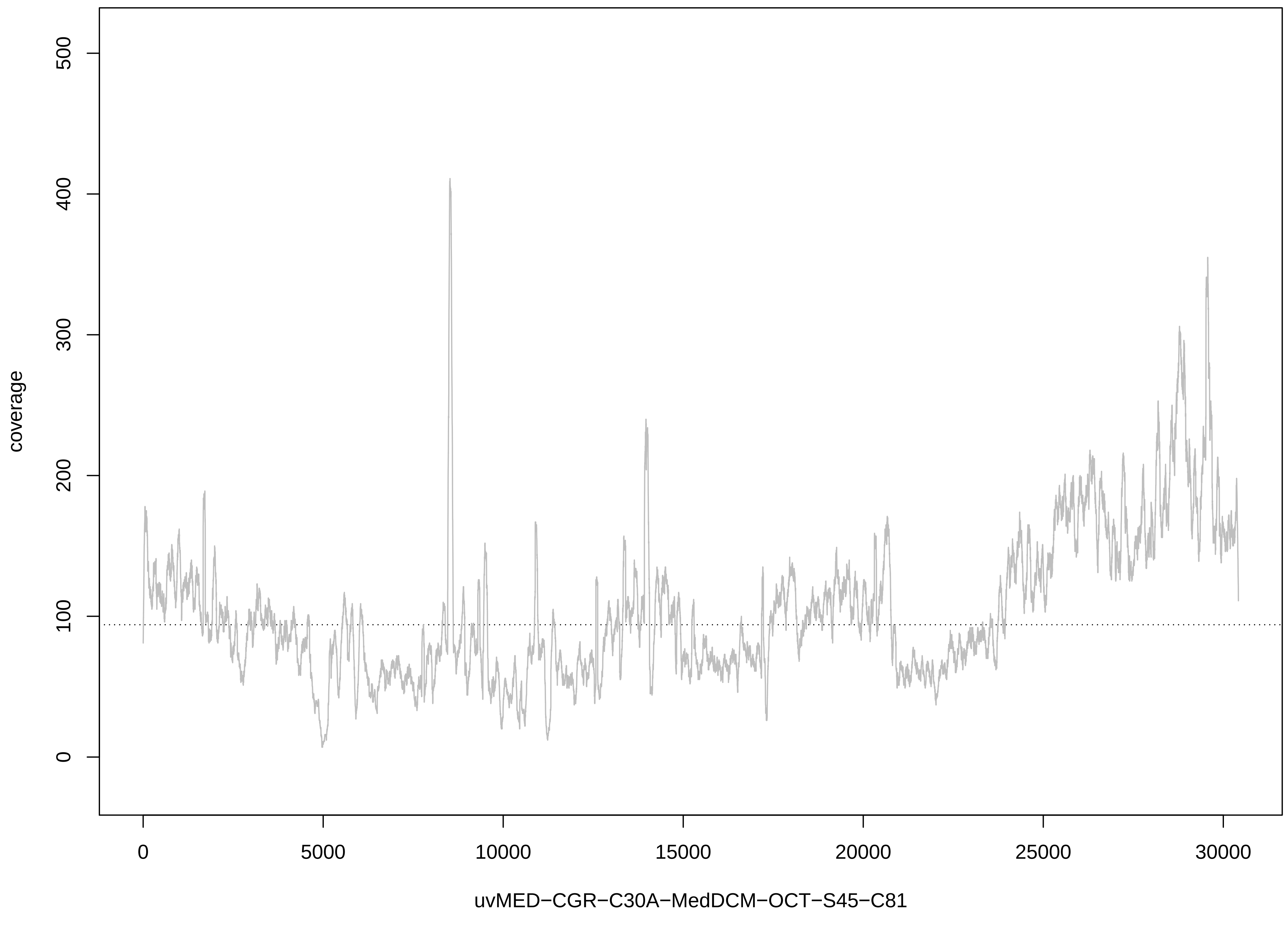

coverage
0
100
200
300
400
500
0
5000
10000
15000
20000
25000
30000
uvMED−CGR−C30A−MedDCM−OCT−S45−C81

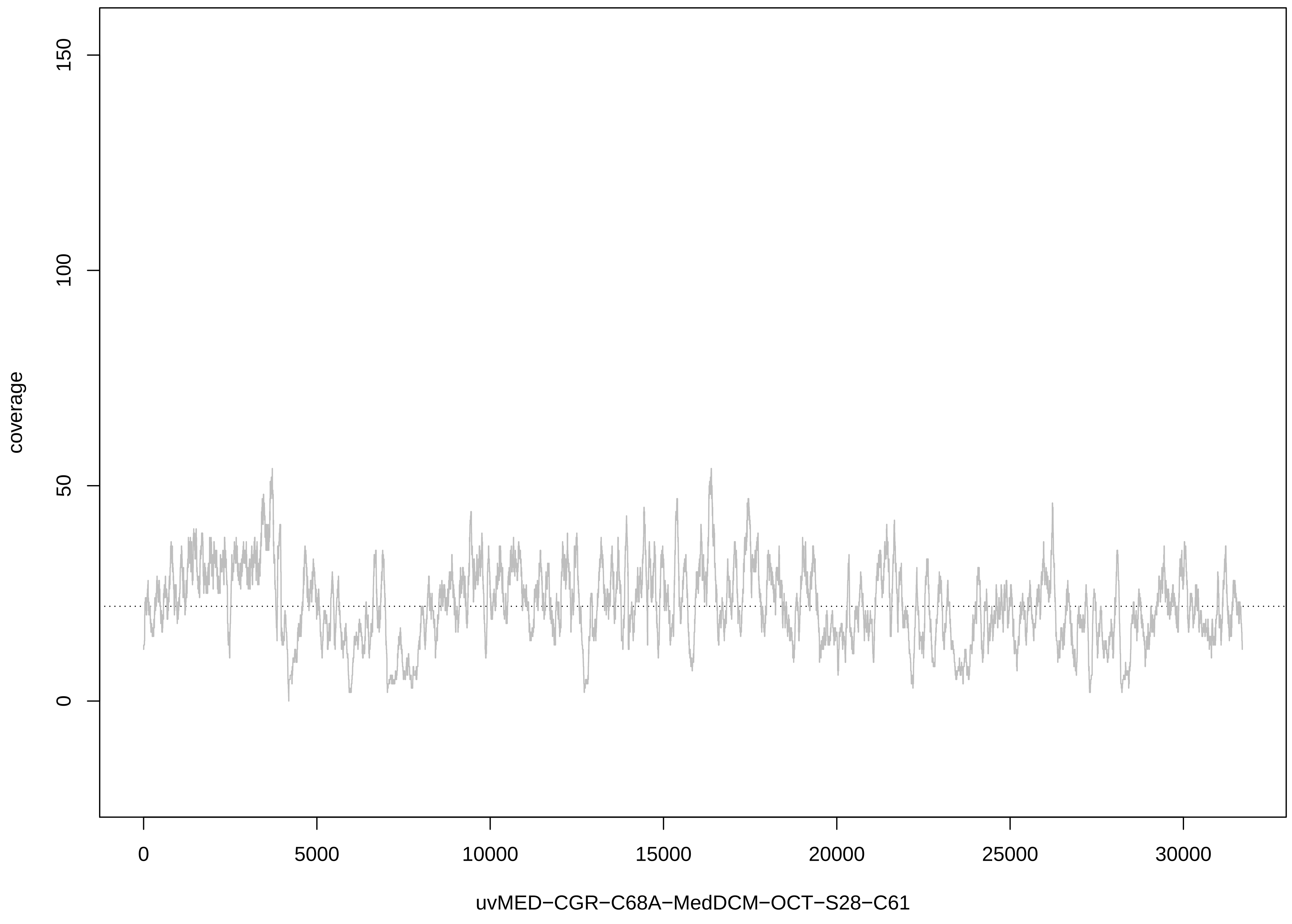
coverage
0
50
100
150
0
5000
10000
15000
20000
25000
30000
uvMED−CGR−C68A−MedDCM−OCT−S28−C61

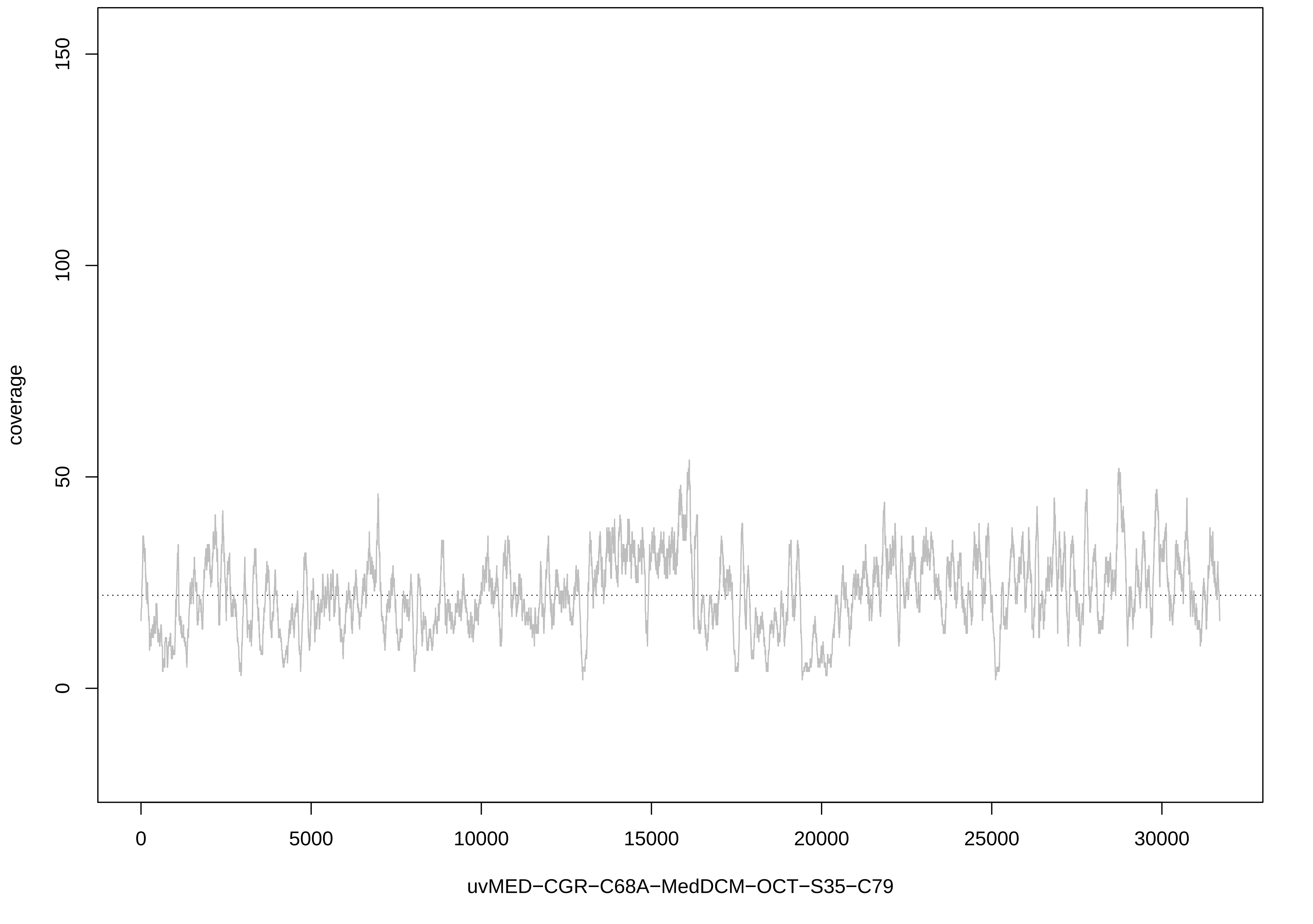

150
100
50
0
coverage
0
5000
10000
15000
20000
25000
30000
uvMED−CGR−C68A−MedDCM−OCT−S35−C79

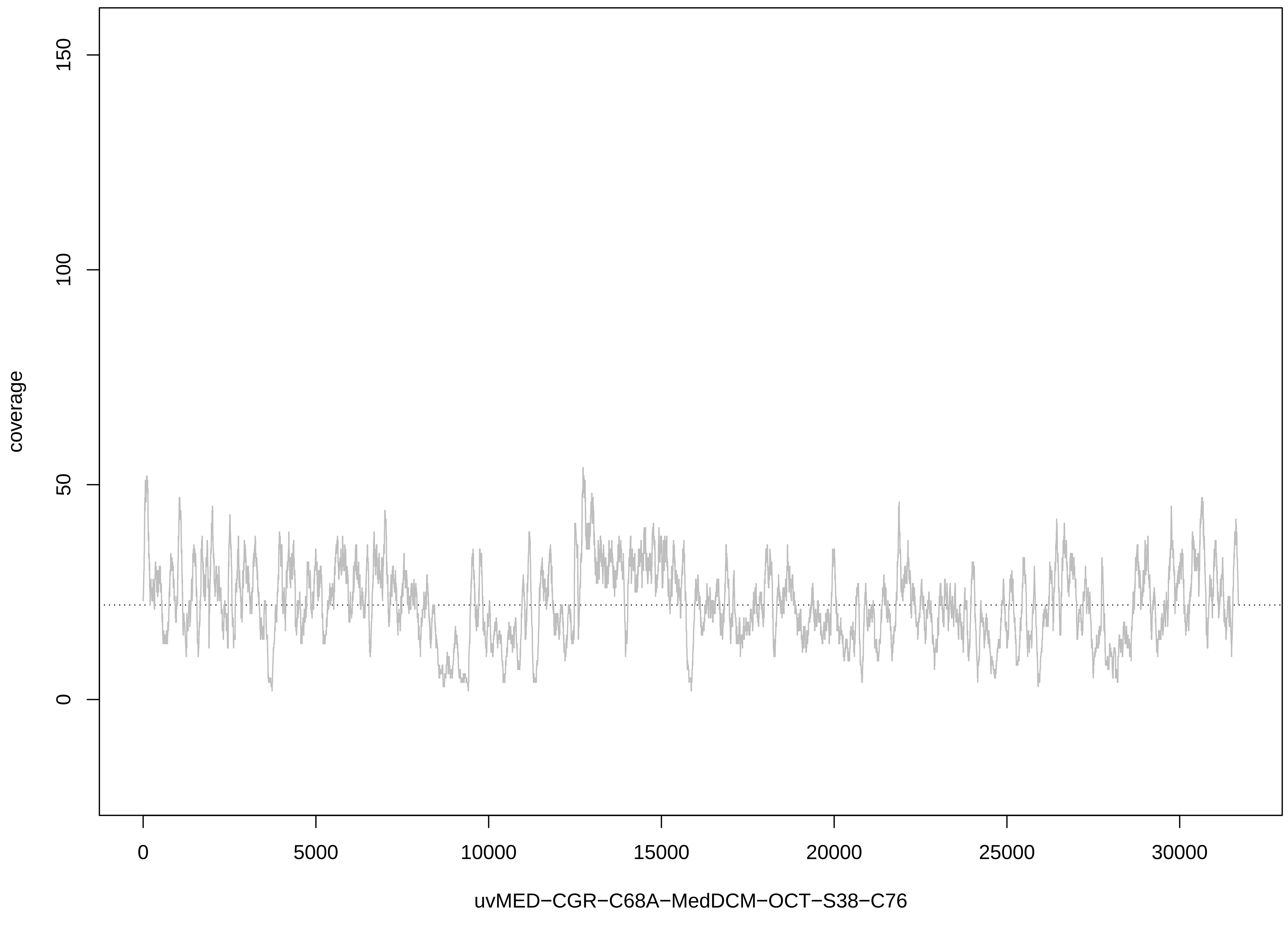

coverage
150
100
50
0
0
5000
10000
15000
20000
25000
30000
uvMED−CGR−C68A−MedDCM−OCT−S38−C76

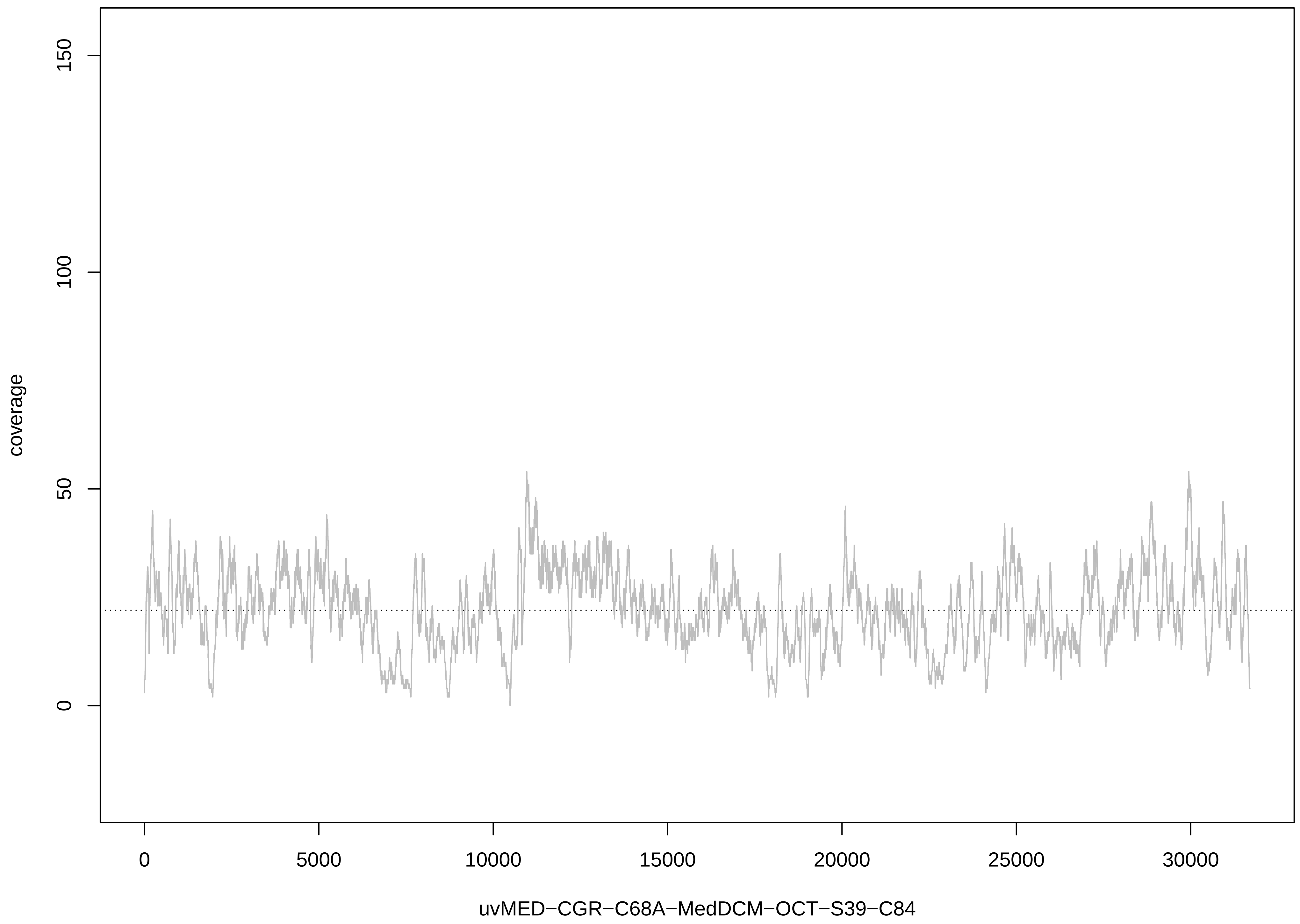
coverage
150
100
50
0
0
5000
10000
15000
20000
25000
30000
uvMED−CGR−C68A−MedDCM−OCT−S39−C84
